# Structure−Activity Relationships of New 1‐Aryl‐1H‐Indole Derivatives as SARS‐CoV‐2 Nsp13 Inhibitors

**DOI:** 10.1002/cmdc.202500205

**Published:** 2025-05-20

**Authors:** Valentina Noemi Madia, Roberta Emmolo, Elisa Patacchini, Donatella Amatore, Stefania Maloccu, Davide Ialongo, Aurora Albano, Giuseppe Ruggieri, Emanuele Cara, Laura Zarbo, Antonella Messore, Riccardo De Santis, Alessandra Amoroso, Florigio Lista, Francesca Esposito, Enzo Tramontano, Angela Corona, Roberto Di Santo, Roberta Costi

**Affiliations:** ^1^ Dipartimento di Chimica e Tecnologie del Farmaco Istituto Pasteur‐Fondazione Cenci Bolognetti “Sapienza” Università di Roma p.le Aldo Moro 5 I‐00185 Rome Italy; ^2^ Dipartimento di Scienze della Vita e dell’Ambiente Sezione biomedica Laboratorio di Virologia Molecolare Blocco E primo piano Università di Cagliari Cittadella Universitaria di Monserrato SS554 ‐09042 Monserrato (CA) Italia; ^3^ Istituto di Scienze Biomediche della Difesa 00184 Roma Italy; ^4^ Dottorato di Interesse Nazionale in One Health approaches to infectious diseases and life science research Dipartimento di Sanità Pubblica Medicina Sperimentale e Forense Università degli Studi di Pavia 27100 Pavia Italia; ^5^ Department of Life Science, Health, and Health Professions Link Campus University Via del Casale di San Pio V 44 I‐00165 Rome Italy; ^6^ Dipartimento di Sanità Pubblica e Malattie Infettive “Sapienza” Università di Roma 00161 Roma Italy

**Keywords:** antiviral agents, diketo acids, drug discovery, indole derivatives, nonstructural protein 13, SARS‐CoV‐2 inhibition, structure–activity relationships

## Abstract

It has been more than four years since the first report of SARS‐CoV‐2, the virus responsible for the coronavirus disease 2019 (COVID‐19) pandemic, the scientific community is focused on vaccine development in an exceptionally rapid time frame, as well as the evaluation of a wide range of potential treatments in clinical trials, a few of which have also reached the market. However, these drugs are characterized by several limits (including low response to treatment in some patients, low effectiveness against the new variants, severe side effects, etc.), thus underscoring the need to speed up the research. Among potential antiviral targets, the SARS‐CoV‐2 nonstructural protein 13 (nsp13) is highly promising thanks to its pivotal role in viral replication. Pursuing the studies on the development of nsp13 inhibitors, herein, the design, synthesis, and biological evaluation of new SARS‐CoV‐2 nsp13 inhibitors are reported. In general, the newly designed dikehexenoic derivatives are proven active against both the enzymatic activities showing measurable IC_50_ under 30 μM concentration, while the diketobutanoic series shows less promising results. Moreover, the tested compounds are capable of blocking viral replication without exerting cytotoxicity. Docking studies predict their binding into an allosteric pocket within the RecA2 domain.

## Graphical abstract

SARS‐CoV‐2 nsp13 is a promising target to develop effective antivirals. Pursuing the studies, new indolyl derivatives to deepen SARs are designed. The newly synthesized N‐aryl indoles are active vs both nsp13‐associated activities. They exert antiviral activity vs SARS‐CoV‐2 infected cells with no cytotoxicity. *In silico* studies predict their binding into an allosteric pocket.

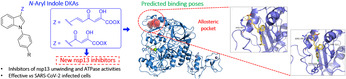

## 1 Introduction

The emergence of the novel coronavirus (CoV), known as severe acute respiratory syndrome coronavirus 2 (SARS‐CoV‐2), toward the end of 2019 posed an unparalleled global health crisis. Fast forward about four years, the Coronavirus Disease 2019 (COVID‐19) pandemic has gradually transitioned toward an endemic phase. However, the reality remains stark.^[^ ^1^ ^]^ As of November 2024, over 777 million confirmed COVID‐19 cases and over 7 million confirmed deaths have been reported globally.^[^ ^2^ ^]^ Moreover, the dynamic nature of the spike protein coupled with growing fatigue from vaccination, provide opportunities for the virus to persist and adapt, thus prolonging its endemic status.^[^ ^3^, ^4^ ^]^ Hence, the development of effective antiviral treatments emerges as a critical strategy in addressing the ongoing threat and potential future outbreaks. Indeed, engaging in prudent foresight today and addressing disease outbreaks and pandemics could facilitate a more efficient response in the future.

The discovery of direct antiviral agents against SARS‐CoV‐2 has proven much more challenging than developing vaccines, however, a few drugs have reached the market.^[^ ^5^ ^]^ The first was Remdesivir, a viral RNA‐dependent RNA polymerase (RdRp) inhibitor, repurposed from Ebola virus disease, that reported EC_50_ of 0.77 μM in Vero‐E6 assay vs SARS‐CoV‐2‐infected cells.^[^ ^6^ ^]^ However, its intravenous route of administration clearly limits its use to hospital settings.^[^ ^7^ ^]^ The other RdRp inhibitor, Molnupiravir, originally developed to treat influenza virus infections, was approved for oral use in people who have mild‐to‐moderate COVID‐19.^[^ ^8^ ^]^ Even so, the Food and Drug Administration (FDA) gave only the Emergency Use Authorization (EUA) so far while the European Medicines Agency refused the marketing authorization in June 2023.^[^ ^9^ ^]^ Paxlovid, a combination of the main protease (M^pro^) inhibitor Nirmatrelvir and the booster Ritonavir (an old anti‐HIV protease inhibitor, acting as CYP inhibitor) is an oral drug for adults who do not require supplemental oxygen.^[^ ^10^ ^]^ Unfortunately, the need to be coadministered with the booster highlights the drawbacks. Finally, Ensitrelvir is an innovative M^pro^ inhibitor,^[^ ^11^ ^]^ approved only in Japan^[^ ^12^ ^]^ and that remains an investigational drug for the FDA so far.^[^ ^13^, ^14^ ^]^ Moreover, drug‐resistant variants vs currently approved drugs have already emerged.^[^ ^15^, ^16^, ^17^ ^]^ Along with these small molecules, a series of monoclonal antibodies are also available but presenting a number of limitations,^[^ ^18^ ^]^ recently pushing the FDA to recommend against the use of most of them.^[^ ^19^ ^]^ In the current scenario, there is still an urgent need to develop new drugs to counter the looming threat of resistance and endowed with anti‐pan‐CoV activity to be also active against future emerging CoVs.

Analysis of mutations in SARS‐CoV‐2 and its genome comparison with previous CoVs has unveiled alternative targets characterized by notably reduced mutation rates and enhanced structural preservation. Among these are functional proteins susceptible to be targeted by small‐molecule inhibitors, presenting distinct advantages over vaccines in terms of formulation, production, storage, and administration ease.^[^ ^20^ ^]^ One of the most promising is the viral nonstructural protein 13 (nsp13). Indeed, pathogenic coronaviruses SARS‐CoV‐2, MERS‐CoV, and SARS‐CoV‐1 have nsp13 enzymes. They share a high degree of sequence similarity (84%–99%) and a close structural organization.^[^ ^21^ ^]^ For instance, SARS‐CoV‐1 and SARS‐CoV‐2 nsp13s demonstrate a 99.8% of sequence identity,^[^ ^22^, ^23^ ^]^ with only a single amino acid out 601 of difference between nsp13s of SARS‐CoV‐1 (I570) and SARS‐CoV‐2 (V570). Moreover, nsp13 is an enzyme that has a critical role in the viral life cycle, making it a highly attractive target for drug development.

Nsp13 belongs to the helicase superfamily 1 (SF1) that targets natural nucleotides as substrates when performing its ATPase activity. Nsp13 exploits the energy derived from the hydrolysis of ATP to unwind the double‐stranded nucleic acids in a 5’ to 3’ direction.^[^ ^24^ ^]^ Like all SF1 helicases, nsp13 shows two canonical RecA ATPase domains (RecA1 and RecA2).^[^ ^25^ ^]^ coupled with three domains unique to nidovirus helicases: the N‐terminal zinc‐binding domain, essential for the helicase activity, a stalk domain (SD), and a 1B domain.^[^ ^26^, ^27^ ^]^ Nsp13 plays a central and indispensable role in replication/transcription complex (RTC) formation. It engages the RTC where it interacts with the RdRP nsp12 which stimulates nsp13 activity.^[^ ^28^ ^]^ Consequently, nsp13 serves as a vital enzyme for viral replication and represents a validated target for drug discovery, whose inhibition offers a promising avenue for the development of antiviral agents.

**Figure 1:**
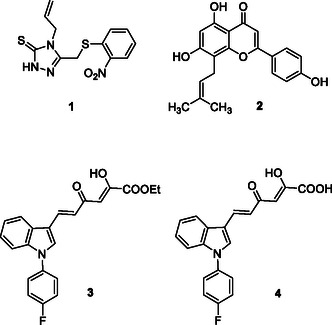
Structures of nsp13 inhibitors.

Recently, SARS‐CoV‐2 nsp13 has been actively explored as drug target, with reports describing small molecules as inhibitors of SARS‐CoV‐2 nsp13,^[^ ^29^, ^30^, ^31^, ^32^ ^]^ both of synthetic (as for SSYA10‐001,^[^ ^31^ ^]^ **1**, **Figure** **1**) or natural origin, such as in the case of licoflavone C (**2**, Figure 1).^[^ ^29^ ^]^ Most of them reported selectivity in inhibiting the nsp13‐associated unwinding in respect to the ATPase, and/or weak activity or inactivity vs SARS‐CoV‐2 infected cells. SSYA10‐001, for instance, was one of the first reported SARS‐CoV‐2 nsp13 inhibitor, showing IC_50_ vs unwinding of 7.5 μM, even though reporting a weak antiviral activity (EC_50_ = 81 μM).^[^ ^31^ ^]^ Similarly, the naturally occurring flavonoid **2** proved to allosterically block both SARS‐CoV‐2 nps13‐associated activities in the micromolar range (IC_50_ of 9.9 and 18.3 μM against unwinding and ATPase, respectively) but without antiviral activity (EC_50_ > 100 μM). Very recently, we described the first report of inhibitors active against both the SARS‐CoV‐2 nsp13‐associated activities and capable also of blocking viral replication in SARS‐CoV‐2 infected cells.^[^ ^33^ ^]^ We reported a small set of indolyl diketo acid (DKA) derivatives endowed with low micromolar inhibitory activity against both the unwinding and ATPase. Among them, compounds **3** and **4** (Figure 1) proved to be SARS‐CoV‐2 nsp13 dual inhibitors of both unwinding and ATPase, with the acid derivative **4** being the one with the best inhibitory profile, considering both the enzymatic and cellular activities (**3**: IC_50_ unwinding = 5.90 μM, IC_50_ ATPase = 13.60 μM, EC_50_ = 16.07 μM, CC_50_ > 264 μM; **4**: IC_50_ unwinding = 4.7 μM, IC_50_ ATPase = 8.2 μM, EC_50_ = 1.70 μM, CC_50_ > 264 μM). Furthermore, both **3** and **4** proved to be active also on other human CoVs such as HCoV229E and MERS‐CoV in the low micromolar and submicromolar range, respectively. The experimental investigation of the binding mode revealed ATP‐noncompetitive kinetics of inhibition, suggesting an allosteric binding mode that was further supported by molecular modeling predicting the binding into an allosteric conserved site within the RecA2 domain.

**Figure 2:**
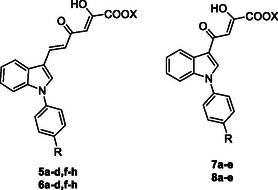
Structures of derivatives **5a–d,f–h, 6a–d,f–h, 7a–e,** and **8a–e**.

Pursuing this study, we decided to expand the structure–activity relationships (SARs) within this series of inhibitors. Therefore, we designed and synthesized new indolyl DKA derivatives structurally related to **3** and **4**.^[^ ^33^ ^]^ Here, we describe the design, synthesis, and biological evaluation of novel inhibitors targeting nsp13, with the aim to widen the pool of therapeutic agents capable of mitigating the impact of SARS‐CoV‐2. In detail, by keeping fixed the 3‐diketo acid indole moiety, we conceived a new series of derivatives (**5a–d,f–h, 6a–d,f–h, 7a–e** and **8a–**, **Figure** **2**) characterized by one or more of the following modifications: introduction of variously substituted phenyl rings on the nitrogen atom of the indole core, and/or shortening of the diketohexenoic branch into a diketobutanoic one. Specifically, the new derivatives were designed by replacement of the fluorine atom in the *p*‐fluorophenyl moiety of the hits with several substituents endowed with different steric or electronic properties.

## 2 Results and Discussion

### 2.1 Chemistry

**Scheme 1:**
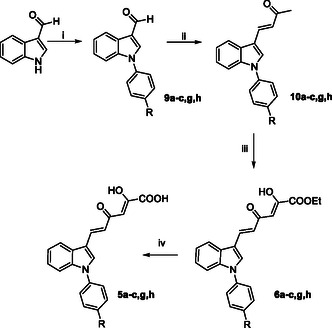
Synthetic route to **5a–c,g,h** and **6a–c,g,h** derivatives. Reagents and conditions: i) proper iodobenzene derivative, Cu_2_O, K_2_CO_3_, DMF dry, 160 °C, 24 h, 27%–76% yield; ii) acetone, 5 N NaOH, 50 °C, 24 h, 56%–90% yield; iii) diethyl oxalate, EtONa, THF dry, N_2_, room temp, 20 min–1.5 h, 61%–100% yield; and iv) 1N NaOH, 1:1 THF/EtOH, room temp, 15 min–1 h, 22%–90% yield.

Compounds **5d,f** and **6d,f** have been obtained as previously reported by us.^[^ ^34^, ^35^ ^]^ The synthesis of *N*‐phenyl diketohexenoic derivatives **5a–c,g,h** and **6a–c,g,h** was performed as reported in **Scheme** **1**. The synthetic pathway starts with the *N*‐arylation of the 1* H*‐indole‐3‐carboxaldehyde following an Ulmann‐type C—N cross‐coupling reaction with the properly substituted iodobenzene derivative using Cu_2_O as catalyst and K_2_CO_3_ as base, obtaining the *N*‐phenyl derivatives **9a–c,g,h**. Afterward, a crossed aldol condensation reaction with acetone in the presence of 5N NaOH furnished the enones **10a–c,g,h** that underwent a Claisen–Schmidt condensation with diethyl oxalate using freshly prepared sodium ethoxide as base, to give dikehexenoic ethyl esters **6a–c,g,h**. The subsequent base‐catalyzed hydrolysis using 1N NaOH afforded the corresponding acids **5a–c,g,h**.

**Scheme 2:**
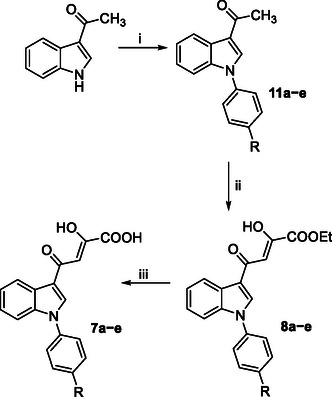
Synthetic route to **7a–e** and **8a–e** derivatives. Reagents and conditions: i) proper iodobenzene derivative, Cu_2_O, K_2_CO_3_, DMF dry, 160 °C, 24 h, 53%–83% yield; ii) diethyl oxalate, EtONa, THF dry, N_2_, room temp, 15–45 min, 56%–89% yield; and iii) 1N NaOH, 1:1 THF/EtOH, room temp, 15 min^−1^ h, 44%–89% yield.

*N*‐phenyl diketobutanoic derivatives **7a–e** and **8a–e** have been synthesized, as described in **Scheme** **2**. Commercially available 3‐acetylindole was reacted with the appropriate iodobenzene derivative in a similar fashion to that described in Scheme 1, obtaining *N*‐phenyl derivatives **11a–e** that were subsequently converted into the corresponding diketo esters **8a–e** via a Claisen–Schmidt condensation in the same conditions described above. Finally, an alkaline hydrolysis gave the corresponding acids **7a–e**.

### 2.2 Evaluation of Biological Activities

#### 2.2.1 *In Vitro* Screening for nsp13 Inhibitory Activity

All compounds **5a–d, f–h, 6a–d, f–h, 7a–e,** and **8a–e** were tested *in vitro* on both the SARS‐CoV‐2 nsp13 unwinding and ATPase‐associated activities (**Table** **1**), using compound **3** as positive control.^[^ ^33^ ^]^

**Table 1:** Inhibition of SARS‐CoV‐2 nsp13‐associated activities by the newly synthesized compounds 5a–d,f–h, 6a–d,f–h, 7a–e, and 8a–e.

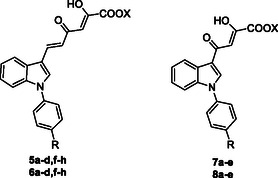				
Cpd	R	X	Activity in enzyme assay IC_50_ (μM)[a]	
			Unwinding BSA‐TCEP[b]	ATPase BSA‐TCEP[c]
**5a**	OCH_3_	H	2.83 ± 0.54	11.23 ± 1.37
**5b**	CH_3_	H	11.72 ± 0.94	5.125 ± 0.38
**5c**	*i*‐Pr	H	0.26 ± 0.01	8.43 ± 0.04
**5d**	H	H	11.83 ± 0.48	13.09 ± 0.37
**5f**	Cl	H	2.64 ± 0.61	20.95 ± 0.14
**5g**	CN	H	10.04 ± 2.07	13.62 ± 3.80
**5h**	CF_3_	H	0.21 ± 0.11	14.19 ± 1.60
**6a**	OCH_3_	Et	7.59 ± 1.50	10.40 ± 0.13
**6b**	CH_3_	Et	13.93 ± 1.50	8.64 ± 0.77
**6c**	*i*‐Pr	Et	4.16 ± 0.44	13.47 ± 3.34
**6d**	H	Et	3.77 ± 0.13	4.94 ± 0.21
**6f**	Cl	Et	2.05 ± 0.03	3.39 ± 0.11
**6g**	CN	Et	6.26 ± 0.65	2.35 ± 0.40
**6h**	CF_3_	Et	3.11 ± 0.55	1.21 ± 0.16
**7a**	OCH_3_	H	>30 (63.94%)[d]	>30 (68.69%)
**7b**	CH_3_	H	1.27 ± 0.29	>30 (70.83%)
**7c**	*i*‐Pr	H	0.70 ± 0.13	>30 (63.88%)
**7d**	H	H	>30 (75.09%)	>30 (102.12%)
**7e**	F	H	7.03 ± 1.94	>30 (99.3%)
**8a**	OCH_3_	Et	18.85 ± 0.22	19.06 ± 0.14
**8b**	CH_3_	Et	17.53 ± 1.37	18.63 ± 1.14
**8c**	*i*‐Pr	Et	8.04 ± 2.10	15.70 ± 0.10
**8d**	H	Et	13.35 ± 3.45	11.34 ± 0.89
**8e**	F	Et	12.89 ± 0.59	16.39 ± 3.10
**3** [e]	–	–	5.90 ± 0.30	13.60 ± 3.20

[a] Inhibitory concentration 50% (*μ*M) determined from dose–response curves.

[b] Experiments performed against SARS‐CoV‐2 nsp13‐associated unwinding activity.

[c] Experiments performed against SARS‐CoV‐2 nsp13‐associated ATPase activity.

[d] Percentage of enzymatic residual activity.

[e] From ref. [33]. The values are expressed as means ± the SD from two experiments, each performed in triplicate.

In general, the newly designed indolyl derivatives were proven active against both the enzymatic activities showing measurable IC_50_ under 30 μM concentration, with only 2 out of 24 tested compounds inactive vs both unwinding and ATPase, 2 inactive against unwinding and 5 inactive against ATPase activity. Among them, 3 compounds proved to be active in the submicromolar range and 7 derivatives showing inhibitory potencies about or lower than 5 μM against unwinding, while 5 compounds showed IC_50_ values about or lower than 5 μM vs ATPase (out of the 24 compounds tested). *N*‐phenyl diketohexenoic derivatives (series **5** and **6**) proved to be highly promising, as it can be observed with compounds **5c** and **5h** (IC_50_ = 0.26 and 0.21 μM, respectively), endowed with the best inhibitory activity vs unwinding among all the newly synthesized compounds, and with **6h** characterized by the best IC_50_ value against ATPase (IC_50_ = 1.21 μM).

All the *N*‐phenyl diketohexenoic compounds proved to be active against both the enzymatic activities, with all of them showing IC_50_ values between 0.21 μM (**5h**) and 11.83 μM (**5d**). More in detail, 8 derivatives (**5a,c,f,h** and **6c,d,f,h**) out of 14 tested showed high inhibitory activities, with IC_50_ values lower than 5 μM, 3 compounds (**5g** and **6a,g**) proved to be active with 5 μM < IC_5_ < 10, and 3 compounds (**5b,d** and **6b**) reported inhibitory activity with IC_50_ values > 10 μM. As concerns ATPase, all the compounds of this series were active up to the tested concentrations, with IC_50_s in the range 1.21 (**6h**)–20.95 μM (**5f**). Specifically, 4 derivatives (**6d,f,g,h**) out of 14 tested showed high inhibitory activities, with IC_50_ values lower than 5 μM, 3 compounds (**5b,c** and **6b**) proved to be active with 5 μM < IC_50_ < 10, and 7 (**5a,d,f–h** and **6a,c**) reported inhibitory activity with IC_50_ values > 10 μM. The most active compounds against unwinding were **5c** and **5h** (IC_50_ of 0.26 and 0.21 μM, respectively) that showed also 70‐fold and 30‐fold selectivity over ATPase, respectively. The best‐acting compound vs ATPase was **6h** (IC_50_ of 1.21 μM), that proved to be also the best dual inhibitors along with compound **6f** (IC_50_ unwinding = 2.05 μM, IC_50_ ATPase = 3.39 μM). In the light of the above, we can state that *N*‐phenyl diketohexenoic derivatives are generally more active against unwinding. Moreover, we observed the trend according to which the ester derivatives are, overall, more active than the acid counterparts, even though some exceptions can be noted as in the case of acid **5c** being more active than the ester counterpart and for the couples **5a–6a, 5b–6b**, and **5h–6h** (where the acids are more potent than the corresponding esters but only for one activity between unwinding or ATPase).

The introduction of electron‐withdrawing groups or electron‐donating ones in para position of the phenyl ring led to comparable activity values whether for the inhibition of the unwinding, ATPase, or both activities. It is worthy of note that about half of the acid derivatives of series **5** were more potent than the reference compound **4** vs both enzymatic activities, while, among the esters, more than half or even all of them were more active against unwinding or ATPase, respectively, than compound **3**.

*N*‐phenyl diketohexenoic indoles (series **5** and **6**) showed better inhibitory profiles than the diketobutanoic ones (series **7** and **8**), especially vs ATPase. Indeed, within series **7** and **8**, 2 compounds out of 10 were inactive and the others showed IC_50_s in the range 0.70–18.85 μM against unwinding, while against ATPase 5 derivatives out of 10 were inactive and 7 were active in the range 11.34–19.06 μM. Regarding the unwinding, only 2 compounds showed inhibition up to 5 μM, 2 compounds in the range 5 μM < IC_50_ < 10, and 5 compounds reported IC_50_ values > 10 μM. Regarding the ATPase, none of them showed inhibition up to 5 μM or in the range 5 μM < IC_50_ < 10, and 5 compounds reported IC_50_ values > 10 μM. It is evident, therefore, that these compounds are overall more active against the unwinding. The most active compound against unwinding was **7c** (IC_50_ of 0.70 μM) and the best‐acting compound vs ATPase was **8d** (IC_50_ = 11.34 μM), that proved to be the best dual inhibitor (IC_50_ unwinding = 13.35 μM, IC_50_ ATPase = 11.34 μM). Compounds **7b,c,e** were selective inhibitors, showing no activity vs ATPase up to the maximum concentration tested.

Also for this series of compounds, we observed the trend according to which the ester derivatives are, overall, more active than the acid counterparts, especially for the ATPase inhibition (all the acid compounds were, indeed, inactive), even though for the unwinding inhibition the acids **7b,c,e** were more active than the corresponding esters.

The introduction of electron‐withdrawing groups or electron‐donating ones in para position of the phenyl ring led to comparable activity values whether for the inhibition of the unwinding, ATPase, or both activities, as also observed for *N*‐phenyl diketohexenoic indoles.

**Figure 3:**
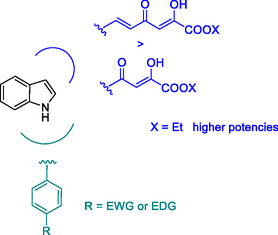
Chemical features of indolyl DKAs modulating their anti‐nsp13 properties.

Altogether, combining our previous results with the new insights gained from the present study, we were then able to describe the structural features that a compound should hold to achieve nsp13 inhibition with respect to both its associated enzymatic activities (**Figure** **3**).

In this regard, the indolyl scaffold is a good starting point since it can be easily functionalized with different chemical groups. On this scaffold, the introduction at 3‐position of a 4‐ or 6‐carbons DKA/diketoester branch has led to many derivatives endowed with promising inhibitory profile on the enzymatic target. In particular, the presence of the diketoester side chain generally improves the inhibition of the unwinding, ATPase, or both activities. Specifically, the presence of a diketohexenoic branch is amenable for higher potencies. In addition, the introduction of variously substituted phenyl rings in position 1 of the indole core confers good inhibitory profiles against unwinding, ATPase, or both, even though slightly better results in inhibiting unwinding were observed. It can be also noticed that various groups endowed with electron‐withdrawing/donating or steric properties can be introduced in para position of the phenyl ring.

#### 2.2.2 Antiviral Activity

The best‐acting compound against both the enzymatic activities (**6h**) and the best inhibitors against the unwinding (**5h**) or against ATPase (**6g**) were chosen to investigate their ability to block SARS‐CoV‐2 replication in infected cells. Compound **5c** was selected as the acid compound with better activity vs unwinding with respect to the ATPase and compound **6f** as the ester derivative with a comparable activity against both. Moreover, compound **7c** was also added being the most potent compound active among the diketobutanoic derivatives and selective vs the unwinding activity.

**Figure 4:**
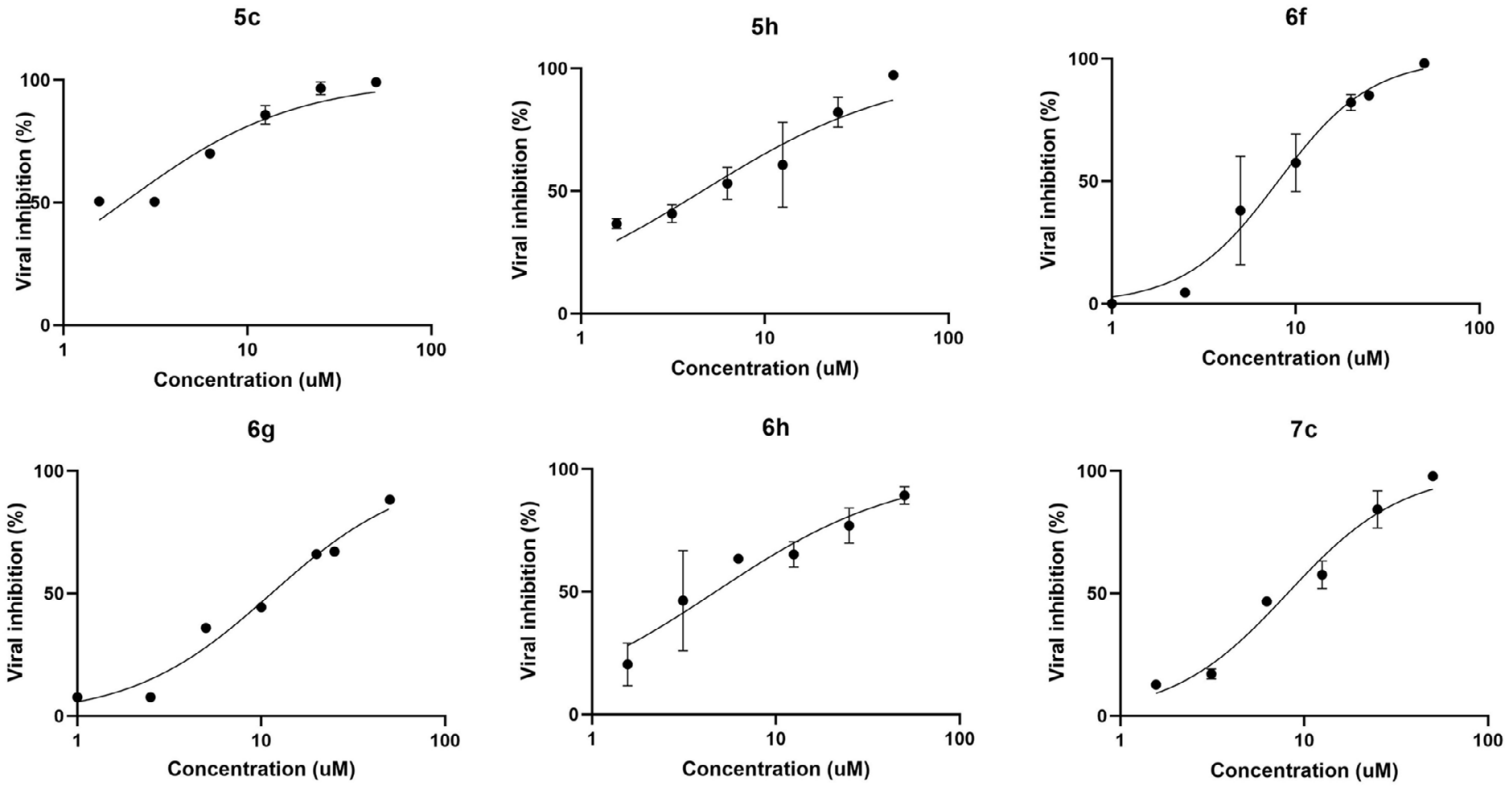
Antiviral effect of **5c,h,** **6f–h,** and **7c** against SARS‐CoV‐2. SARS‐CoV‐2 infected cells were treated with the compounds at concentrations ranging from 1 to 50 μM, and viral titer inhibition was calculated by plaque assay. Values are expressed as means ± the SD from three experiments, each performed in triplicate (*n* = 9).

In the first set of experiments, the effect of compounds on Vero cell viability was evaluated. To this end, confluent monolayers of cells were treated with different concentrations (100–10 μM) of exemplificative indolyl DKA derivatives for 24, 48, and 72 h. Microscopic examination showed no significant mortality in cells treated with the compounds at a concentration less than 50 μM while marked cytopathic effects were observed at concentration of 100 μM. Based on these results, the antiviral activity of the DKA derivatives against SARS‐CoV‐2 was tested through the plaque assay by evaluating the compounds at concentrations ranging from 1 to 50 μM (**Figure** **4**). Untreated‐infected cells were used as a positive control of viral infection (10^3^ pfu mL^−1^). The results indicate that **5c,h, 6f–h**, and **7c** inhibit SARS‐CoV‐2 with varying degrees of effectiveness (**Table** **2**).

**Table 2:** Antiviral activity against SARS‐CoV‐2 activities of derivatives 5c,h, 6f–h, 7c.

Cpd	EC_50_ (μM)[a]	CC_50_ (μM)[b]	SI[d]
**5c**	2.12 ± 0.67	>100	>47
**5h**	4.56 ± 1.86	>100	>21
**6f**	8.00 ± 2.19	>100	>12
**6g**	11.78 ± 7.45	>100	>8
**6h**	4.69 ± 1.80	>100	>21
**7c**	8.10 ± 1.37	>100	>12
**Camptothecin**	–	11.09 ± 0.16	–
**3** [c]	16.07 ± 3.25	>264	>16

[a] Compound concentration required to reduce the SARS‐CoV‐2 replication in Vero cells by 50% (*μ*M), expressed as means ± the SD from three experiments, each performed in triplicate (*n* = 9).

[b] Compound concentration required to reduce Vero cells viability by 50% (*μ*M).

[c] From reference.^[^ ^33^ ^]^

[d] SI = CC_50_/EC_50_.

To assess the *in vitro* inhibition potency of the selected compounds, the 50% inhibitory concentration (EC_50_) for each compound was calculated from the experimental curves to evaluate the *in vitro* inhibitory activity of the compounds. The values reported in Table 2 showed that in general the tested derivatives were more active than the hit **3**. In particular, the acid compounds of series 5 (**5c** and **5h**) proved to be the best acting ones in inhibiting SARS‐CoV‐2 replication, showing EC_50_ of 2.12 and 4.56 μM, respectively. In contrast, ester derivatives of series 6 reported EC_50_ values in the range 4.69–11.78 (**6f:** EC_50_ = 8.00 μM; **6g**: EC_50_ = 11.78 μM; **6h**: EC_50_ = 4.69 μM). Interestingly, the diketobutanoic derivative **7c** showed comparable inhibitory activity (EC_50_ = 8.10 μM). The 95% confidence interval (CI) for each compound was: **5c**: 1.460**–**2.789; **5h**: 2.882**–**6.620; **6f**: 7.018**–**9.208; **6g**: 8.589**–**16.04; **6h**: 3.117**–**6.726; and **7c**: 6.842–9.589.

### 2.3 Molecular Docking Prediction

A molecular docking protocol was employed to predict the potential binding mode of the DKA compounds on the nsp13 complex. Following the indication of the binding within the RecA2 domain as the most supported by our previously reported experimental findings,^[^ ^33^ ^]^ we hypothesized the most important residues for both unwinding and ATPase inhibition performing docking procedures on **5c**, **5h**, **6f,** and **6h**.

In general, the newly designed indolyl derivatives were proven active against both the enzymatic activities showing measurable IC_50_ under 30 μM concentration. Among them, the *N*‐phenyl diketohexenoic derivatives (series **5** and **6**) proved to be highly promising, as it can be observed with compounds **5b** and **5d** (IC_50_ = 0.21 and 0.26 μM, respectively), endowed with the best inhibitory activity vs unwinding among all the newly synthesized compounds. The best‐performing compound against ATPase was **6b** (IC_50_ of 1.21 μM), that proved to be also the best dual inhibitors along with compound **6g** (IC_50_ unwinding = 2.05 μM, IC_50_ ATPase = 3.39 μM).

To elucidate the inhibitory properties of these newly synthesized indolyl DKA derivatives at the atomic level, molecular modeling studies were attended. The 3D model of the SARS‐CoV‐2 nsp13 helicase protein used in this study is the previously published by our group.^[^ ^33^ ^]^

**Figure 5:**
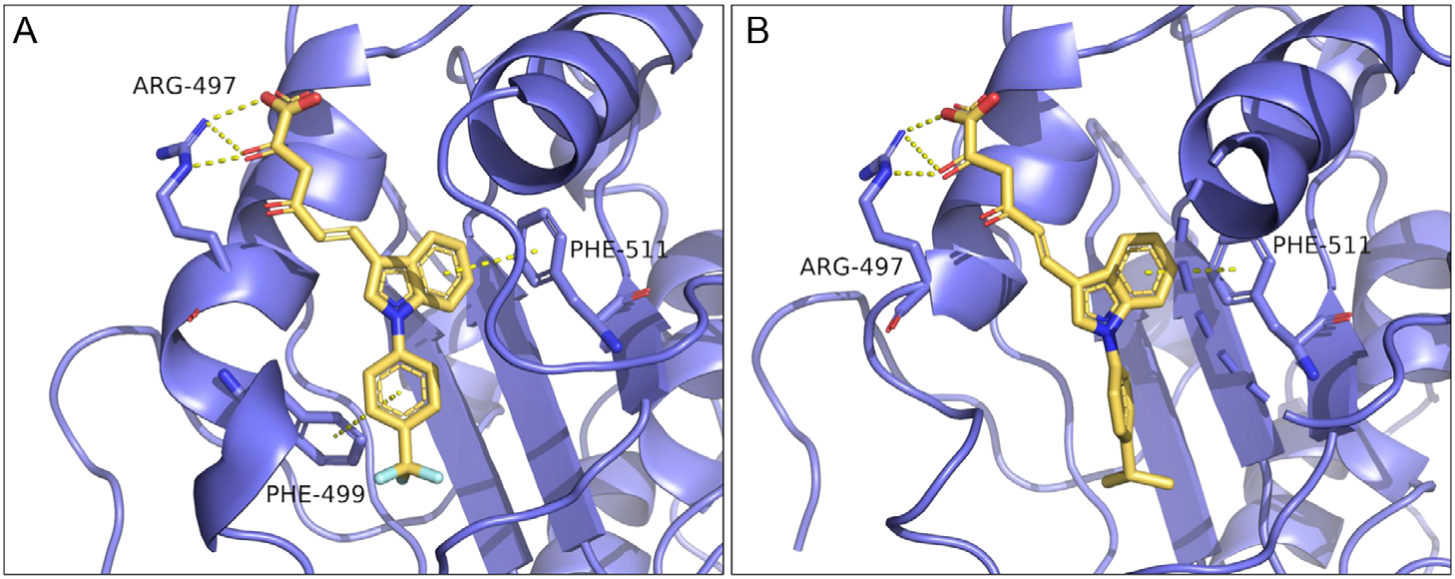
Predicted binding modes for **5h** A) and **5c** B).

After generating the model, docking calculations were attained for **5h** and **5c**, resulting in the binding pose depicted in **Figure** **5**.

Compounds **5h** (Figure 5A) and **5c** (Figure 5B) exhibited a similar binding mode, with the indole ring establishing a π–π stacking with Phe511.

Moreover, **5h** (Figure 5A) showed an interesting additional interaction. Indeed, this derivative can interact via another π–π stacking with Phe499 thanks to the presence of CF_3_ moiety, which is an electron‐withdrawing substituent capable of stabilizing this type of interaction.

Both compounds interact via DKA portion with basic residues Arg497 through salt bridges and H‐bond interactions. This finding is consistent with the *in vitro* biological assays, in which the ester counterparts **6h** and **6c** are less active against the unwinding than the acid ones **5h** and **5c**, respectively.

**Figure 6:**
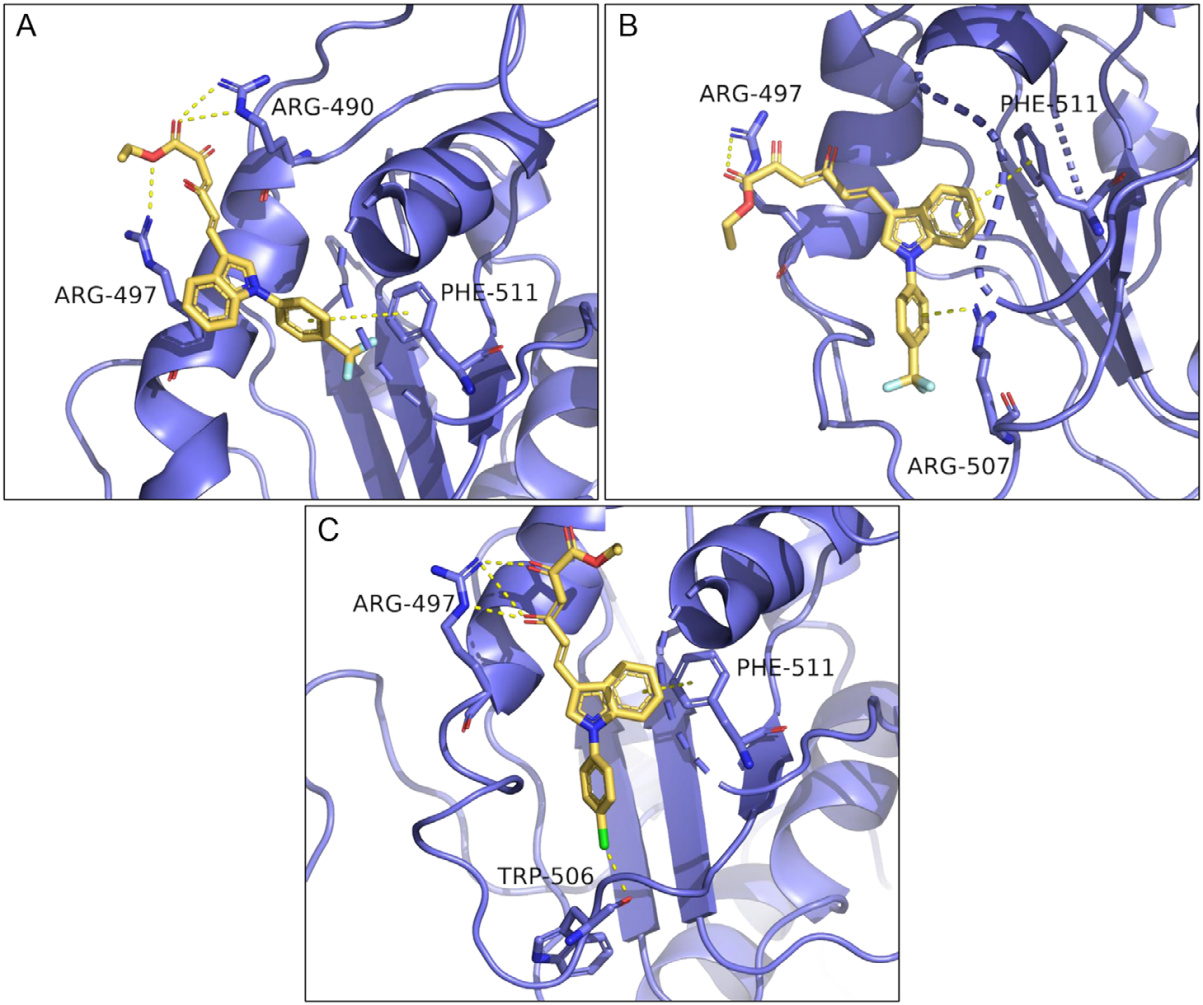
Predicted binding modes for **6h** A,B) and **6f** C).

As concerns ATPase, **6h** proved to be the most active compound, which is also the best dual inhibitor along with compound **6f**. To predict their binding poses, the same SARS‐CoV‐2 nsp13 helicase model was used to perform docking calculations, and the corresponding results are shown in **Figure** **6**.

These derivatives showed interesting binding poses. It is possible to observe that **6h** (Figure 6A,B) and **6f** (Figure 6C) are able to anchor themselves to RecA2 domain interacting via π–π stacking interactions with Phe511. It is worthy of note that this residue is the same involved into a π–π stacking interactions with the indole portions of **5h** (Figure 5A) and **5c** (Figure 5B).

About **6h**, this compound showed two different binding modes (Figure 6A,B). Indeed, the longer diketoester branch and, simultaneously, the presence of the electron‐withdrawing CF_3_ moiety allow the molecule either to establish a π–π interactions between the N‐aryl group and with Phe511 (Figure 6A) or a cation‐π interactions with Arg507 (Figure 6B). For the acid counterpart **5h** (Figure 5A), the shorter DKA chain and the stronger nature of the salt bridge bond only allow the stacking between indole and Phe511. However, this kind of binding pose was obtained also for **6h** (Figure 6B). The two binding modes showed by **6h**, equivalent in terms of probability, are both possible. We hypothesized that the pronounced inhibitory activity toward the ATPase of **6h** is due to this duality.

Regarding **6f**, it is interesting to note how the presence of chlorine atom anchors the molecule in a single possible binding pose, establishing a halogen bond with the backbone of Trp506.

In addition to these contacts, all the compounds interact via diketo ester portion with Arg497 through H‐bond interactions.

It bears mentioning that the poses obtained for **6h** (Figure 6A,B) are very similar to those obtained for **6g** (Figure S62 in the Supporting Information). Indeed, these two derivatives are both diketohexenoic esters and only differ in one substituent (Table 1). However, the *p*‐CN‐benzyl moiety of **6g** has characteristics such as polarity and electron‐withdrawing capacity comparable to that of *p*‐CF_3_‐benzyl moiety of **6h**. Considering this, it is not surprising the comparable in cellulo activity of these two compounds (Table 2).

In light of these results, we speculated that: (1) Phe511 is the most important residue for unwinding inhibition (Figure 5A,B and 6A‐C); (2) interactions with Arg507 or nearby residues such as Trp506 are essential for ATPase inhibition (Figure 6B,C).

### 2.4 Evaluation of Inhibition Type

**Figure 7:**
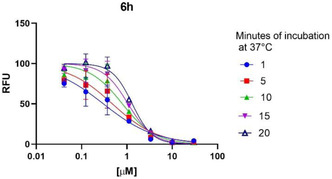
Mode‐of‐action assay for compound **6h**. The values are expressed as means ± the SD from a single experiment in triplicate.

To determine whether the compounds act as covalent/noncovalent inhibitors, we performed additional assays on the most promising compound **6h**. To determine the incubation‐dependent potency,^[^ ^36^ ^]^ that should lead to a decrease in IC_50_ values in case of covalent inhibition, the compound activity was monitored checking the amount of reaction products at different time‐points along the incubation time with the inhibitor: 1, 5, 10, 15, and 20 min after the activation of the reaction (**Figure** **7**). The time‐increased IC_50_ values obtained (0.30; 0.48; 0.79; 1.06 and 1.31 μM, respectively) indicate that the nature of the inhibition is noncovalent. Moreover, extending the preincubation time of compound and enzyme before the activation of the reaction with the substrate, to +20 min and +50 min at room temperature did not lead to increasing in inhibitory potency, further confirming the hypothesis.

## 3 Conclusion

The recent pandemic of COVID‐19, caused by SARS‐CoV‐2, rapidly spread worldwide, causing many casualties in the human population. This devastating event taught us a fundamental lesson on how emerging and re‐emerging pathogenic viruses can have catastrophic consequences. For these reasons, it is imperative to find effective antiviral agents as fast as possible. In this context, the SARS‐CoV‐2 nsp13 is a pivotal enzyme in the viral life cycle and shows a high interspecific similarity among coronaviruses. Pursuing our studies on the development of nsp13 inhibitors, we reported the synthesis of a new series of indolyl DKA derivatives structurally related to the ones we recently described, to deepen the SAR studies within this class of compounds. Among them, dikehexenoic derivatives were proven as the most promising, being good dual inhibitors of both the nsp13‐associated activities with highly promising inhibitory potencies against both the unwinding and ATPase activities. In contrast, diketobutanoic derivatives reported less encouraging inhibitory values. Importantly, in cellulo assays showed their ability to block viral replication in SARS‐CoV‐2 infected cells, without exerting cytotoxicity. Moreover, docking studies helped us to rationalize the structural features involved in the interaction within the allosteric pocket located in the Rec2A domain, providing useful insights for the future development of new SARS‐CoV‐2 nsp13 inhibitors.

## Conflict of Interest

The authors declare no conflict of interest.

## Author Contributions

**Valentina Noemi Madia**: formal analysis (equal); investigation (equal); writing—original draft (equal); **Roberta Emmolo**: software (lead); writing—original draft (equal); **Elisa Patacchini**: investigation (equal); writing—original draft (equal); **Donatella Amatore**: investigation (equal); **Stefania Maloccu**: visualization (equal); **Davide Ialongo**: investigation (equal); **Aurora Albano**: formal analysis (equal); **Giuseppe Ruggieri**: data curation (equal); visualization (equal); **Emanuele Cara**: data curation (equal); visualization (equal); **Laura Zarbo**: validation (supporting); **Antonella Messore**: methodology (equal); writing—original draft (equal); writing—review and editing (equal); **Riccardo De Santis**: formal analysis (equal); investigation (equal): methodology (equal); **Alessandra Amoroso**: data curation (equal); project administration (equal); resources (equal); supervision (equal); **Florigio Lista**: funding acquisition (equal); supervision (equal); **Francesca Esposito**: resources (equal); supervision (equal); visualization (equal); **Enzo Tramontano**: data curation (equal); validation (lead); visualization (lead); writing—review and editing (equal); **Angela Corona**: formal analysis (lead); funding acquisition (lead); project administration (lead); resources (lead); visualization (equal); **Roberto Di Santo**: conceptualization (lead); data curation (lead); funding acquisition (lead); methodology (lead); supervision (lead); writing—review and editing (equal); **Roberta Costi**: Conceptualization (lead); data curation (lead); funding acquisition (lead); methodology (lead); supervision (lead); writing—review and editing (equal).

## Supporting information

Supplementary Material

## Data Availability

The data that support the findings of this study are available from the corresponding author upon reasonable request.

## References

[1] B. A. Williams , C. H. Jones , V. Welch , J. M. True , NPJ Vaccines 2023, 8, 178. 37985781 10.1038/s41541-023-00773-0 PMC10662147

[2] “World Health Organization . COVID‐19 epidemiological update – 24 December 2024”, can be found under https://www.who.int/publications/m/item/covid‐19‐epidemiological‐update‐‐‐24‐december‐2024 **2024** (accessed: 07 January 2025).

[3] A. Tuekprakhon , R. Nutalai , A. Dijokaite‐Guraliuc , D. Zhou , H. M. Ginn , M. Selvaraj , C. Liu , A. J. Mentzer , P. Supasa , H. M. E. Duyvesteyn , R. Das , D. Skelly , T. G. Ritter , A. Amini , S. Bibi , S. Adele , S. A. Johnson , B. Constantinides , H. Webster , N. Temperton , P. Klenerman , E. Barnes , S. J. Dunachie , D. Crook , A. J. Pollard , T. Lambe , P. Goulder , N. G. Paterson , M. A. Williams , D. R. Hall , E. E. Fry , J. Huo , J. Mongkolsapaya , J. Ren , D. I. Stuart , G. R. Screaton , Cell 2022, 185, 2422. 35772405 10.1016/j.cell.2022.06.005 PMC9181312

[4] S. Iketani , L. Liu , Y. Guo , J. F. Chan , Y. Huang , M. Wang , Y. Luo , J. Yu , H. Chu , K. K. Chik , T. T. Yuen , M. T. Yin , M. E. Sobieszczyk , K. Y. Yuen , H. H. Wang , Z. Sheng , D. D. Ho , Nature 2022, 604, 553. 35240676 10.1038/s41586-022-04594-4 PMC9021018

[5] “U.S. Food and Drug Administration. FDA Coronavirus (COVID‐19) Drugs .” can be found under https://www.fda.gov/drugs/emergency‐preparedness‐drugs/coronavirus‐covid‐19‐drugs 2025 (accessed: 07 January 2025).

[6] M. Wang , R. Cao , L. Zhang , X. Yang , J. Liu , M. Xu , Z. Shi , Z. Hu , W. Zhong , G. Xiao , Cell Res. 2020, 30, 269. 32020029 10.1038/s41422-020-0282-0 PMC7054408

[7] Y. Xie , W. Yin , Y. Zhang , W. Shang , Z. Wang , X. Luan , G. Tian , H. A. Aisa , Y. Xu , G. Xiao , J. Li , H. Jiang , S. Zhang , L. Zhang , H. E. Xu , J. Shen , Cell Res. 2021, 31, 1212. 34584244 10.1038/s41422-021-00570-1 PMC8477624

[8] Y. Y. Syed , Drugs 2022, 82, 455. 35184266 10.1007/s40265-022-01684-5 PMC8858220

[9] “European Medicines Agency – Lagevrio ‐ Application withdrawn.” can be found under https://www.ema.europa.eu/en/medicines/human/EPAR/lagevrio 2025 (accessed: 07 January 2025).

[10] D. R. Owen , C. M. N. Allerton , A. S. Anderson , L. Aschenbrenner , M. Avery , S. Berritt , B. Boras , R. D. Cardin , A. Carlo , K. J. Coffman , A. Dantonio , L. Di , H. Eng , R. Ferre , K. S. Gajiwala , S. A. Gibson , S. E. Greasley , B. L. Hurst , E. P. Kadar , A. S. Kalgutkar , J. C. Lee , J. Lee , W. Liu , S. W. Mason , S. Noell , J. J. Novak , R. S. Obach , K. Ogilvie , N. C. Patel , M. Pettersson , D. K. Rai , M. R. Reese , M. F. Sammons , J. G. Sathish , R. S. P. Singh , C. M. Steppan , A. E. Stewart , J. B. Tuttle , L. Updyke , P. R. Verhoest , L. Wei , Q. Yang , Y. Zhu , Science 2021, 374, 1586. 34726479 10.1126/science.abl4784

[11] Y. Unoh , S. Uehara , K. Nakahara , H. Nobori , Y. Yamatsu , S. Yamamoto , Y. Maruyama , Y. Taoda , K. Kasamatsu , T. Suto , K. Kouki , A. Nakahashi , S. Kawashima , T. Sanaki , S. Toba , K. Uemura , T. Mizutare , S. Ando , M. Sasaki , Y. Orba , H. Sawa , A. Sato , T. Sato , T. Kato , Y. Tachibana , J. Med. Chem. 2022, 65, 6499. 35352927 10.1021/acs.jmedchem.2c00117 PMC8982737

[12] “Ministry of Health , Labour and Welfare of Japan (MHW): about urgent approval of new‐style coronavirus medicine. Emergency Approval of Drug for Treating COVID‐19.” can be found under https://www.mhlw.go.jp/stf/newpage_29320.html **2020** (accessed: 07 January 2025).

[13] “Shionogi ‐ Shionogi Receives U.S. FDA Fast Track Designation for Ensitrelvir Fumaric Acid, an Investigational Oral Antiviral for COVID‐19.” can be found under https://www.shionogi.com/global/en/news/2023/04/20230404.html **2023** (accessed: 07 January 2025).

[14] H. Yotsuyanagi , N. Ohmagari , Y. Doi , M. Yamato , N. H. Bac , B. K. Cha , T. Imamura , T. Sonoyama , G. Ichihashi , T. Sanaki , Y. Tsuge , T. Uehara , H. Mukae , JAMA Netw. Open 2024, 7, e2354991. 38335000 10.1001/jamanetworkopen.2023.54991 PMC10858401

[15] S. Iketani , H. Mohri , B. Culbertson , S. J. Hong , Y. Duan , M. I. Luck , M. K. Annavajhala , Y. Guo , Z. Sheng , A. C. Uhlemann , S. P. Goff , Y. Sabo , H. Yang , A. Chavez , D. D. Ho , Nature 2023, 613, 558. 36351451 10.1038/s41586-022-05514-2 PMC9849135

[16] D. Jochmans , C. Liu , K. Donckers , A. Stoycheva , S. Boland , S. K. Stevens , C. De Vita , B. Vanmechelen , P. Maes , B. Trüeb , N. Ebert , V. Thiel , S. De Jonghe , L. Vangeel , D. Bardiot , A. Jekle , L. M. Blatt , L. Beigelman , J. A. Symons , P. Raboisson , P. Chaltin , A. Marchand , J. Neyts , J. Deval , K. Vandyck , mBio 2023, 14, e0281522. 36625640 10.1128/mbio.02815-22 PMC9973015

[17] G. D. Noske , E. de Souza Silva , M. O. de Godoy , I. Dolci , R. S. Fernandes , R. V. C. Guido , P. Sjö , G. Oliva , A. S. Godoy , J. Biol. Chem. 2023, 299, 103004. 36775130 10.1016/j.jbc.2023.103004 PMC9916189

[18] F. R. Brennan , L. D. Morton , S. Spindeldreher , A. Kiessling , R. Allenspach , A. Hey , P. Y. Muller , W. Frings , J. Sims , mAbs 2010, 2, 233. 20421713 10.4161/mabs.2.3.11782 PMC2881251

[19] “Emergency Use Authorization ‐ Archived Information ‐Terminated or Revoked EUAs .” can be found under https://www.fda.gov/emergency‐preparedness‐and‐response/mcm‐legal‐regulatory‐and‐policy‐framework/emergency‐use‐authorization‐archived‐information **2025** (accessed: 07 January 2025).

[20] M. Amicone , V. Borges , M. J. Alves , J. Isidro , L. Zé‐Zé , S. Duarte , L. Vieira , R. Guiomar , J. P. Gomes , I. Gordo , Evol. Med. Public Health. 2022, 10, 142. 35419205 10.1093/emph/eoac010 PMC8996265

[21] N. Mehyar , J. Virus Erad. 2023, 9, 100327. 37363132 10.1016/j.jve.2023.100327 PMC10214743

[22] M. A. White , W. Lin , X. Cheng , J. Phys. Chem. Lett. 2020, 11, 9144. 33052685 10.1021/acs.jpclett.0c02421 PMC7571306

[23] A. Wu , Y. Peng , B. Huang , X. Ding , X. Wang , P. Niu , J. Meng , Z. Zhu , Z. Zhang , J. Wang , J. Sheng , L. Quan , Z. Xia , W. Tan , G. Cheng , T. Jiang , Cell Host Microbe 2020, 27, 325. 32035028 10.1016/j.chom.2020.02.001 PMC7154514

[24] A. O. Adedeji , B. Marchand , A. J. Te Velthuis , E. J. Snijder , S. Weiss , R. L. Eoff , K. Singh , S. G. Sarafianos , PloS One 2012, 7, e36521. 22615777 10.1371/journal.pone.0036521 PMC3352918

[25] K. Saikrishnan , B. Powell , N. J. Cook , M. R. Webb , D. B. Wigley , Cell 2009, 137, 849. 19490894 10.1016/j.cell.2009.03.036

[26] J. Chen , B. Malone , E. Llewellyn , M. Grasso , P. M. M. Shelton , P. D. B. Olinares , K. Maruthi , E. T. Eng , H. Vatandaslar , B. T. Chait , T. M. Kapoor , S. A. Darst , E. A. Campbell , Cell 2020, 182, 1560. 32783916 10.1016/j.cell.2020.07.033 PMC7386476

[27] Z. Jia , L. Yan , Z. Ren , L. Wu , J. Wang , J. Guo , L. Zheng , Z. Ming , L. Zhang , Z. Lou , Z. Rao , Nucleic Acids Res. 2019, 47, 6538. 31131400 10.1093/nar/gkz409 PMC6614802

[28] P. V’Kovski , A. Kratzel , S. Steiner , H. Stalder , V. Thiel , Nat. Rev. Microbiol. 2021, 19, 155. 33116300 10.1038/s41579-020-00468-6 PMC7592455

[29] A. Corona , K. Wycisk , C. Talarico , C. Manelfi , J. Milia , R. Cannalire , F. Esposito , P. Gribbon , A. Zaliani , D. Iaconis , A. R. Beccari , V. Summa , M. Nowotny , E. Tramontano , ACS Pharmacol. Transl. Sci. 2022, 5, 226. 35434533 10.1021/acsptsci.1c00253 PMC9003574

[30] M. G. Nizi , L. Persoons , A. Corona , T. Felicetti , G. Cernicchi , S. Massari , G. Manfroni , L. Vangeel , M. L. Barreca , F. Esposito , D. Jochmans , J. Milia , V. Cecchetti , D. Schols , J. Neyts , E. Tramontano , S. Sabatini , S. De Jonghe , O. Tabarrini , ACS Med. Chem. Lett. 2022, 13, 855. 35571875 10.1021/acsmedchemlett.2c00123 PMC9088073

[31] J. Zeng , F. Weissmann , A. P. Bertolin , V. Posse , B. Canal , R. Ulferts , M. Wu , R. Harvey , S. Hussain , J. C. Milligan , C. Roustan , A. Borg , L. McCoy , L. S. Drury , S. Kjaer , J. McCauley , M. Howell , R. Beale , J. F. X. Diffley , Biochem. J. 2021, 478, 2405. 34198322 10.1042/BCJ20210201 PMC8286831

[32] L. Lu , Y. Peng , H. Yao , Y. Wang , J. Li , Y. Yang , Z. Lin , Antiviral Res. 2022, 206, 105389. 35985407 10.1016/j.antiviral.2022.105389 PMC9381947

[33] A. Corona , V. N. Madia , R. De Santis , C. Manelfi , R. Emmolo , D. Ialongo , E. Patacchini , A. Messore , D. Amatore , G. Faggioni , M. Artico , D. Iaconis , C. Talarico , R. Di Santo , F. Lista , R. Costi , E. Tramontano , Antiviral Res. 2023, 217, 105697. 37562607 10.1016/j.antiviral.2023.105697

[34] R. Costi , G. C. Crucitti , L. Pescatori , A. Messore , L. Scipione , S. Tortorella , A. Amoroso , E. Crespan , P. Campiglia , B. Maresca , A. Porta , I. Granata , E. Novellino , J. Gouge , M. Delarue , G. Maga , R. Di Santo , J. Med. Chem. 2013, 56, 7431. 23968551 10.1021/jm4010187

[35] R. Di Santo , R. Costi , M. Artico , G. Miele , A. Lavecchia , E. Novellino , A. Bergamini , R. Cancio , G. Maga , ChemMedChem 2006, 1, 1367. 17089433 10.1002/cmdc.200600119

[36] E. Mons , S. Roet , R. Q. Kim , M. P. C. Mulder , Curr. Protoc. 2022, 2, e419. 35671150 10.1002/cpz1.419

