# Supplementary material for: Structure−Activity Relationships of New 1‐Aryl‐1H‐Indole Derivatives as SARS‐CoV‐2 Nsp13 Inhibitors
Source: ChemMedChem. 2025 May 20;20(14):e202500205. doi: 10.1002/cmdc.202500205 (PMC12276029; doi:10.1002/cmdc.202500205)
Supplement: Supplementary file 1 — Supplementary Material [file CMDC-20-e202500205-s001.pdf]

Supporting Information

Structure−Activity Relationships of New 1-Aryl-1*H*-indole Derivatives as SARS-CoV-2 Nsp13 Inhibitors

Valentina Noemi Madia,[a] Roberta Emmolo,[b] Elisa Patacchini,[a] Donatella Amatore,[c] Stefania Maloccu,[b] Davide Ialongo,[a] Aurora Albano,[a] Giuseppe Ruggieri,[d,a] Emanuele Cara,[a] Laura Zarbo,[a] Antonella Messore,[a,e] Riccardo De Santis,[c,f] Alessandra Amoroso,[c] Florigio Lista,[c] Francesca Esposito,[b] Enzo Tramontano,[b] Angela Corona,[b] Roberto Di Santo,*[a] and Roberta Costi [a]

[a] Dr. Valentina Noemi Madia, Dr. Elisa Patacchini, Dr. Davide Ialongo, Dr. Aurora Albano, Dr. Emanuele Cara, Dr. Laura Zarbo, Prof. Roberto Di Santo, Prof. Roberta Costi
Dipartimento di Chimica e Tecnologie del Farmaco, Istituto Pasteur-Fondazione Cenci Bolognetti
"Sapienza" Università di Roma
p.le Aldo Moro 5, I-00185, Rome, Italy
E-mail: roberto.disanto@uniroma1.it

[b] Dr. Roberta Emmolo, Dr. Stefania Maloccu, Prof. Francesca Esposito, Prof. Enzo Tramontano, Prof. Angela Corona
Dipartimento di Scienze della Vita e dell'Ambiente Sezione biomedica, Laboratorio di Virologia Molecolare Blocco E, primo piano
Università di Cagliari
Cittadella Universitaria di Monserrato, SS554 -09042 Monserrato (CA) Italia

[c] Dr. Donatella Amatore, Dr. Alessandra Amoroso, Dr. Florigio Lista
Istituto di Scienze Biomediche della Difesa
00184 Roma, Italy

[d] Dr. Giuseppe Ruggieri
Dottorato di Interesse Nazionale in One Health approaches to infectious diseases and life science research
Dipartimento di Sanità Pubblica, Medicina Sperimentale e Forense, Università degli Studi di Pavia
Pavia, 27100, Italia

[e] Dr. Antonella Messore
Department of Life Science, Health, and Health Professions
Link Campus University
Via del Casale di San Pio V 44, I-00165, Rome, Italy

[f] Dr. Riccardo De Santis
Dipartimento di Sanità Pubblica e Malattie Infettive
"Sapienza" Università di Roma
00161 Roma, Italy

Contents

Chemistry Experimental Section	S2-S12
General Instrumentation	S2
General Experimental Procedures	S2-S4
Specific Procedures and Characterization	S4-S12
Biology Experimental Section	S12-S14
Molecular Modeling Experimental Section	S14-S15
References	S15-S16
Figure S1 – S60: FTIR, ^{1}H NMR, ^{13}C NMR Spectra for compounds **5a−c,g,h, 6a−c,g,h, 7a−e and 8a−e.**	S17-S77
Figure S61	S78
Figure S62	S79

Chemistry Experimental section

General Instrumentation. Melting points were determined on a Bobby Stuart Scientific SMP1 melting point apparatus and are uncorrected. Compound purity was always >95% as determined by combustion analysis. Analytical results agreed to within ± 0.40% of the theoretical values. Infrared (IR) spectra were recorded on a PerkinElmer Spectrum-One spectrophotometer. ^{1}H NMR and ^{13}C NMR spectra were recorded at 400 MHz and 100 MHz, respectively, on a Bruker AC 400 Ultrashield 10 spectrometer (400 MHz); the following abbreviations were used: s for singlet, bs for broad singlet, d for doublet, t for triplet, dd for double doublet, m for multiplet; chemical shift are given in d with respect to the residual solvent signal, coupling constant are given in Hz. Dimethyl sulfoxide-d_6 99.9% (CAS 2206-27-1) and deuteromethanol-d_4 98.8% (CAS 811-98-3) of isotopic purity (Aldrich) were used. Column chromatography was performed on silica gel (Merck, 70−230 mesh) or alumina (Merck, 70−230 mesh). All compounds were routinely checked on TLC by using aluminium-baked silica gel plates (Merck silica-gel 60 F_{254} plates). Developed plates were visualized by UV light. Solvents and reagent were of analytical-grade and, when necessary, were purified and dried by standard methods. Concentration of solutions after reactions and extractions involved the use of rotary evaporator (Büchi) operating at a reduced pressure (ca. 20 Torr). Organic solutions were dried over anhydrous sodium sulfate (Merck). All solvents were freshly distilled under nitrogen and stored over molecular sieves for at least 3 h prior to use.

General Experimental Procedures

General Procedure A (GP-A) to Obtain *N*-phenyl Indoles (9a–c,g,h and **11a–e)**. To a solution of 1*H*-indole-3-carboxaldehyde (for compounds **9a–c,g,h**) or 3-acetylindole (for compounds **11a–e**) (24.0 mmol) in anhydrous DMF (30 mL) were added Cu_2O (7.2 mmol), K_2CO_3 (48.0 mmol), and the proper iodobenzene derivative (48.0 mmol) sequentially. The reaction was stirred under reflux for 24 hours and monitored by TLC. After completion, the mixture was cooled to room temperature and filtrated through a Celite pad eluting with ethyl acetate.[1] The organic layer was washed with brine (3 x 300 mL), dried over Na_2SO_4, concentrated, and purified by column chromatography on silica gel. For each compound proper iodobenzene derivative; chromatography eluent; recrystallization solvent; yield (%); melting point (°C); IR; ^{1}H NMR; ^{13}C NMR and elemental analysis are reported.

General procedure B (GP-B) to Obtain a,b-Unsaturated Ketones (10a–c,g,h). The proper indole carboxaldehyde (8.5 mmol) was dissolved in 29 mL of acetone. To this mixture was added

5 N NaOH (12.4 mL), and the mixture was stirred at 50 °C for 24 h. After this period water (300 mL) was added and the solid that formed was filtrated under vacuum, washed with water and light petroleum ether to obtain pure compounds. For compound **10a,** after completion of the reaction water (300 mL) and ethyl acetate (250 mL) were added. The organic layer was separated, washed with water (2 × 100 mL), dried over Na_2SO_4, filtered, and evaporated under reduced pressure. For compounds **10a,c,h,** the crude product was purified by column chromatography on silica gel to obtain pure products eluting with chloroform /ethyl acetate 5:5 (for **10a**) or *n*-hexane/ethyl acetate 7:3 (for **10c,h**). For compounds **10b,g,** after completion of the reaction, water was added and the solid that formed was filtered on gooch, then washed with petroleum ether and dried under IR lamp to obtain **10g** as pure compound. For each compound proper indole carboxaldehyde; chromatography eluent; recrystallization solvent; yield (%); melting point (°C); IR; ^{1}H NMR; ^{13}C NMR and elemental analysis are reported.

General procedure C (GP-C) to Obtain Diketo Esters (6a–c,g,h and **8a–e)**. For compounds **6a–c,g,h**, freshly prepared sodium ethoxide (7.2 mmol), obtained by the dissolution of Na (72 mmol) in 7.47 mL of absolute ethanol, was added to a well-stirred solution of the proper a,b-unsaturated ketone derivative (18 mmol) and diethyl oxalate (72 mmol) in anhydrous THF (4 mL) under argon atmosphere.

For compounds **8a–e**, freshly prepared sodium ethoxide (34 mmol), obtained as above, was added to a well-stirred solution of the proper acetyl derivative (17 mmol) in anhydrous THF (3.8 mL) under argon atmosphere; afterwards, diethyl oxalate (34 mmol) was added.

The mixture was stirred at room temperature for the proper time under argon atmosphere and then was poured into *n*-hexane (200 mL). The resulting precipitate was vigorously stirred for 30 min in 1 N HCl (200 mL). The solid that formed was filtered, washed with water and light petroleum ether, and dried under IR lamp to afford the pure diketo esters. For each compound proper aldehyde or acetyl derivative; reaction time; recrystallization solvent; yield (%); melting point (°C); IR; ^{1}H NMR; ^{13}C NMR and elemental analysis are reported.

General procedure D (GP-D) to Obtain Diketo Acids (5a–c,g,h and **7a–e)**. A solution of 1 N NaOH (11.9 mmol) was added to a solution of the appropriate ester (2.39 mmol) in 1:1 THF/methanol (54 mL) and the reaction was stirred vigorously at room temperature for the proper time. The organic phase was removed under vacuum and to the resulting suspension crushed ice was added. The mixture was acidified with 1N HCl up to pH 4-5 and the solid that formed was filtered, washed with water and light petroleum ether, and dried under IR lamp (or extracted with ethyl acetate, dried over Na_2SO_4, filtered and evaporated under reduced pressure for derivatives

5a and **7c–e**) to afford pure acids. For each compound appropriate ester; reaction time; recrystallization solvent; yield (%); melting point (°C); IR; ^{1}H NMR; ^{13}C NMR and elemental analysis are reported.

*(2Z,5E)-2-hydroxy-6-(1-(4-methoxyphenyl)-1H-indol-3-yl)-4-oxohexa-2,5-dienoic acid (**5a**)*. Compound **5a** was prepared from **6a** by means of GP-D; 45 min; washed with ethanol; 31% as a red solid; decomposes at 110 °C; IR ν OH 2934, CO acid 1726, CO ketone 1602 cm^{-1}; ^{1}H NMR (400 MHz, DMSO-d_6) δ 8.30 (s, 1H, indole C2-H), 8.23 – 8.20 (m, 1H, indole C4-H), 8.08 (d, J = 15.9 Hz, 1H, hexenoate C6-H), 7.56 (d, J = 8.9 Hz, 2H, benzene H), 7.48 – 7.45 (m, 1H, indole C7-H), 7.36 – 7.31 (m, 2H, indole C5-H and C6-H), 7.17 (d, J = 8.9 Hz, 2H, benzene H), 7.08 (d, J = 15.9 Hz, 1H, hexenoate C5-H), 6.70 (s, 1H, CH_2), 3.85 (s, 3H, CH_3). ^{13}C NMR (101 MHz, DMSO-d_6) δ 187.56, 171.63, 163.55, 158.69, 137.51, 137.42, 136.14, 130.62, 126.04, 125.76, 123.87, 122.29, 121.10, 118.67, 115.07, 113.75, 111.30, 101.12, 55.56. Anal. calcd for $C_{21}H_{17}NO_5$: C, 69.41; H, 4.72; N, 3.85%. Found C, 69.35; H, 4.73; N, 3.84%.

*(2Z,5E)-2-hydroxy-4-oxo-6-(1-(p-tolyl)-1H-indol-3-yl)hexa-2,5-dienoic acid (**5b**)*. Compound **5b** was prepared from **6b** by means of GP-D; 30 min; washed with isopropanol; 75% as a red solid; 120 - 122 °C; IR ν OH 2918, CO acid 1720, CO ketone 1591 cm^{-1}; ^{1}H NMR (400 MHz, DMSO-d_6) δ 13.85 (bs, 1H, OH), 8.33 (s, 1H, indole C2-H), 8.23 – 8.20 (m, 1H, indole C4-H), 8.07 (d, J = 16.0 Hz, 1H, hexenoate C6-H), 7.54 – 7.50 (m, 3H, benzene H and indole C7-H), 7.43 (d, J = 8.0 Hz, 2H, benzene H), 7.34 – 7.32 (m, 2H, indole C5-H and C6-H), 7.05 (d, J = 16.0 Hz, 1H, hexenoate C5-H), 6.69 (s, 1H, hexenoate C3-H), 2.42 (s, 3H, CH_3). ^{13}C NMR (101 MHz, CD_3OD-d_4) δ 196.72, 180.31, 171.48, 171.29, 147.12, 146.88, 146.67, 145.56, 144.76, 139.89, 135.40, 133.78, 133.47, 131.90, 130.67, 128.01, 123.55, 120.88, 110.71, 30.13. Anal. calcd for $C_{21}H_{17}NO_4$: C, 72.61; H, 4.93; N, 4.03%. Found: C, 72.69; H, 4.92; N, 4.04%.

*(2Z,5E)-2-hydroxy-6-(1-(4-isopropylphenyl)-1H-indol-3-yl)-4-oxohexa-2,5-dienoic acid (**5c**)*. Compound **5c** was prepared from **6c** by means of GP-D; 15 min; isopropanol; 42% as a red solid; 165 - 168 °C; IR ν OH 3262, CO acid 1618, CO ketone 1590 cm^{-1}; ^{1}H NMR (400 MHz, DMSO-d_6) δ 13.79 (bs, 1H, OH), 8.34 (s, 1H, indole C2-H), 8.24 – 8.21 (m, 1H, indole C4-H), 8.08 (d, J = 15.9 Hz, 1H, hexenoate C6-H), 7.58 – 7.49 (m, 5H, benzene H and indole C7-H), 7.34 – 7.32 (m, 2H, indole C5-H and C6-H), 7.05 (d, J = 15.9 Hz, 1H, hexenoate C5-H), 6.70 (s, 1H, hexenoate C3-H), 3.01 (q, J = 7.0 Hz, 1H, CH), 1.28 (d, J = 6.8 Hz, 6H, CH_3). ^{13}C NMR (101 MHz, DMSO-d_6) δ 187.64, 170.19, 162.07, 147.94, 138.32, 137.51, 137.27, 134.38, 127.36, 126.60, 125.55, 123.25, 121.83, 120.98, 117.28, 112.61, 111.51, 101.13, 61.83, 49.36, 33.09, 23.79, 13.94. Anal. calcd for $C_{23}H_{21}NO_4$: C, 73.58; H, 5.64; N, 3.73%. Found: C, 73.50; H, 5.65; N, 3.72%.

*(2Z,5E)-2-hydroxy-4-oxo-6-(1-phenyl-1H-indol-3-yl)hexa-2,5-dienoic acid (**5d**)*. Synthesis, analytical, and spectroscopic data are reported in the literature.[2]

*(2Z,5E)-6-(1-(4-chlorophenyl)-1H-indol-3-yl)-2-hydroxy-4-oxohexa-2,5-dienoic acid (**5f**)*. Synthesis, analytical, and spectroscopic data are reported in the literature.[2]

*(2Z,5E)-6-(1-(4-cyanophenyl)-1H-indol-3-yl)-2-hydroxy-4-oxohexa-2,5-dienoic acid (**5g**)*. Compound **5g** was prepared from **6g** by means of GP-D; 1 h; DMF/H_2O; 90% as a red solid; 190 - 192 °C; IR ν OH 3107, CO acid 1722, CO ketone 1509 cm^{-1}; ^1H NMR (400 MHz, DMSO-d_6) δ 8.39 (s, 1H, indole C2-H), 8.20 – 8.09 (m, 4H, indole C4-H, hexenoate C6-H and benzene H), 7.91 – 7.69 (m, 4H, indole C7-H, benzene H and hexenoate C5-H), 7.50 – 7.36 (m, 2H, indole C5-H and C6-H), 7.05 (s, 1H, hexenoate C3-H). ^{13}C NMR (101 MHz, CD_3OD-d_4) δ 196.24, 180.82, 171.35, 170.40, 151.03, 145.87, 144.58, 143.70, 135.89, 134.15, 133.93, 132.43, 130.80, 129.15, 127.83, 124.79, 120.98, 119.13, 110.72. Anal. calcd for $C_{21}H_{14}N_2O_4$: C, 70.39; H, 3.94; N, 7.82%. Found: C, 70.45; H, 3.95; N, 7.81%.

*(2Z,5E)-2-hydroxy-4-oxo-6-(1-(4-(trifluoromethyl)phenyl)-1H-indol-3-yl)hexa-2,5-dienoic acid (**5h**)*. Compound **5h** was prepared from **6h** by means of GP-D; 15 min; DMF/H_2O; 22% as a red solid; 185 - 187 °C; IR ν OH 3106, CO acid 1591, CO ketone 1524 cm^{-1}; ^1H NMR (400 MHz, DMSO-d_6) δ 8.47 (s, 1H, indole C2-H), 8.27 – 8.25 (m, 1H, indole C4-H), 8.07 (d, *J* = 15.9 Hz, 1H, hexenoate C6-H), 8.01 (d, *J* = 8.4 Hz, 2H, benzene H), 7.92 (d, *J* = 8.3 Hz, 2H, benzene H), 7.70 – 7.68 (m, 1H, indole C7-H), 7.41 – 7.35 (m, 2H, indole C5-H and C6-H), 7.11 (d, *J* = 16.0 Hz, 1H, hexenoate C5-H), 6.73 (s, 1H, hexenoate C3-H). ^{13}C NMR (101 MHz, DMSO-d_6) δ 187.21, 172.17, 163.47, 141.21, 136.58, 135.11, 127.22, 127.18, 126.35, 125.37, 124.77, 124.35, 122.81, 121.29, 119.82, 115.02, 111.42, 101.17. Anal. calcd for $C_{21}H_{14}F_3NO_4$: C, 62.85; H, 3.52; F, 14.20; N, 3.49 %. Found: C, 62.90; H, 3.53; F, 14.18; N, 3.48%.

*Ethyl (2Z,5E)-2-hydroxy-6-(1-(4-methoxyphenyl)-1H-indol-3-yl)-4-oxohexa-2,5-dienoate (**6a**)*. Compound **6a** was prepared from **10a** by means of GP-C; 20 min; isopropanol; 100% as a red solid; 118- 120 °C; IR ν CO ester 1738, CO ketone 1624 cm^{-1};^1H NMR (400 MHz, DMSO-d_6) δ 8.32 (s, 1H, indole C2-H), 8.25 – 8.22 (m, 1H, indole C4-H), 8.10 (d, *J* = 15.9 Hz, 1H, hexenoate C6-H), 7.56 (d, *J* = 8.9 Hz, 2H, benzene H), 7.48 – 7.46 (m, 1H, indole C7-H), 7.34 – 7.32 (m, 2H, indole C5-H and C6-H), 7.17 (d, 2H, *J* = 8.9 Hz, benzene H), 7.07 (d, *J* = 15.9 Hz, 1H, hexenoate C5-H), 6.73 (s, 1H, hexenoate C3-H), 4.30 (q, *J* = 7.1 Hz, 2H, *CH_2*CH_3), 3.85 (s, 3H, CH_3), 1.31 (t, *J* = 7.1 Hz, 3H, CH_2*CH_3*). ^{13}C NMR (101 MHz, DMSO-d_6) δ 187.27, 170.75, 161.99, 158.70, 137.74, 137.54, 136.39, 130.58, 126.03, 125.73, 123.91, 122.33, 121.15, 118.32, 115.06, 113.80,

111.32, 101.21, 61.88, 55.56, 13.94. Anal. calcd for $C_{23}H_{21}NO_5$: C, 70.58; H, 5.41; N, 3.58%. Found: C, 70.50; H, 5.42; N, 3.59%.

*Ethyl (2Z,5E)-2-hydroxy-4-oxo-6-(1-(p-tolyl)-1H-indol-3-yl)hexa-2,5-dienoate (**6b**)*. Compound **6b** was prepared from **10b** by means of GP-C; 20 min; isopropanol; 98% as a red solid; 110 - 112 °C; IR ν OH 2979, CO ester 1741, CO ketone 1514 cm^{-1}; ^1H NMR (400 MHz, DMSO-d_6) δ 8.35 (s, 1H, indole C2-H), 8.25 – 8.23 (m, 1H, indole C4-H), 8.10 (d, J = 15.8 Hz, 1H, hexenoate C6-H), 7.55 – 7.51 (m, 3H, benzene H and indole C7-H), 7.43 (d, J = 8.0 Hz, 2H, benzene H), 7.34 (m, 2H, indole C5-H and C6-H), 7.08 (d, J = 15.8 Hz, 1H, hexenoate C5-H), 6.73 (s, 1H, hexenoate C3-H), 4.30 (q, J = 7.1 Hz, 2H, $CH_2$$CH_3$), 2.42 (s, 3H, CH_3), 1.31 (t, J = 7.0 Hz, 3H, $CH_2$$CH_3$). ^{13}C NMR (101 MHz, DMSO-d_6) δ 187.24, 170.83, 162.00, 137.64, 137.40, 137.19, 136.08, 135.28, 130.41, 125.92, 124.30, 123.99, 122.42, 121.19, 118.53, 114.07, 111.40, 101.23, 61.89, 20.65, 13.95. Anal. calcd for $C_{23}H_{21}NO_4$: C, 73.58; H, 5.64; N, 3.73%. Found: C, 73.40; H, 5.63; N, 3.74%.

*Ethyl (2Z,5E)-2-hydroxy-6-(1-(4-isopropylphenyl)-1H-indol-3-yl)-4-oxohexa-2,5-dienoate (**6c**)*. Compound **6c** was prepared from **10c** by means of GP-C; 20 min; washed with diisopropyl ether; 61% as a red solid; 97 - 100 °C; IR ν OH 3409, CO ester 1730, CO ketone 1514 cm^{-1}; ^1H NMR (400 MHz, DMSO-d_6) δ 8.36 (s, 1H, indole C2-H), 8.25 – 8.23 (m, 1H, indole C4-H), 8.09 (d, J = 15.9 Hz, 1H, hexenoate C6-H), 7.57 – 7.49 (m, 5H, benzene H and indole C7-H), 7.35 – 7.33 (m, 2H, indole C5-H and C6-H), 7.08 (d, J = 15.8 Hz, 1H, hexenoate C5-H), 6.73 (s, 1H, hexenoate C3-H), 4.30 (q, J = 7.0 Hz, 2H $CH_2$$CH_3$), 3.05 – 3.00 (m, 1H, CH), 1.33 – 1.27 (m, 9H, $CH_2$$CH_3$ and CH_3). ^{13}C NMR (101 MHz, DMSO-d_6) δ 187.20, 170.84, 161.97, 148.15, 137.62, 137.16, 136.13, 135.53, 127.80, 125.90, 124.35, 123.99, 122.41, 121.20, 118.50, 114.07, 111.43, 101.23, 61.88, 33.12, 23.82, 13.94. Anal. calcd for $C_{25}H_{25}NO_4$: C, 74.42; H, 6.25; N, 3.47%. Found: C, 74.55; H, 6.24; N, 3.46%.

*Ethyl (2Z,5E)-2-hydroxy-4-oxo-6-(1-phenyl-1H-indol-3-yl)hexa-2,5-dienoate (**6d**)*. Synthesis, analytical, and spectroscopic data are reported in the literature.[2]

*Ethyl (2Z,5E)-6-(1-(4-chlorophenyl)-1H-indol-3-yl)-2-hydroxy-4-oxohexa-2,5-dienoate (**6f**)*. Synthesis, analytical, and spectroscopic data are reported in the literature.[2]

*Ethyl (2Z,5E)-6-(1-(4-cyanophenyl)-1H-indol-3-yl)-2-hydroxy-4-oxohexa-2,5-dienoate (**6g**)*. Compound **6g** was prepared from **10g** by means of GP-C; 1.5 h; toluene; 91% as an orange solid; 182 - 185 °C; IR ν OH 3385, CN 2224, CO ester 1737, CO ketone 1622 cm^{-1}; ^1H NMR (400 MHz, DMSO-d_6) δ 8.53 (s, 1H, indole C2-H), 8.33 – 8.31 (m, 1H, indole C4-H), 8.17 – 8.10 (m, 3H,

hexenoate C6-H and benzene H), 7.95 (d, J = 6.5 Hz, 2H, benzene H) 7.75 – 7.73 (m, 1H, indole C7-H), 7.46 – 7.40 (m, 2H, indole C5-H and C6-H), 7.19 (d, *J* = 16.0 Hz, 1H, hexenoate C5-H), 6.79 (s, 1H, hexenoate C3-H), 4.34 (q, *J* = 7.4 Hz, 2H CH_2CH_3), 1.35 (t, J = 7.1 Hz, 3H, CH_2CH_3). ^{13}C NMR (101 MHz, DMSO-d_6) δ 186.76, 171.33, 161.87, 160.92, 141.55, 136.39, 135.10, 134.22, 126.41, 124.67, 124.45, 122.95, 121.31, 119.67, 118.35, 115.31, 111.50, 109.65, 101.24, 61.91, 13.92. Anal. calcd for $C_{23}H_{18}N_2O_4$: C, 71.49; H, 4.70; N, 7.25%. Found: C, 70.99; H, 4.71; N, 7.23%.

*Ethyl (2Z,5E)-2-hydroxy-4-oxo-6-(1-(4-(trifluoromethyl)phenyl)-1H-indol-3-yl)hexa-2,5-dienoate (**6h**)*. Compound **6h** was prepared from **10h** by means of GP-C; 20 min; ethanol; 98% as red solid; decomposes at 115 °C; IR ν CO ester 1721, CO ketone 1524 cm^{-1}; ^{1}H NMR (400 MHz, DMSO-d_6) δ 8.48 (s, 1H, indole C2-H), 8.29 – 8.27 (m, 1H, indole C4-H), 8.09 (d, *J* = 15.9 Hz, 1H, hexenoate C6-H), 8.01 (d, *J* = 8.3 Hz, 2H, benzene H), 7.92 (d, *J* = 8.4 Hz, 2H, benzene H), 7.70 – 7.68 (m, 1H, indole C7-H), 7.40 – 7.38 (m, 2H, indole C5-H and C6-H), 7.14 (d, *J* = 16.0 Hz, 1H, hexenoate C5-H), 6.75 (s, 1H, hexenoate C3-H), 4.30 (q, *J* = 7.2 Hz, 2H, CH_2CH_3), 1.31 (t, *J* = 7.1 Hz, 3H, CH_2CH_3). ^{13}C NMR (101 MHz, DMSO-d_6) δ 186.72, 161.08, 140.98, 136.76, 136.41, 135.19, 127.02, 126.98, 126.10, 124.59, 124.19, 122.65, 119.29, 114.85, 111.24, 101.07, 61.73, 13.73. Anal. calcd for $C_{23}H_{18}F_3NO_4$: C, 64.34; H, 4.23; F, 13.27; N, 3.26%. Found: C, 64.10; H, 4.22; F, 13.25; N, 3.27%.

*(Z)-2-hydroxy-4-(1-(4-methoxyphenyl)-1H-indol-3-yl)-4-oxobut-2-enoic acid (**7a**)*. Compound **7a** was prepared from **8a** by means of GP-D; 30 min; ethanol; 89% as a brown solid; 177 - 180 °C; IR ν OH 3338, CO acid 1615, CO ketone 1512 cm^{-1}; ^{1}H NMR (400 MHz, DMSO-d_6) δ 8.98 (s, 1H, indole C2-H), 8.36 – 8.34 (m, 1H, indole C4-H), 7.63 (d, *J* = 8.7 Hz, 2H, benzene H), 7.49 – 7.47 (m, 1H, indole C7-H), 7.36 – 7.34 (m, 2H, indole C5-H and C6-H), 7.19 – 7.15 (m, 3H, benzene H and butenoate C3-H), 3.86 (s, 3H, CH_3). ^{13}C NMR (101 MHz, DMSO-d_6) δ 189.74, 163.70, 162.93, 158.97, 137.98, 137.18, 130.25, 126.37, 125.90, 124.32, 123.35, 122.07, 115.42, 114.95, 111.49, 100.87, 55.61. Anal. calcd for $C_{19}H_{15}NO_5$: C, 67.65; H, 4.48; N, 4.15%. Found: C, 67.85; H, 4.47; N, 4.16%.

*(Z)-2-hydroxy-4-oxo-4-(1-(p-tolyl)-1H-indol-3-yl)but-2-enoic acid (**7b**)*. Compound **7b** was prepared from **8b** by means of GP-D; 15 min; toluene; 44% as a yellow solid; 180 - 182 °C; IR ν CO acid 1606, CO ketone 1518 cm^{-1}; ^{1}H NMR (400 MHz, DMSO-d_6) δ 9.08 (s, 1H, indole C2-H), 8.44 – 8.41 (m, 1H, indole C4-H), 7.66 (d, *J* = 8.2 Hz, 2H, benzene H), 7.62 – 7.59 (m, 1H, indole C7-H), 7.51 (d, *J* = 8.1 Hz, 2H, benzene H), 7.44 – 7.42 (m, 2H, indole C5-H and C6-H), 7.23 (s,

1H, butenoate C3-H), 2.50 (s, 3H, CH_3). ^{13}C NMR (101 MHz, DMSO-d_6) δ 189.80, 163.73, 163.00, 161.12, 137.83, 137.71, 136.84, 134.95, 130.29, 126.05, 124.66, 124.37, 123.42, 122.12, 115.67, 111.55, 100.88, 20.69. Anal. calcd for $C_{19}H_{15}NO_4$: C, 71.02; H, 4.71; N, 4.36%. Found: C, 70.80; H, 4.70; N, 4.35%.

*(Z)-2-hydroxy-4-(1-(4-isopropylphenyl)-1H-indol-3-yl)-4-oxobut-2-enoic acid (**7c**)*. Compound **7c** was prepared from **8c** by means of GP-D; 15 min; diisopropyl ether; 83% as a yellow solid; decomposes at 135 °C; IR ν OH 2960, CO acid 1603, CO ketone 1515 cm^{-1}; ^1H NMR (400 MHz, DMSO-d_6) δ 15.29 (bs, 1H, OH), 13.83 (bs, 1H, OH), 9.01 (s, 1H, indole C2-H), 8.37 – 8.35 (m, 1H, indole C4-H), 7.65 – 7.51 (m, 5H, benzene H and indole C7-H), 7.37 – 7.35 (m, 2H, indole C5-H and C6-H), 7.14 (s, 1H, butenoate C3-H), 3.06 – 3.02 (m, 1H, CH), 1.29 (d, J = 6.9 Hz, 6H, CH_3). ^{13}C NMR (101 MHz, CD_3OD-d_4) δ 199.04, 171.67, 171.44, 158.19, 147.60, 146.39, 144.66, 137.19, 135.43, 134.36, 133.92, 132.95, 131.57, 125.02, 121.08, 110.50, 42.64, 33.31. Anal. calcd for $C_{21}H_{19}NO_4$: C, 72.19; H, 5.48; N, 4.01%. Found: C, 72.00; H, 5.47; N, 4.02%.

*(Z)-4-(1-(4-fluorophenyl)-1H-indol-3-yl)-2-hydroxy-4-oxobut-2-enoic acid (**7d**)*. Compound **7d** was prepared from **8d** by means of GP-D; 15 min; washed with isopropanol; 57% as a yellow solid; 170 - 173 °C; IR ν OH 3300, CO acid 1621, CO ketone 1512 cm^{-1}; ^1H NMR (400 MHz, DMSO-d_6) δ 9.05 (s, 1H, indole C2-H), 8.38 – 8.35 (m, 1H, indole C4-H), 7.79 (dd, J = 9.0, 4.8 Hz, 2H, benzene H), 7.52 – 7.48 (m, 3H, benzene H and indole C7-H), 7.39 – 7.36 (m, 2H, indole C5-H and C6-H), 7.18 (s, 1H, butenoate C3-H). ^{13}C NMR (101 MHz, DMSO-d_6) δ 189.87, 163.71, 163.06, 137.72, 137.43, 136.72, 129.90, 128.23, 126.10, 124.84, 124.44, 123.47, 122.15, 115.86, 111.54, 100.87. Anal. calcd for $C_{18}H_{12}FNO_4$: C, 66.46; H, 3.72; F, 5.84; N, 4.31%. Found: C, 66.60; H, 3.71 F, 5.85 N, 4.30%.

*(Z)-2-hydroxy-4-oxo-4-(1-phenyl-1H-indol-3-yl)but-2-enoic acid (**7e**)*. Compound **7e** was prepared from **8e** by means of GP-D; 1 h; ethanol; 58% as a green solid; decomposes at 197 °C; IR ν CO acid 1621, CO ketone 1513 cm^{-1}; ^1H NMR (400 MHz, DMSO-d_6) δ 15.31 (bs, 1H, OH), 13.79 (bs, 1H, OH), 9.07 (s, 1H, indole C2-H), 8.38 – 8.36 (m, 1H, indole C4-H), 7.73 (d, J = 7.7 Hz, 2H benzene H), 7.65 (t, J = 7.7 Hz, 2H, benzene H), 7.59 – 7.53 (m, 2H, benzene H and indole C7-H), 7.38 – 7.36 (m, 2H, indole C5-H and C6-H), 7.18 (s, 1H, butenoate C3-H). ^{13}C NMR (101 MHz, DMSO-d_6) δ 189.86, 163.73, 162.59, 160.16, 137.86, 136.92, 133.81, 127.26, 127.17, 125.97, 124.44, 123.45, 122.11, 116.82, 116.59, 115.81, 111.40, 100.76. Anal. calcd for $C_{18}H_{13}NO_4$: C, 70.35; H, 4.26; N, 4.56%. Found: C, 70.50; H, 4.27; N, 4.55%.

*Ethyl (Z)-2-hydroxy-4-(1-(4-methoxyphenyl)-1H-indol-3-yl)-4-oxobut-2-enoate (**8a**)*. Compound **8a** was prepared from **11a** by means of GP-C; 30 min; toluene; 75% as a yellow solid; 147 - 150 °C; IR ν OH 3385, CO ester 1736, CO ketone 1512 cm^{-1}; ^1H NMR (400 MHz, DMSO-d_6) δ 9.02 (s, 1H, indole C2-H), 8.36 – 8.34 (m, 1H, indole C4-H), 7.64 (d, J = 8.6 Hz, 2H, benzene H), 7.48 – 7.46 (m, 1H, indole C7-H), 7.37 – 7.35 (m, 2H, indole C5-H and C6-H), 7.20 – 7.16 (m, 3H, benzene H and butenoate C3-H), 4.31 (q, J = 7.1 Hz, 2H, CH_2CH$_3$), 3.86 (s, 3H, CH_3), 1.31 (t, J = 7.1 Hz, 3H, CH$_2$$CH_3$). ^{13}C NMR (101 MHz, DMSO-d_6) δ 189.54, 162.27, 162.02, 159.08, 138.29, 137.31, 130.23, 126.51, 125.86, 124.43, 123.47, 122.10, 115.35, 115.01, 111.54, 101.05, 61.89, 55.65, 14.00. Anal. calcd for $C_{21}H_{19}NO_5$: C, 69.03; H, 5.24; N, 3.83%. Found: C, 68.88; H, 5.22; N, 3.82%.

*Ethyl (Z)-2-hydroxy-4-oxo-4-(1-(p-tolyl)-1H-indol-3-yl)but-2-enoate (**8b**)*. Compound **8b** was prepared from **11b** by means of GP-C; 15 min; washed with isopropanol; 67% as a yellow solid; decomposes at 109 °C; IR ν CO ester 1737, CO ketone 1517 cm^{-1}; ^1H NMR (400 MHz, DMSO-d_6) δ 9.05 (s, 1H, indole C2-H), 8.37 – 8.34 (m, 1H, indole C4-H), 7.60 (d, J = 8.2 Hz, 2H, benzene H), 7.54 – 7.52 (m, 1H, indole C7-H), 7.46 (d, J = 8.1 Hz, 2H, benzene H), 7.37 – 7.35 (m, 2H, indole C5-H and C6-H), 7.18 (s, 1H, butenoate C3-H), 4.31 (q, J = 7.1 Hz, 2H, CH_2CH$_3$), 2.43 (s, 3H, CH_3), 1.31 (t, J = 7.1 Hz, 3H, CH$_2$$CH_3$). ^{13}C NMR (101 MHz, DMSO-d_6) δ 189.55, 162.21, 162.01, 138.02, 137.93, 136.92, 134.88, 130.29, 125.96, 124.76, 124.43, 123.47, 122.09, 115.52, 111.55, 101.01, 61.83, 20.68, 13.96. Anal. calcd for $C_{21}H_{19}NO_4$: C, 72.19; H, 5.48; N, 4.01%. Found: C, 71.99; H, 5.49; N, 4.00%.

*Ethyl (Z)-2-hydroxy-4-(1-(4-isopropylphenyl)-1H-indol-3-yl)-4-oxobut-2-enoate (**8c**)*. Compound **8c** was prepared from **11c** by means of GP-C; 30 min; washed with isopropanol; 56% as a yellow solid; decomposes at 113 °C; IR ν CO ester 1738, CO ketone 1515 cm^{-1}; ^1H NMR (400 MHz, DMSO-d_6) δ 9.06 (s, 1H, indole C2-H), 8.36 – 8.34 (m, 1H, indole C4-H), 7.63 (d, J = 8.4 Hz, 2H, benzene H), 7.54 – 7.51 (m, 3H, benzene H and indole C7-H), 7.38 – 7.35 (m, 2H, indole C5-H and C6-H), 7.17 (s, 1H, butenoate C3-H), 4.30 (q, J = 7.1 Hz, 2H, CH_2CH$_3$), 3.09 – 3.00 (m, 1H, CH), 1.33 – 1.28 (m, 9H, CH$_3$ and CH$_2$$CH_3$). ^{13}C NMR (101 MHz, DMSO-d_6) δ 189.56, 162.19, 161.96, 148.71, 138.12, 136.91, 135.16, 127.72, 125.95, 124.88, 124.44, 123.47, 122.09, 115.54, 111.59, 101.02, 61.82, 33.16, 23.83, 13.95. Anal. calcd for $C_{23}H_{23}NO_4$: C, 73.19; H, 6.14; N, 3.71%. Found: C, 73.30; H, 6.13; N, 3.70%.

*Ethyl (Z)-4-(1-(4-fluorophenyl)-1H-indol-3-yl)-2-hydroxy-4-oxobut-2-enoate (**8d**)*. Compound **8d** was prepared from **11d** by means of GP-C 15 min; ethanol; 79% as a yellow solid; 190 - 193 °C;

IR ν CO ester 1736, CO ketone 1502 cm^{-1}; ^{1}H NMR (400 MHz, DMSO-d_6) δ 9.10 (s, 1H, indole C2-H), 8.37 – 8.35 (m, 1H, indole C4-H), 7.79 (dd, *J* = 9.0, 4.8 Hz, 2H, benzene H), 7.53 – 7.48 (m, 3H, benzene H and indole C7-H), 7.39 – 7.36 (m, 2H, indole C5-H and C6-H), 7.18 (s, 1H, butenoate C3-H), 4.30 (q, *J* = 7.1 Hz, 2H, CH_2CH3), 1.31 (t, *J* = 7.1 Hz, 3H, CH_2CH_3). ^{13}C NMR (101 MHz, DMSO-d_6) δ 189.65, 162.20, 162.02, 138.04, 137.38, 136.83, 129.93, 128.34, 126.02, 124.97, 124.51, 123.55, 122.14, 115.73, 111.55, 101.04, 61.85, 13.97. Anal. calcd for $C_{20}H_{16}FNO_4$: C, 67.98; H, 4.56; F, 5.38; N, 3.96%. Found: C, 67.89; H, 4.55; F, 5.39; N, 3.95%.

*Ethyl (Z)-2-hydroxy-4-oxo-4-(1-phenyl-1H-indol-3-yl)but-2-enoate (**8e**)*. Compound **8e** was prepared from **11e** by means of GP-C; 45 min; isopropanol; 89% as a yellow solid; decomposes at 155 °C; IR ν CO ester 1732, CO ketone 1512 cm^{-1}; ^{1}H NMR (400 MHz, DMSO-d_6) δ 15.40 (bs, 1H, OH), 9.10 (s, 1H, indole C2-H), 8.38 – 8.35 (m, 1H, indole C4-H), 7.73 (d, *J* = 7.9 Hz, 2H, benzene H), 7.66 (t, *J* = 7.6 Hz, 2H, benzene H), 7.57 – 7.54 (m, 2H, benzene H and indole C7-H), 7.39 – 7.36 (m, 2H, indole C5-H and C6-H), 7.19 (s, 1H, butenoate C3-H), 4.31 (q, *J* = 7.1 Hz, 2H, CH_2CH3), 1.32 (t, *J* = 7.1 Hz, 3H, CH_2CH_3). ^{13}C NMR (101 MHz, DMSO-d_6) δ 189.64, 162.19, 162.00, 161.32, 138.28, 137.05, 133.75, 127.43, 127.34, 125.87, 124.56, 123.58, 122.10, 116.87, 116.64, 115.67, 111.46, 101.02, 61.87, 13.98. Anal. calcd for $C_{20}H_{17}NO_4$: C, 71.63; H, 5.11; N, 4.18%. Found: C, 71.56; H, 5.10; N, 4.17%.

*1-(4-methoxyphenyl)-1H-indole-3-carbaldehyde (**9a**)*. Compound **9a** was prepared from 1*H*-indole-3-carboxaldehyde by means of GP-A using 4-iodoanisole as arylating agent; *n*-hexane/ethyl acetate 5:5; 56% as a red solid. Analytical and spectroscopic data are in agreement with literature.[3]

*1-(p-tolyl)-1H-indole-3-carbaldehyde (**9b**)*. Compound **9b** was prepared from 1*H*-indole-3-carboxaldehyde by means of GP-A using 4-iodotoluene as arylating agent; chloroform; 73% as a red solid. Analytical and spectroscopic data are in agreement with literature.[3]

*1-(4-isopropylphenyl)-1H-indole-3-carbaldehyde (**9c**)*. Compound **9c** was prepared from 1*H*-indole-3-carboxaldehyde by means of GP-A using 1-iodo-4-isopropylbenzene as arylating agent; chloroform; diisopropyl ether; 35% as a white wax; IR ν CO 1660 cm^{-1}; ^{1}H NMR (400 MHz, DMSO-d_6) δ 10.0 (s, 1H, CHO), 8.57 (s, 1H, indole C2-H), 8.22 – 8.19 (m 1H, indole C4-H), 7.59 (d, *J* = 8.5 Hz, 2H, benzene H), 7.53 – 7.50 (m, 3H, benzene H and indole C7-H), 7.35 – 7.33 (m, 2H, indole C5-H and C6-H), 3.04 – 2.99 (m, 1H, CH), 1.27 (6H, dd, *J* = 6.9, 1.6 Hz, CH_3). Anal. calcd for $C_{18}H_{17}NO$: C, 82.10; H, 6.51; N, 5.32%. Found: C, 82.08; H, 6.50; N, 5.31%.

*4-(3-formyl-1H-indol-1-yl)benzonitrile (**9g**)*. Compound **9g** was prepared from 1*H*-indole-3-carboxaldehyde by means of GP-A using 4-iodobenzonitrile as arylating agent; *n*-hexane/ethyl acetate 2:1; 27% as a yellow solid. Analytical and spectroscopic data are in agreement with literature.[3]

*1-(4-(trifluoromethyl)phenyl)-1H-indole-3-carbaldehyde (**9h**)*. Compound **9h** was prepared from 1*H*-indole-3-carboxaldehyde by means of GP-A using 4-iodobenzotrifluoride as arylating agent; chloroform; toluene; 76% as a white solid; 168 - 171 °C; IR ν CO 1666 cm^{-1}; ^{1}H NMR (400 MHz, DMSO-d_6) δ 10.1 (s, 1H, CHO), 8.71 (s, 1H, indole C2-H), 8.25 – 8.22 (d, *J* = 7.2 Hz, 1H, indole C4-H), 8.02 (d, *J* = 8.4 Hz, 2H, benzene H), 7.96 (d, *J* = 8.4 Hz, 2H, benzene H), 7.67 – 7.65 (m, 1H, indole C7-H), 7.41 – 7.36 (m, 2H, indole C5-H and C6-H). Anal. calcd for $C_{16}H_{10}F_3NO$: C, 66.44; H, 3.48; F, 19.70; N, 4.84%. Found: C, 66.38; H, 3.47; F, 19.67; N, 4.83%.

*(E)-4-(1-(4-methoxyphenyl)-1H-indol-3-yl)but-3-en-2-one (**10a**)*. Compound **10a** was prepared from **9a** by means of GP-B; chlorofom/ethyl acetate 7:3; isopropanol; 90% as a yellow solid; 85 - 87 °C; IR ν CO 1671 cm^{-1}; ^{1}H NMR (400 MHz, DMSO-d_6) δ 8.19 (s, 1H, indole C2-H), 8.06 – 8.04 (m, 1H, indole C4-H), 7.87 (d, *J* = 16.3 Hz, 1H, butenoate C4-H), 7.53 (d, *J* = 8.0 Hz, 2H, benzene H), 7.47 – 7.44 (m, 1H, indole C7-H), 7.30 – 7.28 (m, 2H, indole C5-H and C6-H), 7.14 (d, *J* = 8.0 Hz, 2H, benzene H), 6.82 (d, *J* = 16.3 Hz, 1H, butenoate C3-H), 3.85 (s, 2H, OCH_3), 2.33 (s, 3H, butenoate C1-H). Anal. calcd for $C_{19}H_{17}NO_2$: C, 78.33; H, 5.88; N, 4.81%. Found: C, 78.29; H, 5.87; N, 4.80%.

*(E)-4-(1-(p-tolyl)-1H-indol-3-yl)but-3-en-2-one (**10b**)*. Compound **10b** was prepared from **9b** by means of GP-B; isopropanol; 76% as a yellow solid; 121 - 123 °C; IR ν CO 1655 cm^{-1}; ^{1}H NMR (400 MHz, DMSO-d_6) δ 8.32 (s, 1H, indole C2-H), 8.07 – 8.04 (m, 1H, indole C4-H), 7.87 (d, *J* = 16.4 Hz, 1H, butenoate C4-H), 7.52 – 7.41 (m, 5H, benzene H and indole C7-H), 7.31 – 7.29 (m, 2H, indole C5-H and C6-H), 6.83 (d, *J* = 16.4 Hz, 1H, butenoate C3-H), 2.41 (s, 3H, CH_3), 2.33 (s, 3H, butenoate C1-H). Anal. calcd for $C_{19}H_{17}NO$: C, 82.88; H, 6.22; N, 5.09%. Found: C, 82.73; H, 6.21; N, 5.08%.

*(E)-4-(1-(4-isopropylphenyl)-1H-indol-3-yl)but-3-en-2-one (**10c**)*. Compound **10c** was prepared from **9c** by means of GP-B; *n*-hexane/ethyl acetate 7:3; diisopropyl ether; 90% as a white wax; IR ν CO 1675 cm^{-1}; ^{1}H NMR (400 MHz, DMSO-d_6) δ 8.24 (s, 1H, indole C2-H), 8.07 – 8.05 (m, 1H, indole C4-H), 7.87 (d, *J* = 16.4 Hz, 1H, butenoate C4-H), 7.55 – 7.47 (m, 5H, benzene H and indole C7-H), 7.31 – 7.29 (m, 2H, indole C5-H and C6-H), 6.83 (d, *J* = 16.4 Hz, 1H, butenoate

C3-H), 3.03 – 3.00 (m, 1H, CH), 2.33 (s, 3H, butenoate C1-H), 1.27 (6H, dd, *J* = 6.9, 1.6 Hz, CH_3). Anal. calcd for $C_{21}H_{21}NO$: C, 83.13; H, 6.98; N, 4.62%. Found: C, 83.02; H, 6.97; N, 4.61%.

*(E)-4-(3-(3-oxobut-1-en-1-yl)-1H-indol-1-yl)benzonitrile (**10g**)*. Compound **10g** was prepared from **9g** by means of GP-B; toluene; 56% as a yellow solid; 205 - 207 °C; IR ν CN 2226 and CO 1671 cm^{-1}; ^1H NMR (400 MHz, DMSO-d_6) δ 8.45 (s, 1H, indole C2-H), 8.16 – 8.14 (m, 3H, benzene H and indole C4-H), 7.96 – 7.90 (m, 3H, benzene H and butanoate C4-H), 7.75 – 7.70 (m, 1H, indole C7-H), 7.41 – 7.40 (m, 2H, indole C5-H and C6-H), 6.94 (d, *J* = 15.9 Hz, 1H, butanoate C3-H), 2.40 (s, 3H, butenoate C1-H), Anal. calcd for $C_{19}H_{14}N_2O$: C, 79.70; H, 4.93; N, 9.78%. Found: C, 79.68; H, 4.92; N, 9.76%.

*(E)-4-(1-(4-(trifluoromethyl)phenyl)-1H-indol-3-yl)but-3-en-2-one (**10h**)*. Compound **10h** was prepared from **9h** by means of GP-B; *n*-hexane/ethyl acetate 7:3; toluene; 68% as a yellow solid; 153 - 155 °C; IR ν CO 1641 cm^{-1}; ^1H NMR (400 MHz, DMSO-d_6) δ 8.38 (s, 1H, indole C2-H), 8.11 – 8.09 (m, 1H, indole C4-H), 7.99 (d, *J* = 8.0 Hz, 2H, benzene H), 7.99 (d, *J* = 8.0 Hz, 2H, benzene H), 7.92 – 7.85 (m, 3H, benzene H and butenoate C4-H), 7.79 – 7.67 (m, 1H, indole C7-H), 7.38 – 7.32 (m, 2H, indole C5-H and C6-H), 6.88 (d, *J* = 16.3 Hz, 1H, butenoate C3-H), 2.35 (s, 3H, butenoate C1-H). Anal. calcd for $C_{19}H_{14}F_3NO$: C, 69.30; H, 4.29; F, 17.31; N, 4.25%. Found: C, 69.27; H, 4.28; F, 17.29; N, 4.24%.

*1-(1-(4-methoxyphenyl)-1H-indol-3-yl)ethan-1-one (**11a**)*. Compound **11a** was prepared from 3-acetylindole by means of GP-A using 4-iodoanisole as arylating agent; *n*-hexane/ethyl acetate 5:5; 53% as a beige solid. Analytical and spectroscopic data are in agreement with literature.[3]

*1-(1-(p-tolyl)-1H-indol-3-yl)ethan-1-one (**11b**)*. Compound **11b** was prepared from 3-acetylindole by means of GP-A using 4-iodotoluene as arylating agent; chloroform; cyclohexane; 57% as a red solid; 135 - 137 °C; IR ν CO 1644 cm^{-1}; ^1H NMR (400 MHz, DMSO-d_6) δ 8.56 (s, 1H, indole C2-H), 8.30 – 8.28 (m, 1H, indole C4-H), 7.55 (d, *J* = 8.3 Hz, 2H, benzene H), 7.50 – 7.47 (m, 1H, indole C7-H), 7.44 (d, *J* = 8.3 Hz, 2H, benzene H), 7.30 – 7.28 (m, 2H, indole C5-H and C6-H), 2.52 (s, 3H, $COCH_3$), 2.42 (s, 3H, CH_3). Anal. calcd for $C_{17}H_{15}NO$: C, 81.90; H, 6.06; N, 5.62%. Found: C, 81.84; H, 6.05; N, 5.61%.

*1-(1-(4-isopropylphenyl)-1H-indol-3-yl)ethan-1-one (**11c**)*. Compound **11c** was prepared from 3-acetylindole by means of GP-A using 1-iodo-4-isopropylbenzene as arylating agent; chloroform; cyclohexane; 65% as a yellow wax; °C; IR ν CO 1647 cm^{-1}; ^1H NMR (400 MHz, DMSO-d_6) δ 8.60 (s, 1H, indole C2-H), 8.30 – 8.28 (m 1H, indole C4-H), 7.59 (d, *J* = 8.5 Hz, 2H, benzene H), 7.52

– 7.50 (m, 3H, benzene H and indole C7-H), 7.30 – 7.28 (m, 2H, indole C5-H and C6-H), 3.04 – 3.01 (m, 1H, CH), 2.51 (s, 3H, $COCH_3$), 1.28 (6H, dd, *J* = 6.9, 1.6 Hz, CH_3). Anal. calcd for $C_{19}H_{19}NO$: C, 82.28; H, 6.90; N, 5.05%. Found: C, 82.25; H, 6.88; N, 5.04%.

*1-(1-(4-fluorophenyl)-1H-indol-3-yl)ethan-1-one (**11d**)*. Compound **11d** was prepared from 3-acetylindole by means of GP-A using 1-fluoro-4-iodobenzene as arylating agent; *n*-hexane/ethyl acetate 5:5; cyclohexane; 79% as a brown solid; 140 - 142 °C; IR ν CO 1630 cm^{-1}; ^1H NMR (400 MHz, DMSO-d_6) δ 8.61 (s, 1H, indole C2-H), 8.31 – 8.29 (m, 1H, indole C4-H), 7.75 – 7.72 (m, 2H, benzene H), 7.51 – 7.45 (m, 3H, benzene H and indole C7-H), 7.31 – 7.29 (m, 2H, indole C5-H and C6-H), 2.52 (s, 3H, $COCH_3$). Anal. calcd for $C_{16}H_{12}FNO$: C, 75.88; H, 4.78; F, 7.50; N, 5.53%. Found: C, 75.80; H, 4.77; F, 7.48; N, 5.52%.

*1-(1-phenyl-1H-indol-3-yl)ethan-1-one (**11e**)*. Compound **11e** was prepared from 3-acetylindole by means of GP-A using iodobenzene as arylating agent; *n*-hexane/ethyl acetate 7:3; 83% as a yellow solid. Analytical and spectroscopic data are in agreement with literature.[3]

Biology Experimental Section

SARS-CoV-2 nsp13 expression and purification.

SARS-CoV-2 nsp13 was expressed from pNIC-ZB vector (addgene 159614)[4] following the procedure described in reference 33 in the main text. Briefly, the protein was express in *E. coli* BL21 Rosetta 2 cells in Terrific Broth media induced with 300 µM IPTG and lasted overnight at 18 °C at 200 rpm.

Cell pellets were resuspended and sonicated for 15 min, 10s on 5s off, and clarified by centrifugation at 11000× g for 50 min. The supernatant was used for a batch binding of 40 min with 3 ml of Ni-sepharose (Cytiva). Beads were loaded on a gravity flow column and washed with 50 ml lysis buffer, 25 ml wash buffer (50 mM HEPES pH 7.5, 500 mM NaCl, 5% Glycerol, 45 mM Imidazole, 0.5 mM TCEP), 10 ml Hi-salt buffer (50 mM HEPES pH 7.5, 1 M NaCl, 5% Glycerol, 0.5 mM TCEP) and again with 10 ml of wash buffer. The protein was eluted with elution buffer (50 mM HEPES pH 7.5, 500 mM NaCl, 5% Glycerol, 300 mM Imidazole, 0.5 mM TCEP). The eluted fraction was immediately applied to a 5ml Hi-Trap SP HP column using a syringe. The column was washed with 20 mL elution buffer and proteins were eluted with 20 mL Hi-salt buffer (20 fractions 0-100% Hi-salt buffer). Protein fractions were pooled and loaded into a Superdex 200

10/300 GL column equilibrated in 50 mM Hepes, 500 mM NaCl, 5% Glycerol and 0.5 mM TCEP. The elution fractions were analyzed for purity by SDS page. Proteins were stored at -80°C.

Determination of SARS-CoV-2 nsp13 unwinding-associated activity.

The SARS-CoV-2 nsp13 unwinding-associated activity was measured as reported in reference 29 in the main text in black 384 well plates (PerkinElmer), in 20 µl reaction volume containing 20 mM Tris–HCl pH 7.2, 50 mM NaCl, 2 µM Hel Capture oligo (5'- TGG TGC TCG AAC AGT GAC - 3') from Biomers, 5 mM $MgCl_2$, 10 µg/ml BSA and 180 µM TCEP, 5% DMSO or inhibitor and 2 nM of purified nsp13. The reaction mixture containing the enzyme was pre-incubated for 10 min with inhibitor at room temperature (RT). The reaction was started adding 500 µM ATP and 500 nM annealed DNA substrate (5'- AGT CTT CTC CTG GTG CTC GAA CAG TGA C-Cy3-3', 5'-BHQ-2-GTC ACT GTT CGA GCA CCA CCT CTT CTG A-3') from Biomers. After 15 min of incubation at 37 °C, fluorescence products were measured with Victor Nivo (Perkin) at 530/580 nm.

Determination of SARS-CoV-2 nsp13 ATPase-associated activity.

The SARS-CoV-2 nsp13 ATPase-associated activity was measured as reported in reference 29 in the main text in a transparent 96 well plate (PerkinElmer), in 25 µl reaction volume containing 20 mM Tris–HCl pH 7.2, 50 mM NaCl, 2 mM $MgCl_2$, 10 µg/ml BSA and 180 µM TCEP, 5% DMSO or inhibitor and 25 nM of purified nsp13. The reaction was started adding 200 µM ATP. After 30 min of incubation at 37 °C, 50 µl of Biomol® Green Reagent (Prod. No. BML-AK111, Enzo Lifescience) were added and reaction was incubated for 10 min at RT, protected from the light. Products were measured with Victor Nivo (Perkin) at 650 nm for ABS value.

Data analysis (enzymatic assays). Data analysis of assay development results was performed using GraphPad Prism Version 9.1.2. Test compound results were normalized relative to respective controls. Dose response curves, with the values expressed as percentage of control from two experiments, each performed in triplicate, were fitted to a non-linear regression of (log10) dose vs normalized response- variable slope, obtaining the IC_{50} values. Assay quality was assessed using the Z′-factor calculation with Z′ > 0.5 as threshold for acceptance.

Data analysis (antiviral activity). The EC_{50} value was determined and graphed by GraphPad Prism v9.0 software by fitting a variable slope-sigmoidal dose-response curve. The data set consists of the viral inhibition percentages of the treated virus at different compound concentrations.

Virus production. A virus working stock was prepared through the propagation in Vero E6 cells cultured in minimum essential medium (MEM) containing 2% (w/v) fetal bovine serum (Euroclone S.p.A.) of a strain of SARS-CoV-2 variant omicron XBB.1.16.11 (hCoV-19/Italy/LAZ-DIBS-230829301/2023) isolated from nasopharyngeal swab by Defence Institute for Biomedical Sciences, Rome Italy. 72 hours after the infection, supernatants containing the released viral particles were collected and centrifuged at 600 g for 5 minutes. Virus stocks were kept at −80 °C until use. The viral titer was determined by plaque assay.

Cellular toxicity assay. Vero E6-green fluorescent protein (GFP) cells (Janssen Pharmaceutical were maintained in Dulbecco's modified Eagle's medium (DMEM, Gibco) supplemented with 10 % v/v fetal beef serum (FBS, Gibco), 0.075 % Sodium Bicarbonate (7.5 % solution, Gibco) and 1X Pen-strep (Euroclone) and kept under 5 % CO_2 on 37 ◦C. The cells were seeded at 10,000 cells/well in 96-well black cell-treated plates (PerkinElmer). The following day, cells were incubated with the control compounds at different concentrations. Compound was dissolved in 0.1 % dimethyl sulfoxide (DMSO). Seventy-two hours post infection the GPF signal, as direct index of cellular viability, was quantified by measuring the total-well fluorescence with a Victor 3 multiplate reader (PerkinElmer) set to excitation and emission wavelengths of 485 and 535 nm, respectively.

The percentage of induced CPE was calculated considering the mock as 100 % of cell viability, and the readout from empty wells as blank. The compound half-maximal cytopathic concentration (CC_{50}), was determined via non-linear regression by using on Prism 9 v. 9.4.1 software (GraphPad) the built-in function dose-response inhibition, log-concentration-normalized response. Experimental points represent the average and standard deviation of at least two sets of independent triplicates.

SARS CoV-2 plaque assay. Confluent Vero E6 cell monolayers were infected for 1 h at 37 ◦C with SARS-CoV-2 (0.01 MOI). Then, after 1-hour incubation, the inoculum was replaced with fresh medium supplemented with 2% FBS and containing respectively each indole derivative at different concentrations. Untreated-infected cells were used as positive control of viral infection. After 24 h, the supernatants were collected, and plaque assay was performed. Briefly, Vero E6 cells seeded in a 12/24-well plate for 1 h at 37 °C. Then, the inoculum was removed, and the medium was replaced with a mixture of MEM (no glutamine, no phenol-red-GIBCO), 1.5% Tragacanth (SIGMA), $NaHCO_3$ 7% (Gibco), L-glutamine 1X (Gibco), MEM NEAA 1x (Gibco), 0.02M Hepes (Euroclone), DMSO (Sigma-Aldrich) and 2% FBS (final concentration). 3 days post-infection the mixture was carefully removed, plates were washed with saline solution, stained with

1% crystal violet for 10 min and plaque forming units (PFU) were counted. The plaque reduction ratio was calculated as (100− N/N0 ×100) where N is the PFU count of the treated sample, and N0 is the PFU count of the control sample, obtaining the viral inhibition percentage. The plaque assay was also used to determine the concentration of molecules that inhibit 50% of viral titer in each well (IC_{50}).

Molecular Modeling Experimental Section

Molecular docking protocol. The 3D model of the SARS-CoV-2 nsp13 helicase protein was generated as previously reported in reference 33 in the main text. Before utilizing these receptor structures in docking calculations, preparation steps were taken using the Protein Preparation Wizard utility within the Maestro software package.[5] The receptor structures underwent preparation, including assigning bond orders, adding hydrogens, and generating physiological pH states using the EPIK tool. Subsequently, the “Minimize and Delete Waters” tool was employed to minimize overall protein structures, with restrained heavy atoms and removal of all water molecules. The model was then validated with Ramachandran plot (Figure S61).[6]

After generating the model, docking calculations were attained for **5c**, **5h**, **6f**, **6g** and **6h** employing the Glide tool implemented in Maestro.[7] To prepare all the ligands for docking calculations, the Schrodinger software suite’s “LigPrep” tool was employed. This included adding hydrogen atoms, generating all tautomeric states, and retaining specified chiralities. The docking grid boxes were centered on RecA2 domain based on previously reported DKA inhibitors (center in -34.42 × 1.69 × -21.5) with a grid box dimension equal to 20 Å × 20 Å × 20 Å. Finally, docking runs were carried out using the standard Glide protocol with a rigid treatment of the protein, employing standard settings. The best-scoring complexes in terms of GlideScore were selected.

All the figures were rendered with Pymol.[8]

References

1. R. Di Santo, R. Costi, M. Artico, G. Miele, A. Lavecchia, E. Novellino, A. Bergamini, R. Cancio, G. Maga, “Arylthiopyrrole (AThP) derivatives as non-nucleoside HIV-1 reverse transcriptase inhibitors: synthesis, structure-activity relationships, and docking studies (part 1).” *ChemMedChem* **2006**, *1*, 1367-1378.

2. R. Costi, G. C. Crucitti, L. Pescatori, A. Messore, L. Scipione, S. Tortorella, A. Amoroso, E. Crespan, P. Campiglia, B. Maresca, A. Porta, I. Granata, E. Novellino, J. Gouge, M. Delarue, G. Maga, R. Di Santo, "New nucleotide-competitive non-nucleoside inhibitors of terminal deoxynucleotidyl transferase: discovery, characterization, and crystal structure in complex with the target." *J. Med. Chem.* **2013,** *56*, 7431–7441.

3. D. Nandi, A. Taher, R. Ul Islam, S. Siwal, M. Choudhary, K. Mallick, "Carbon nitride supported copper nanoparticles: light-induced electronic effect of the support for triazole synthesis." *R. Soc. Open Sci.* **2016**, *3*, 160580.

4. J. A. Newman, A. Douangamath, S. Yadzani, Y. Yosaatmadja, A. Aimon, J. Brandão-Neto, L. Dunnett, T. Gorrie-Stone, R. Skyner, D. Fearon, M. Schapira, F. von Delft, O. Gileadi, "Structure, mechanism and crystallographic fragment screening of the SARS-CoV-2 NSP13 helicase." *Nature Comm.* **2021**, *12*, 4848.

5. Schrödinger Release 2023-1 Maestro, Program for docking studies, Schrödinger, LLC, New York (USA), **2023**.

6. C. J. Williams, J. J Headd, N. W. Moriarty, M. G. Prisant, L. L Videau, L. N. Deis, V. Verma, D. A. Keedy, B. J. Hintze, V. B. Chen, S. Jain, S. M. Lewis, W. B. Arendall 3rd, J. Snoeyink, P. D. Adams, S. C. Lovell, J. S. Richardson, D. C. Richardson, "MolProbity: More and better reference data for improved all-atom structure validation." *Protein Sci*. **2018**, 27(1), 293–315.

7. Schrödinger Release 2023-1 Glide, Program for docking studies, Schrödinger, LLC, New York (USA), **2023**.

8. The PyMOL Molecular Graphics System, Version 2.4.2, Program for docking studies, Schrödinger, LLC, New York (USA), **2019**.

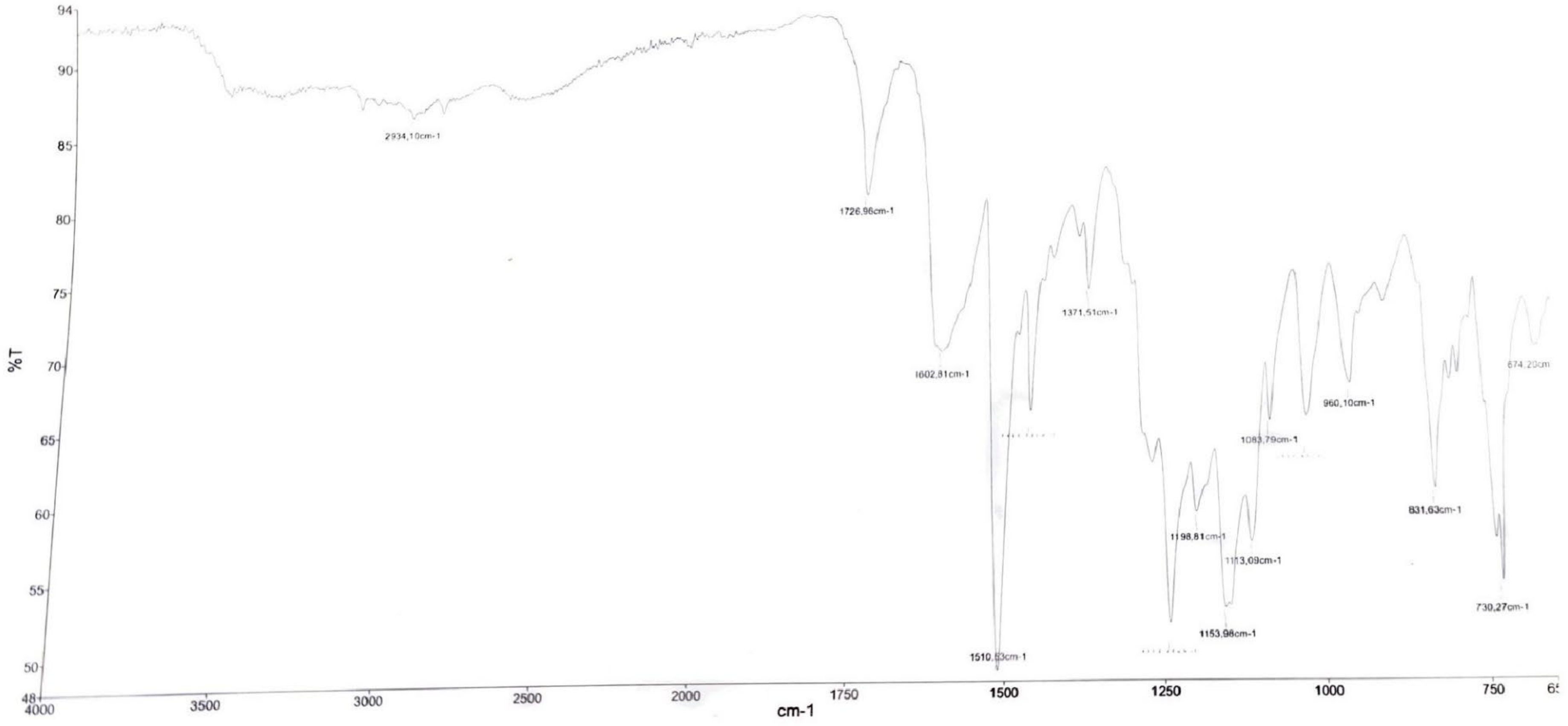

Figure S1. FTIR Spectrum for compound **5a**

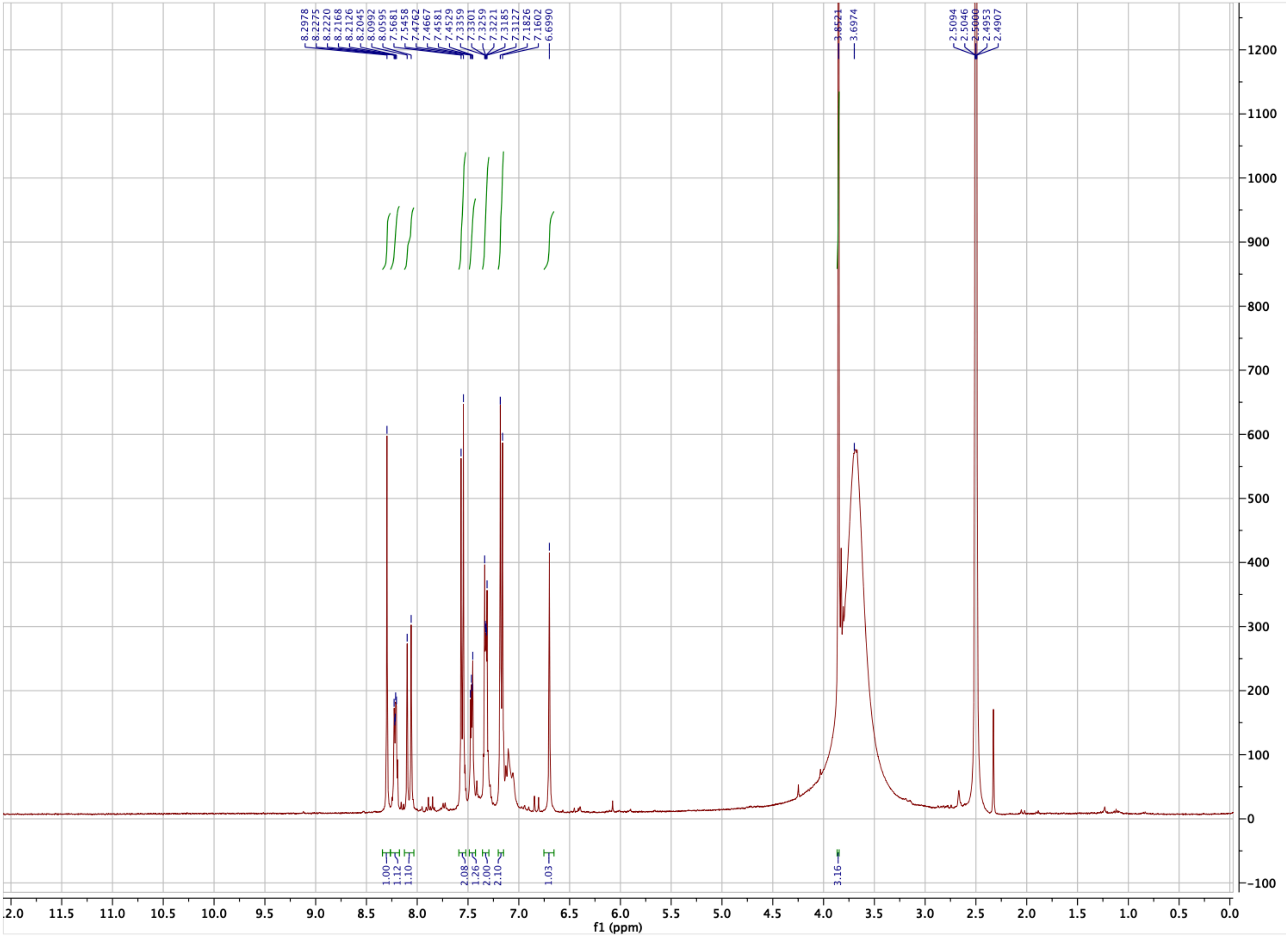

Figure S2. ^{1}H NMR Spectrum for compound **5a**

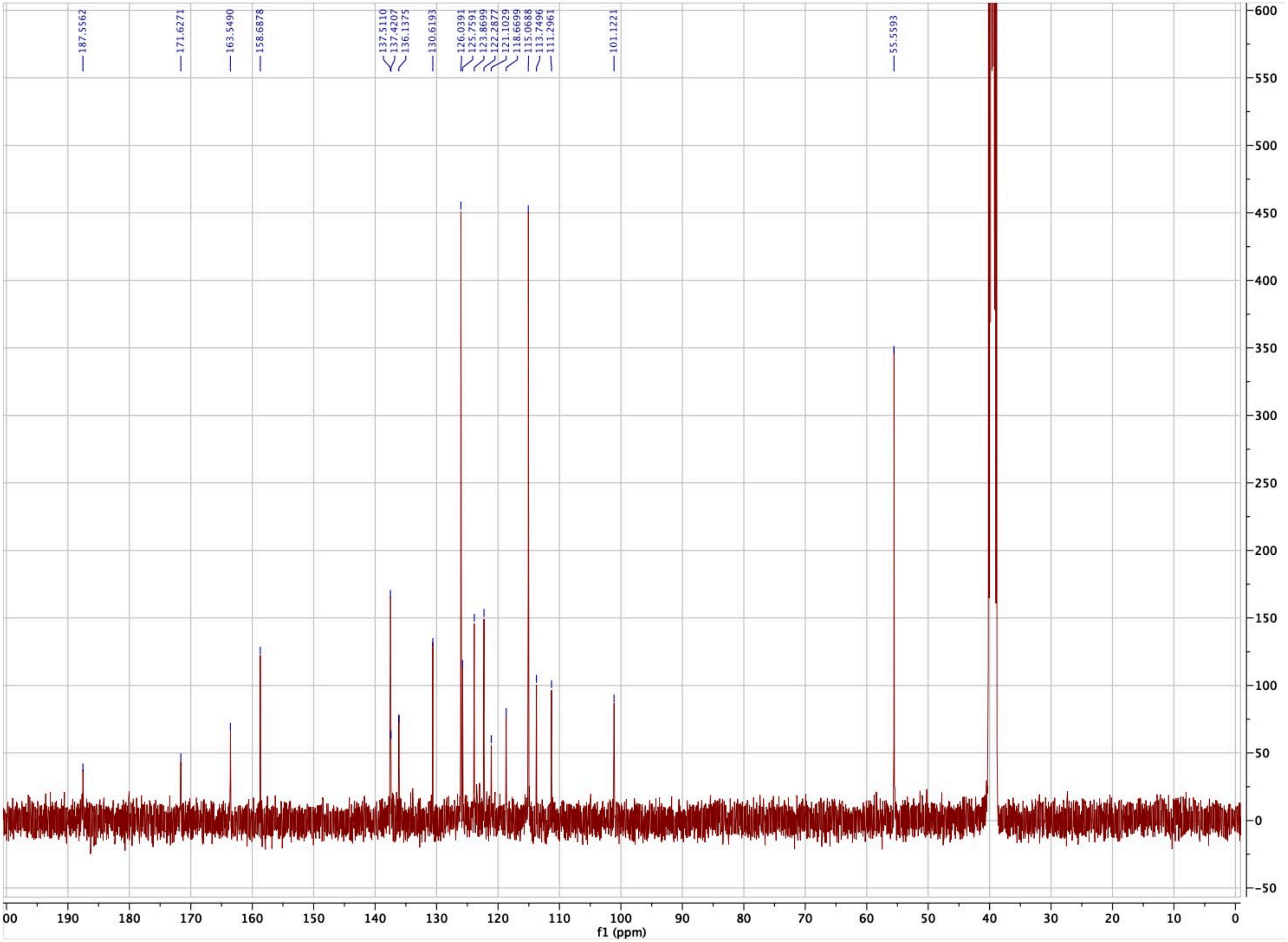

Figure S3. ^{13}C NMR Spectrum for compound **5a**

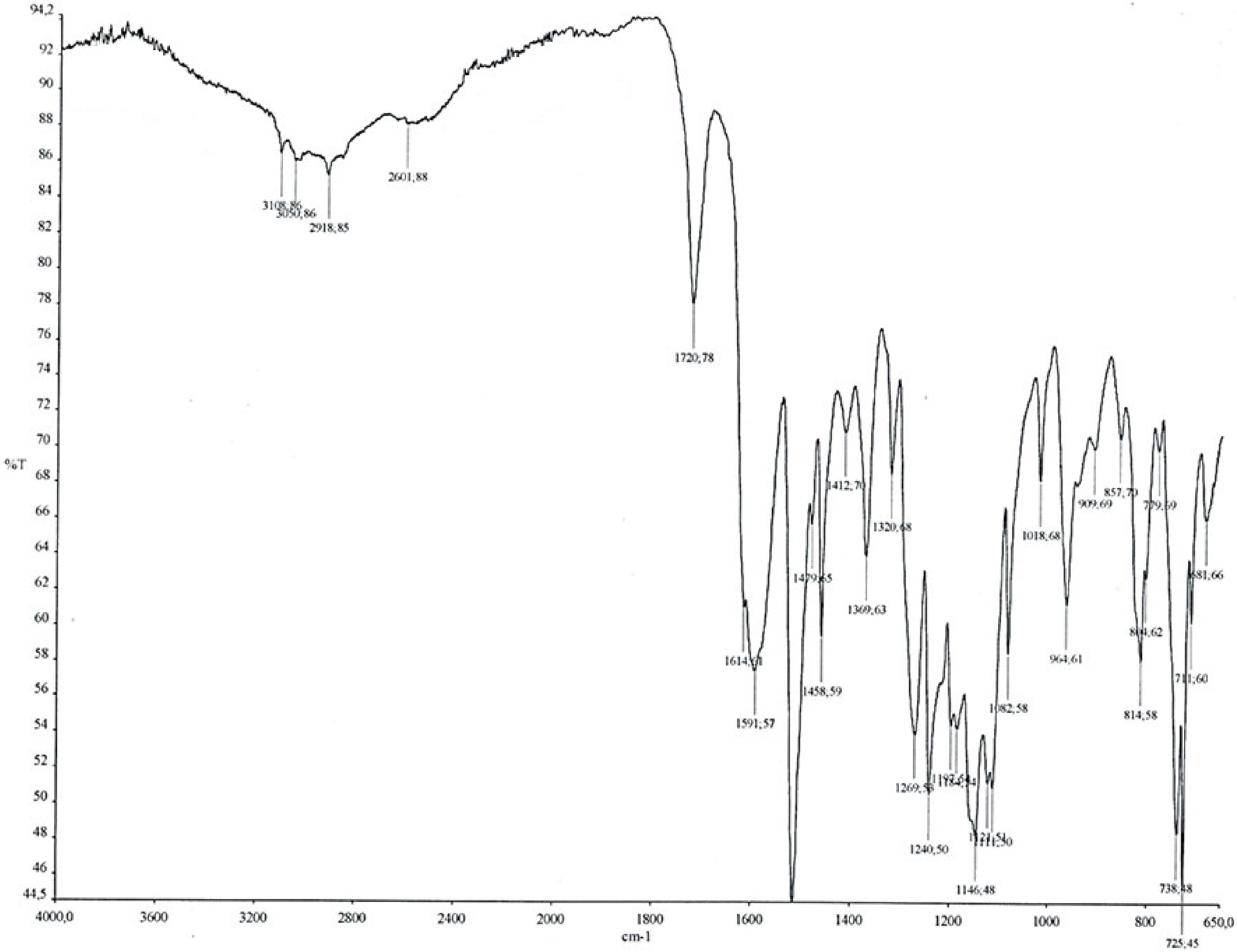

Figure S4. FTIR Spectrum for compound **5b**

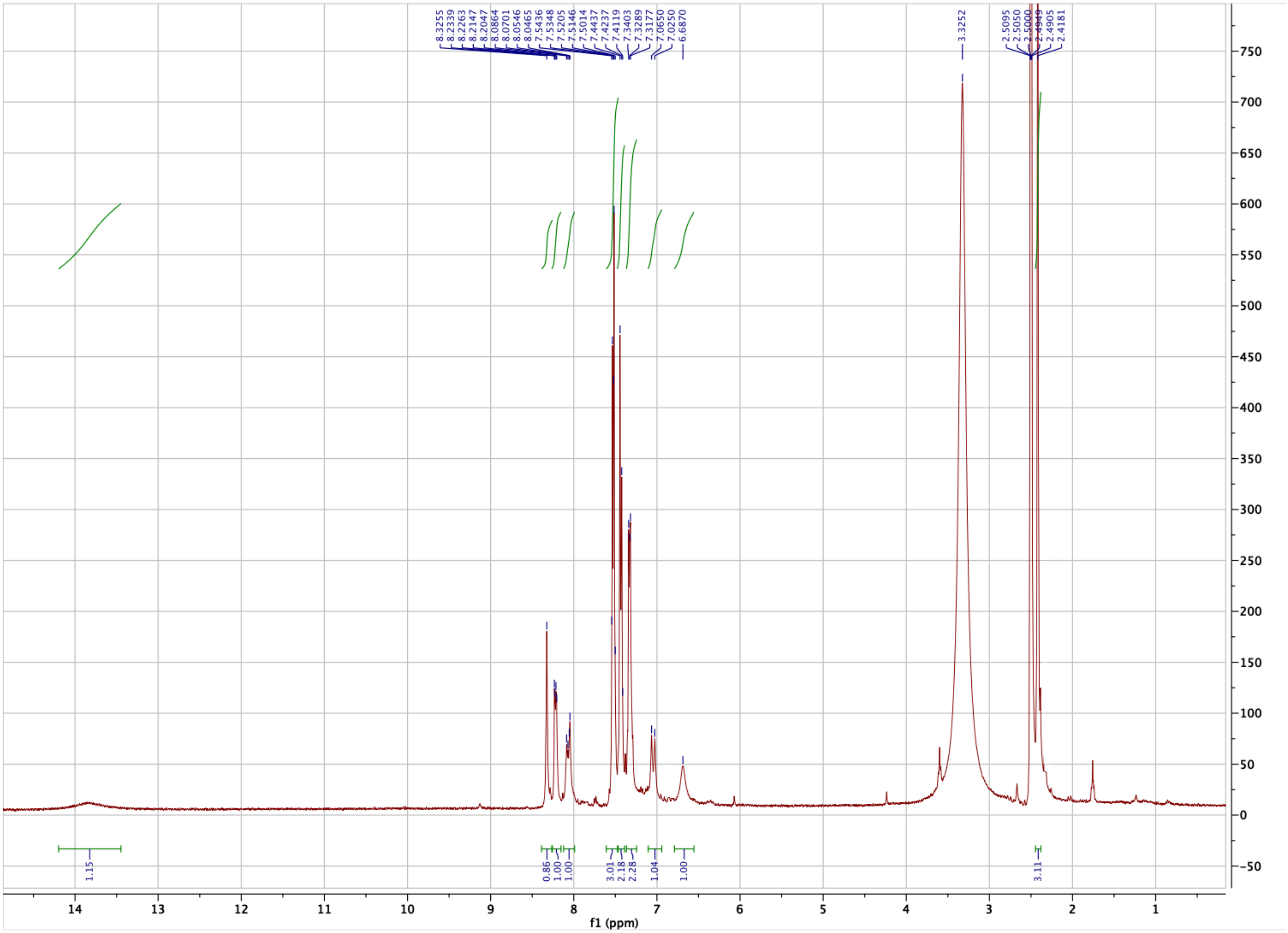

Figure S5. ^{1}H NMR Spectrum for compound **5b**

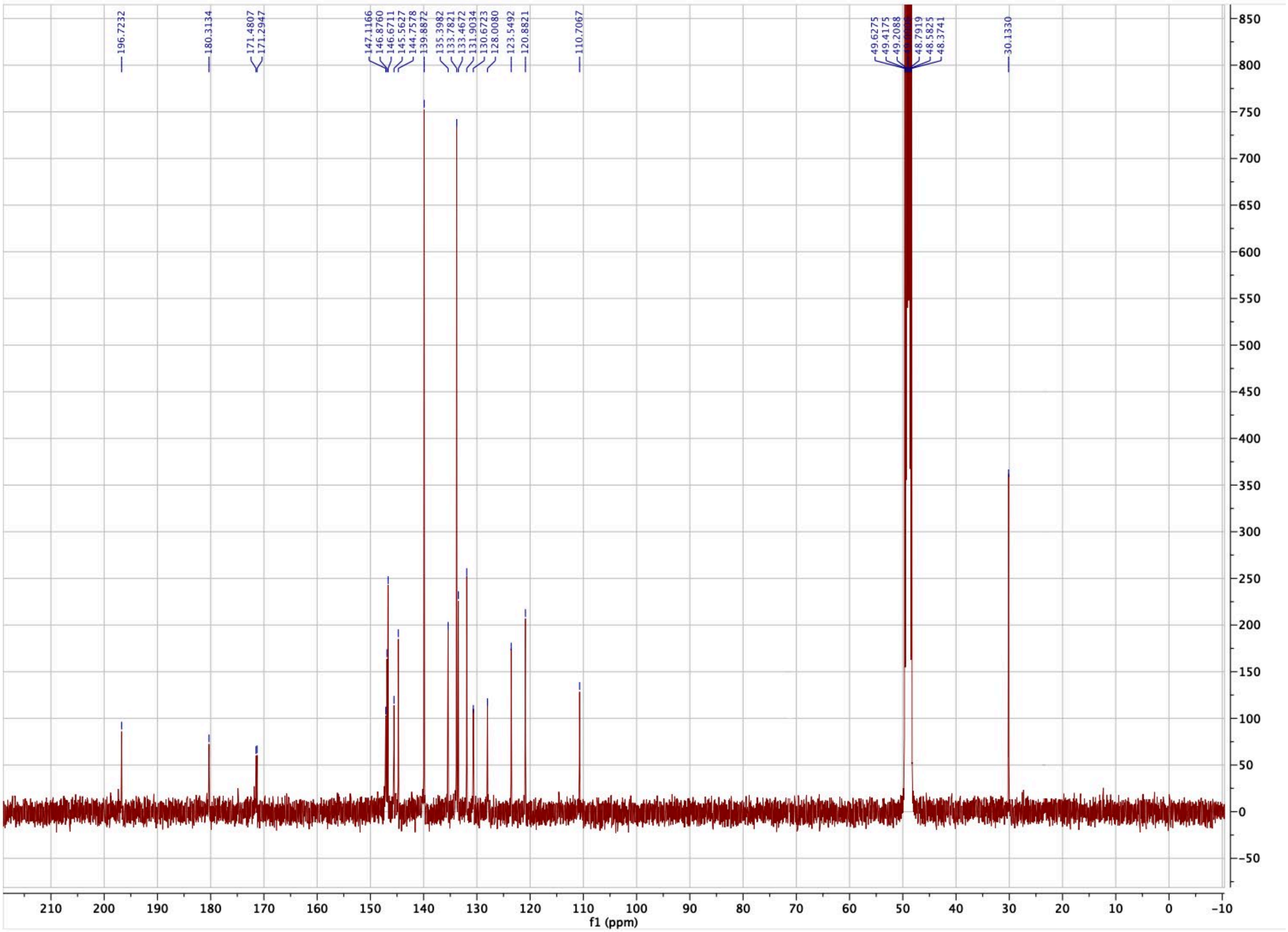

Figure S6. ^{13}C NMR Spectrum for compound **5b**

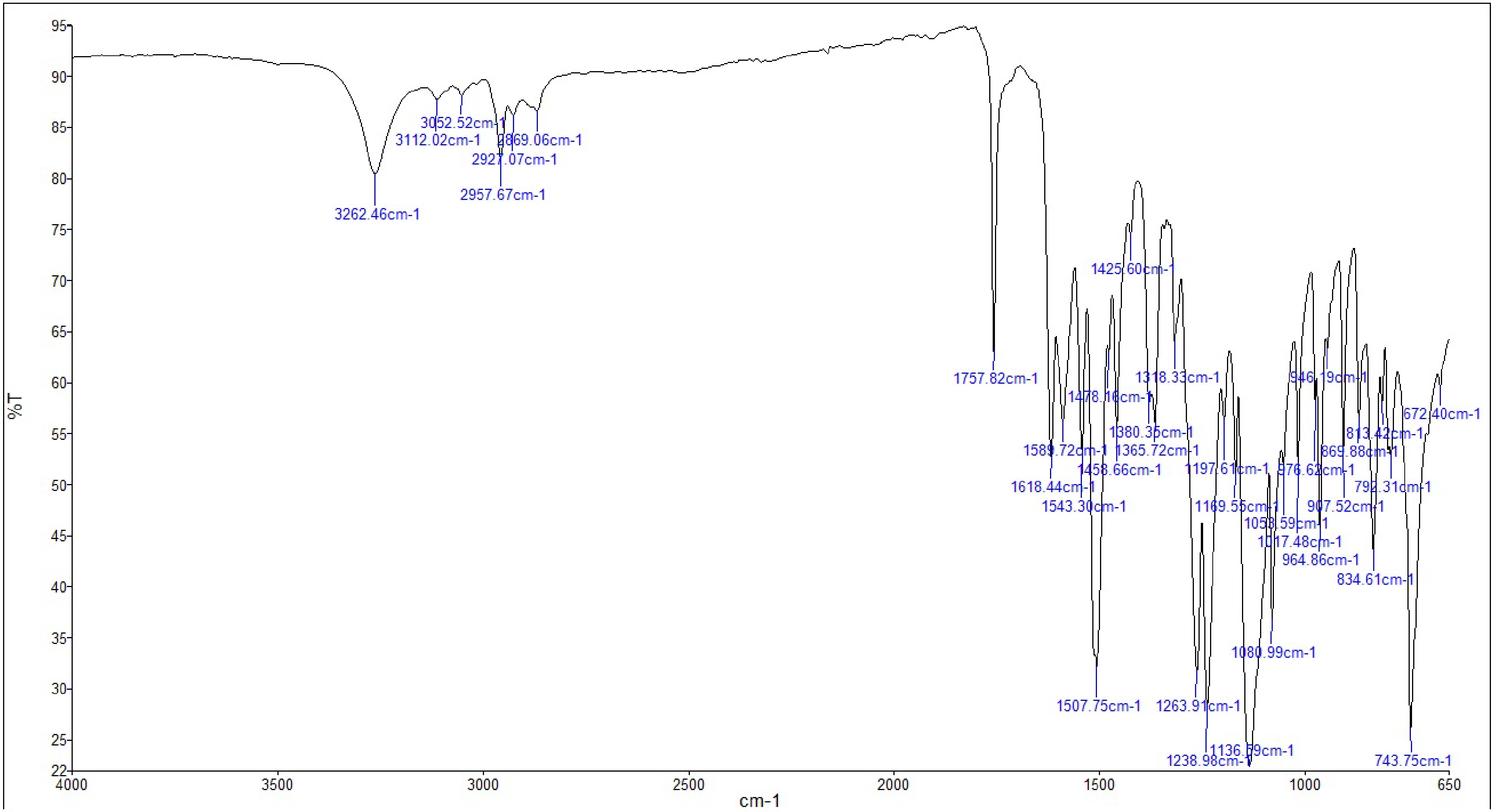

Figure S7. FTIR Spectrum for compound **5c**

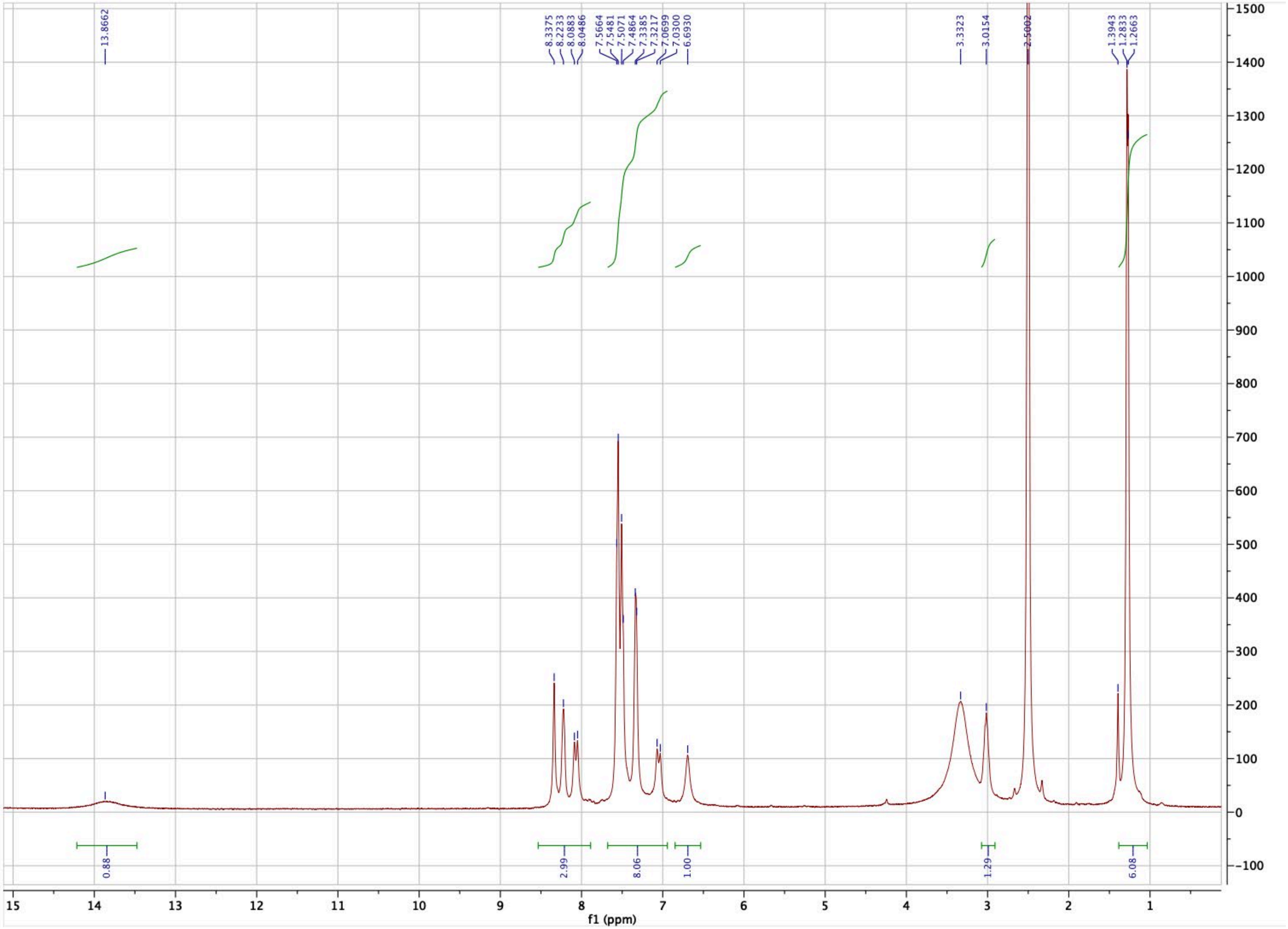

Figure S8. ^{1}H NMR Spectrum for compound **5c**

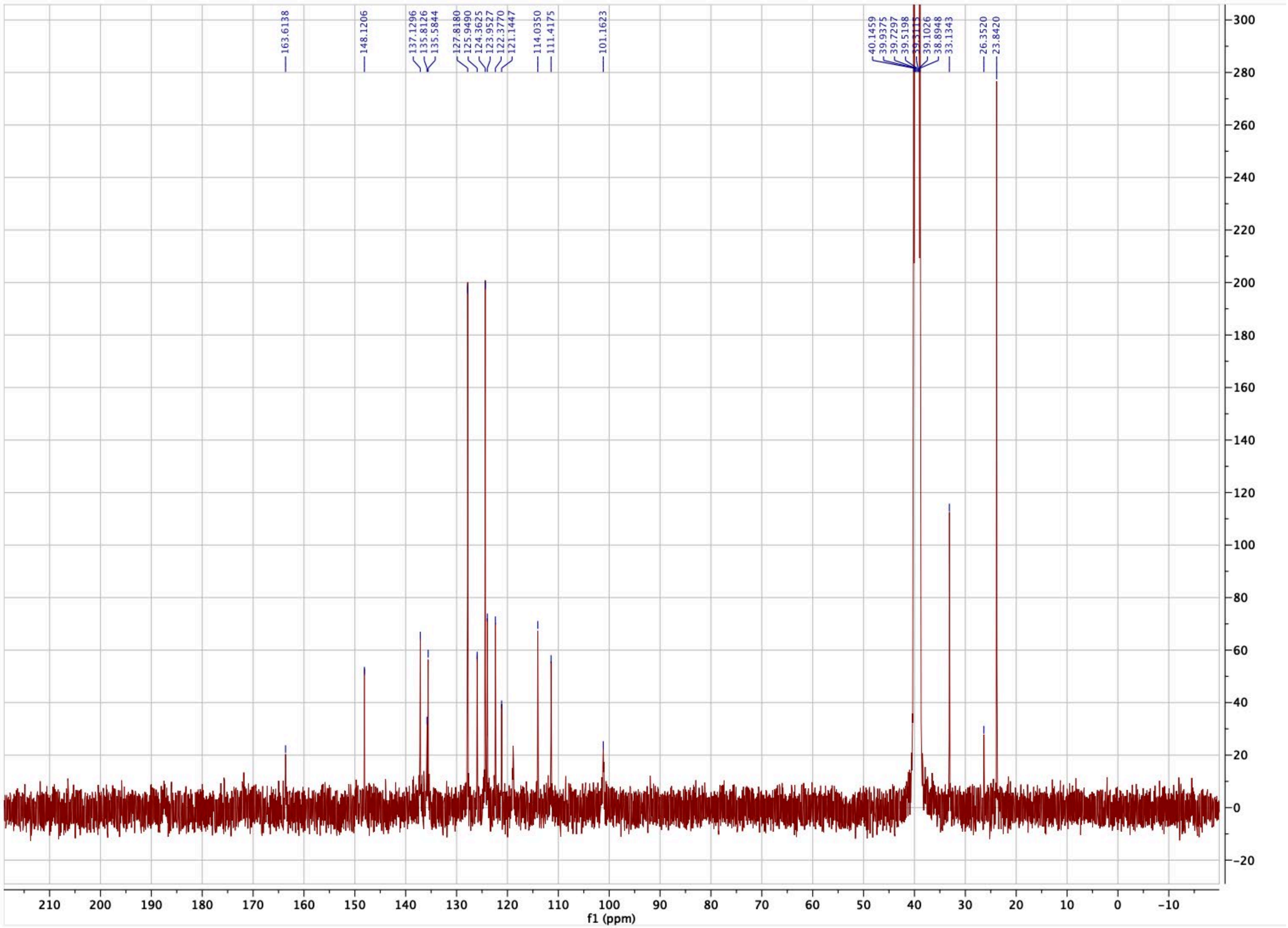

Figure S9. ^{13}C NMR Spectrum for compound **5c**

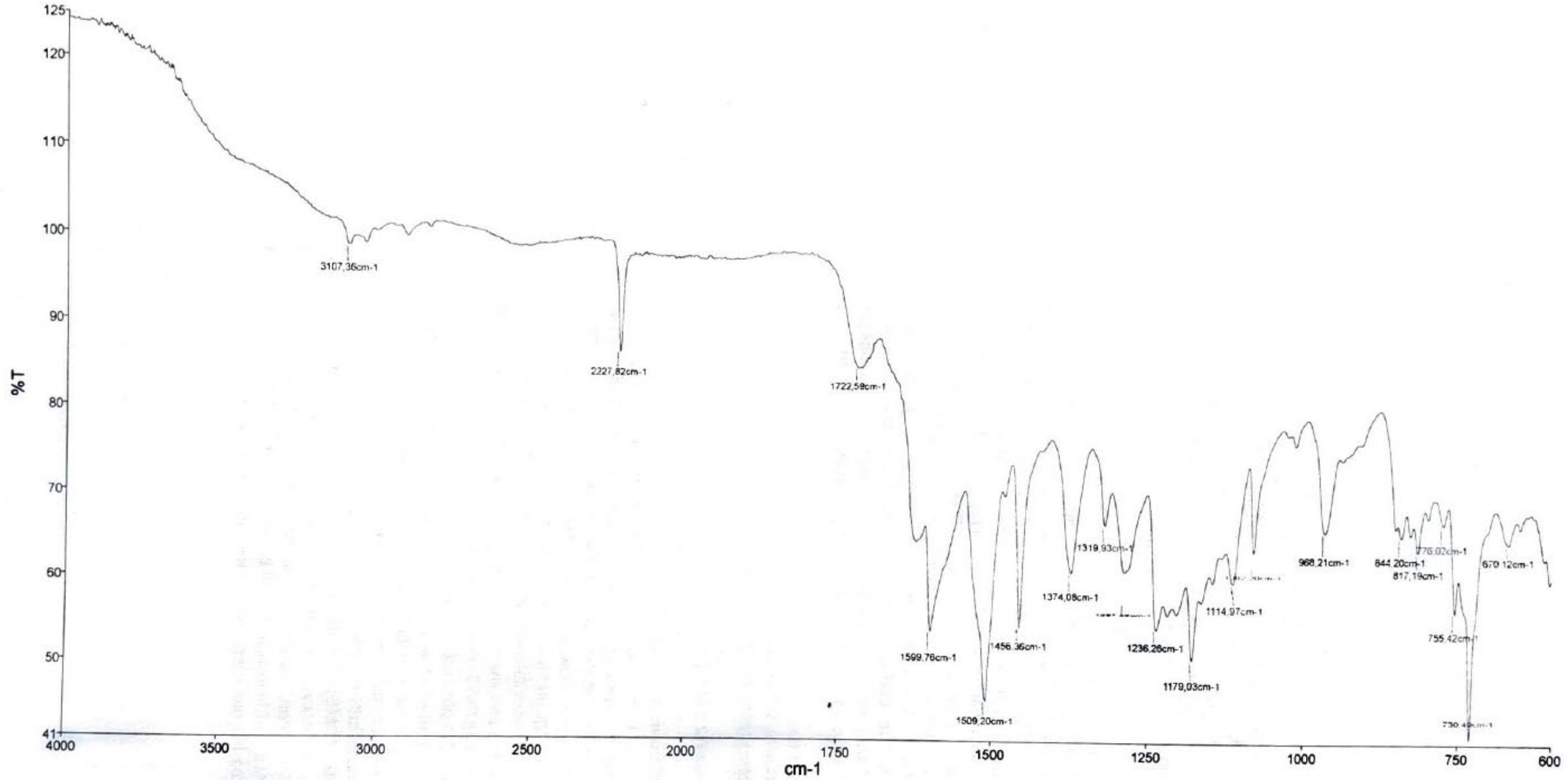

Figure S10. FTIR Spectrum for compound **5g**

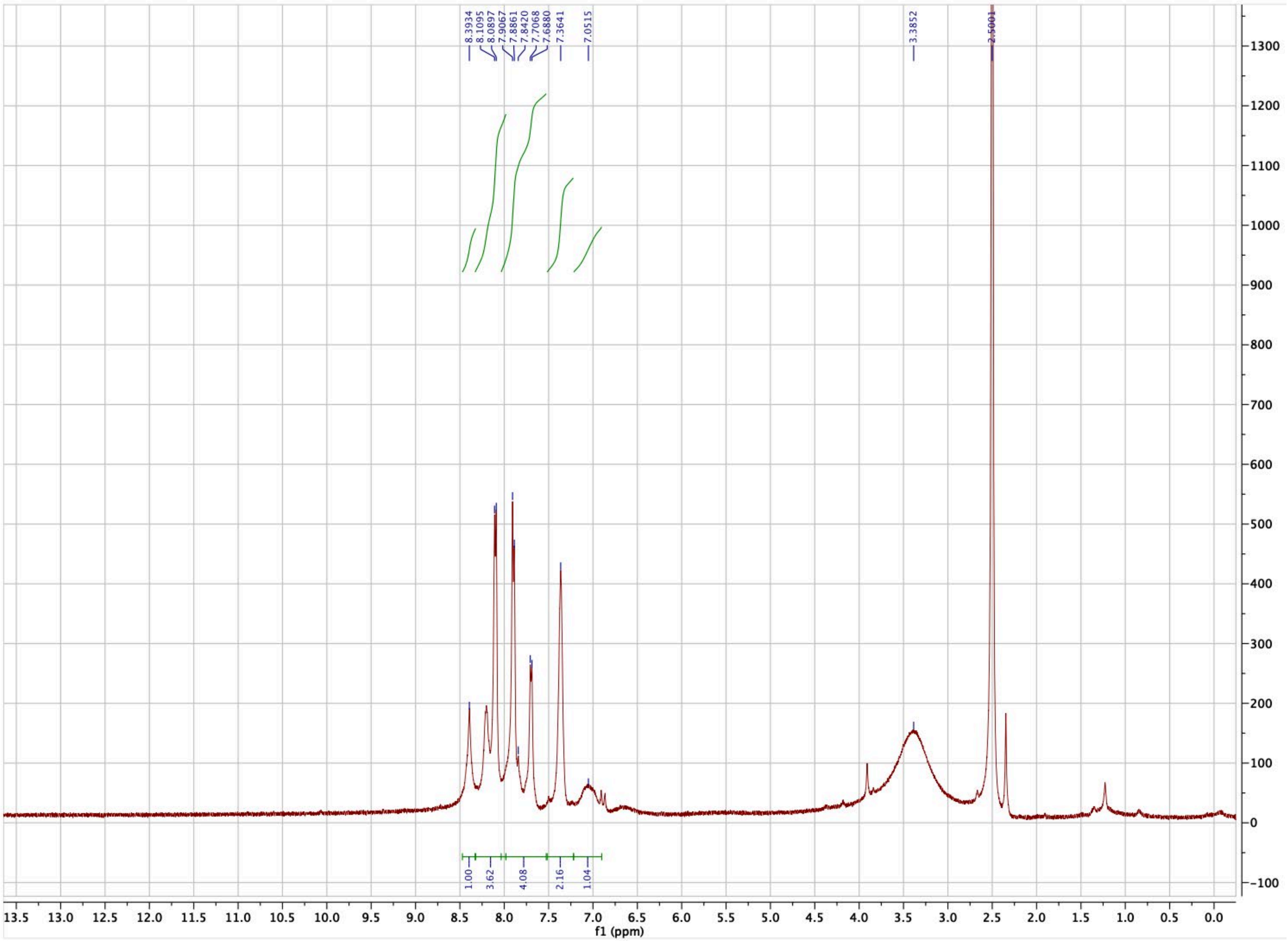

Figure S11. ^{1}H NMR Spectrum for compound **5g**

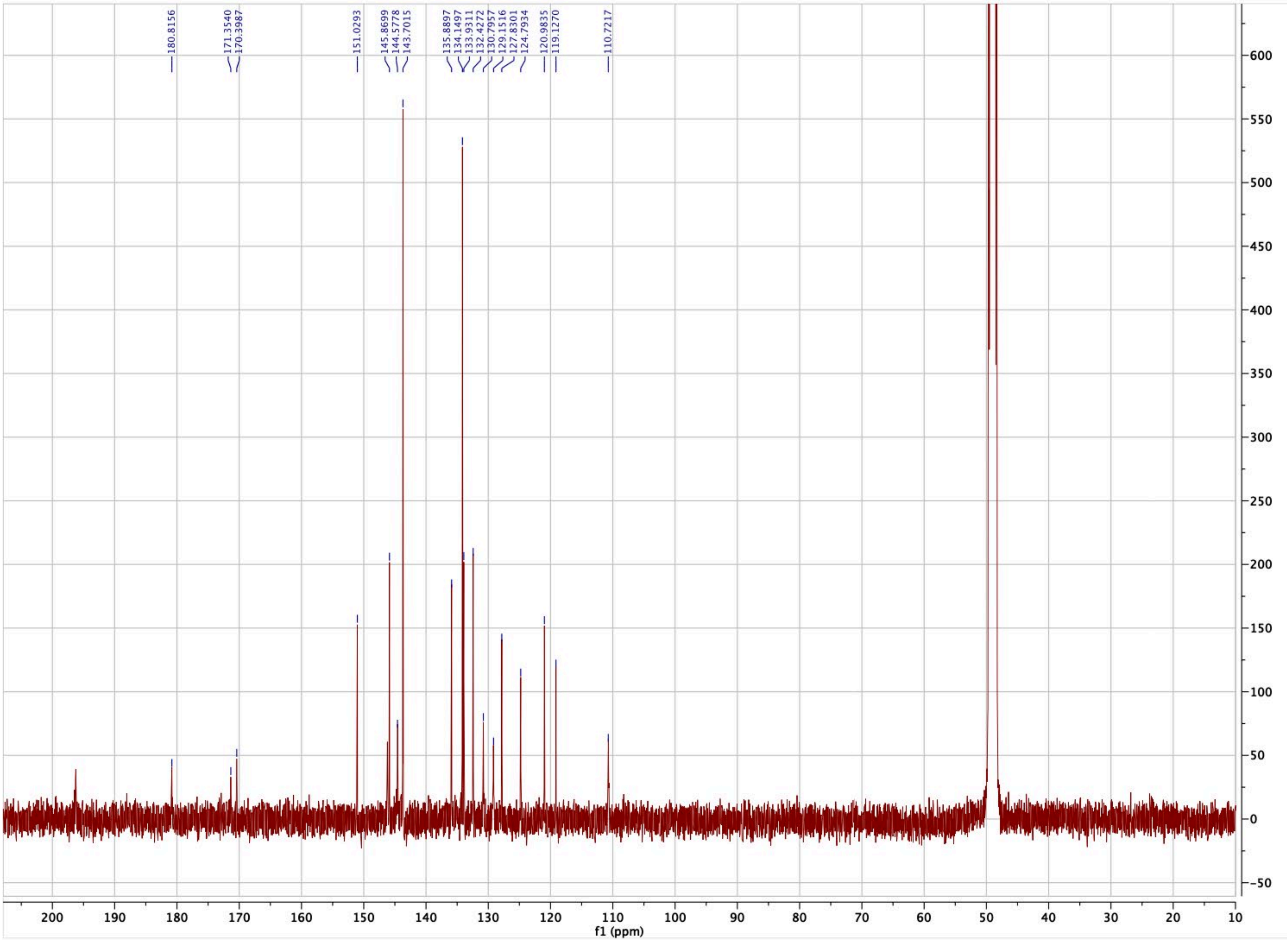

Figure S12. ^{13}C NMR Spectrum for compound **5g**

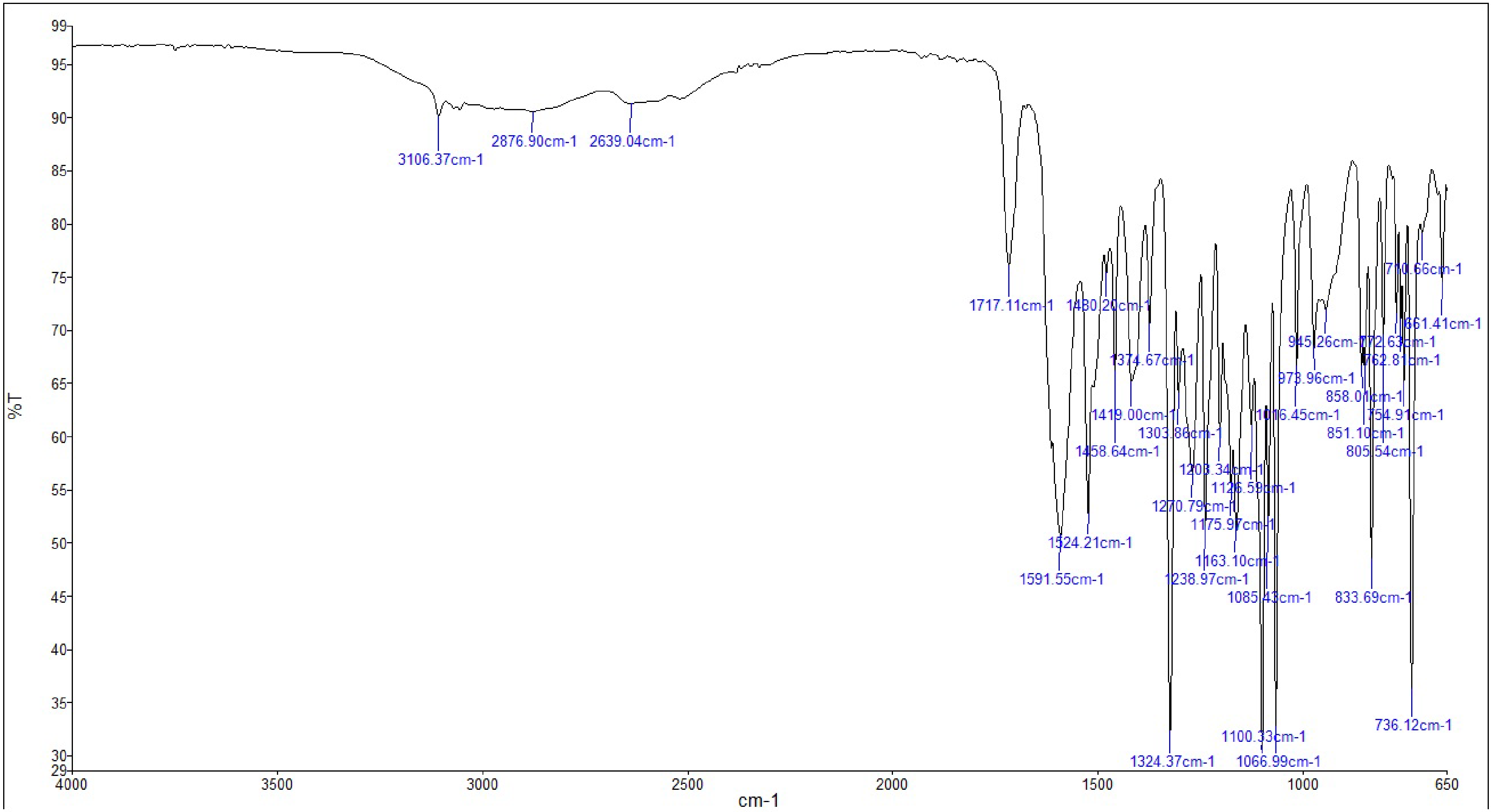

Figure S13. FTIR Spectrum for compound **5h**

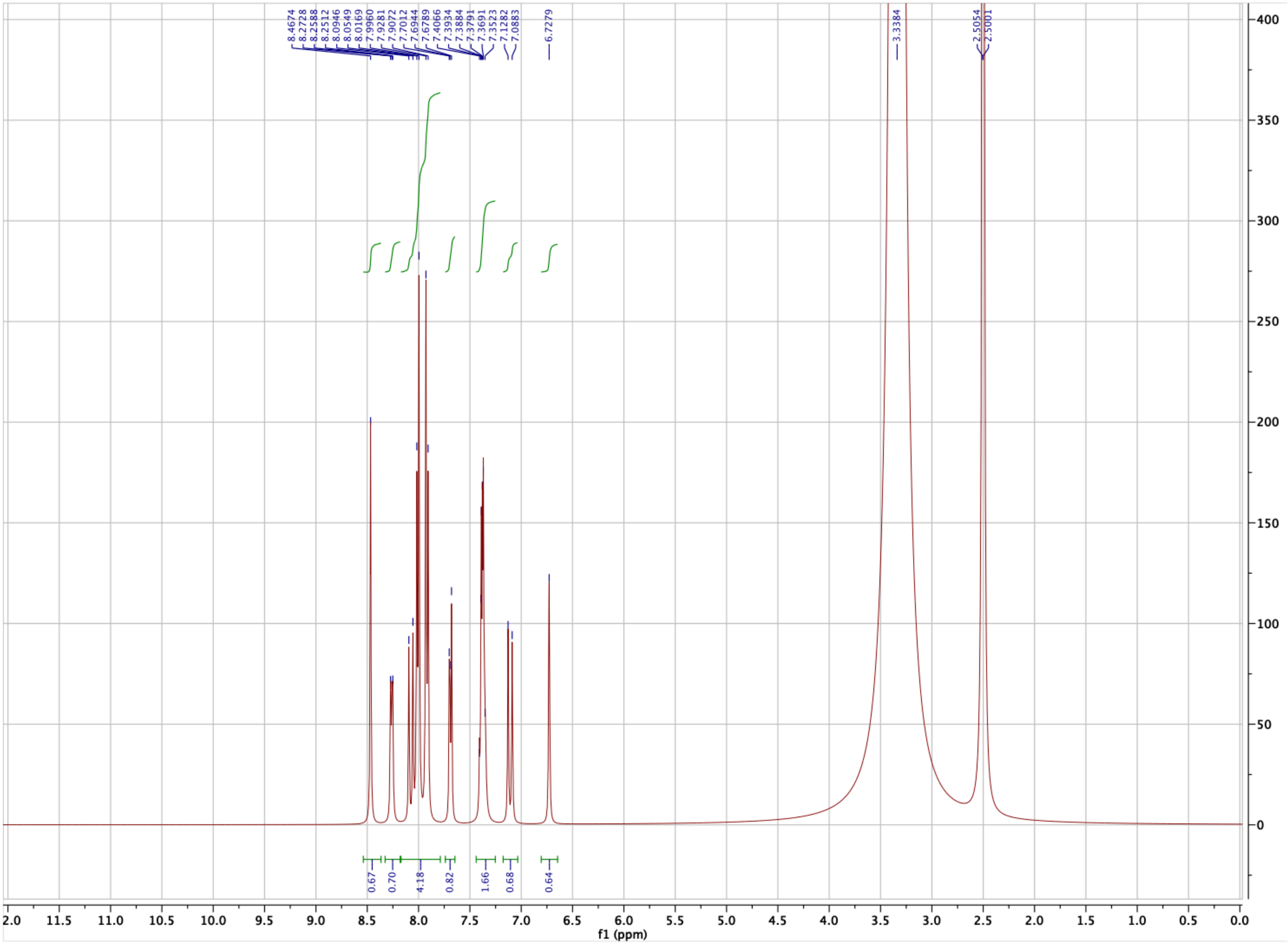

Figure S14. ^{1}H NMR Spectrum for compound **5h**

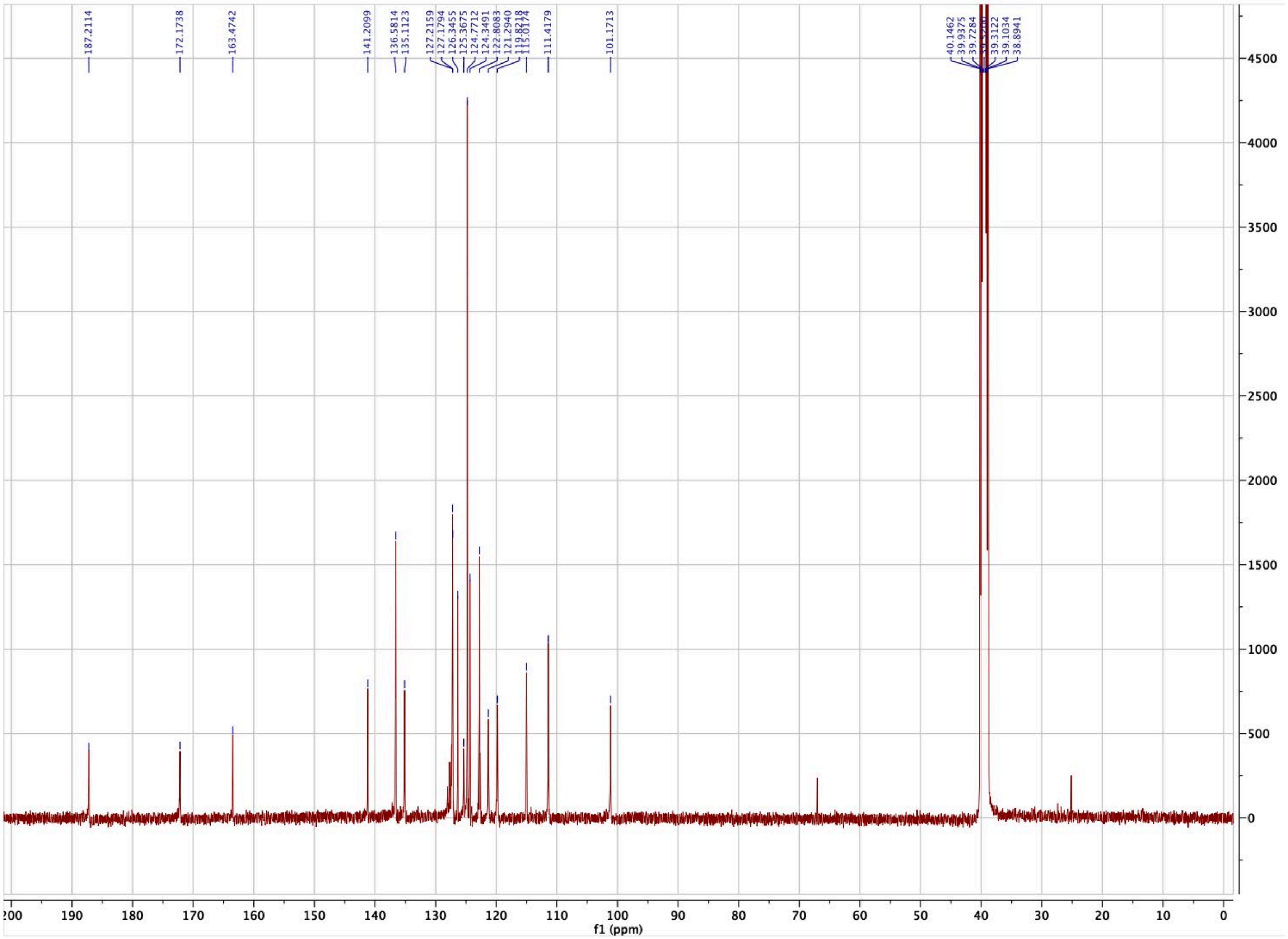

Figure S15. ^{13}C NMR Spectrum for compound **5h**

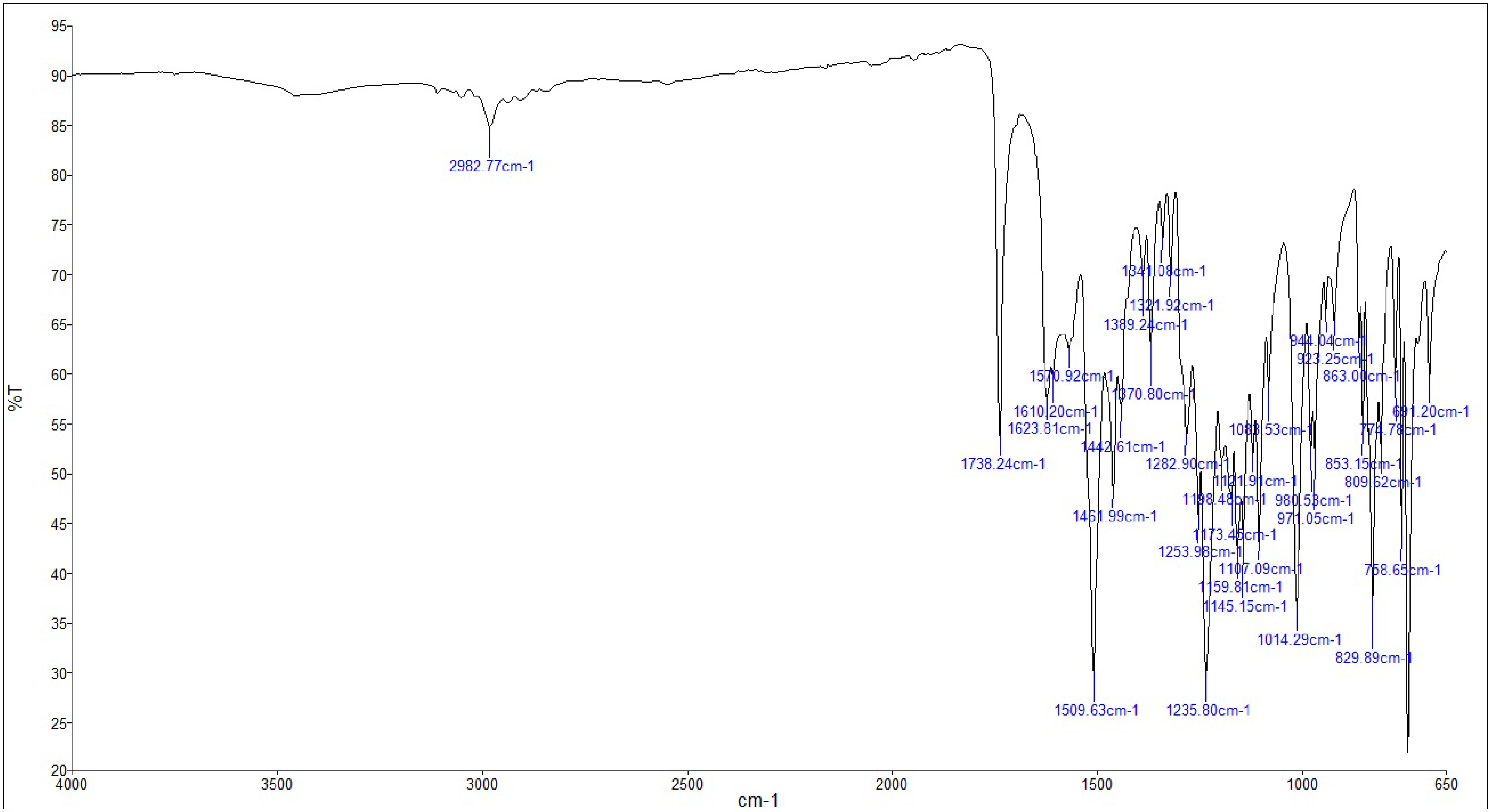

Figure S16. FTIR Spectrum for compound **6a**

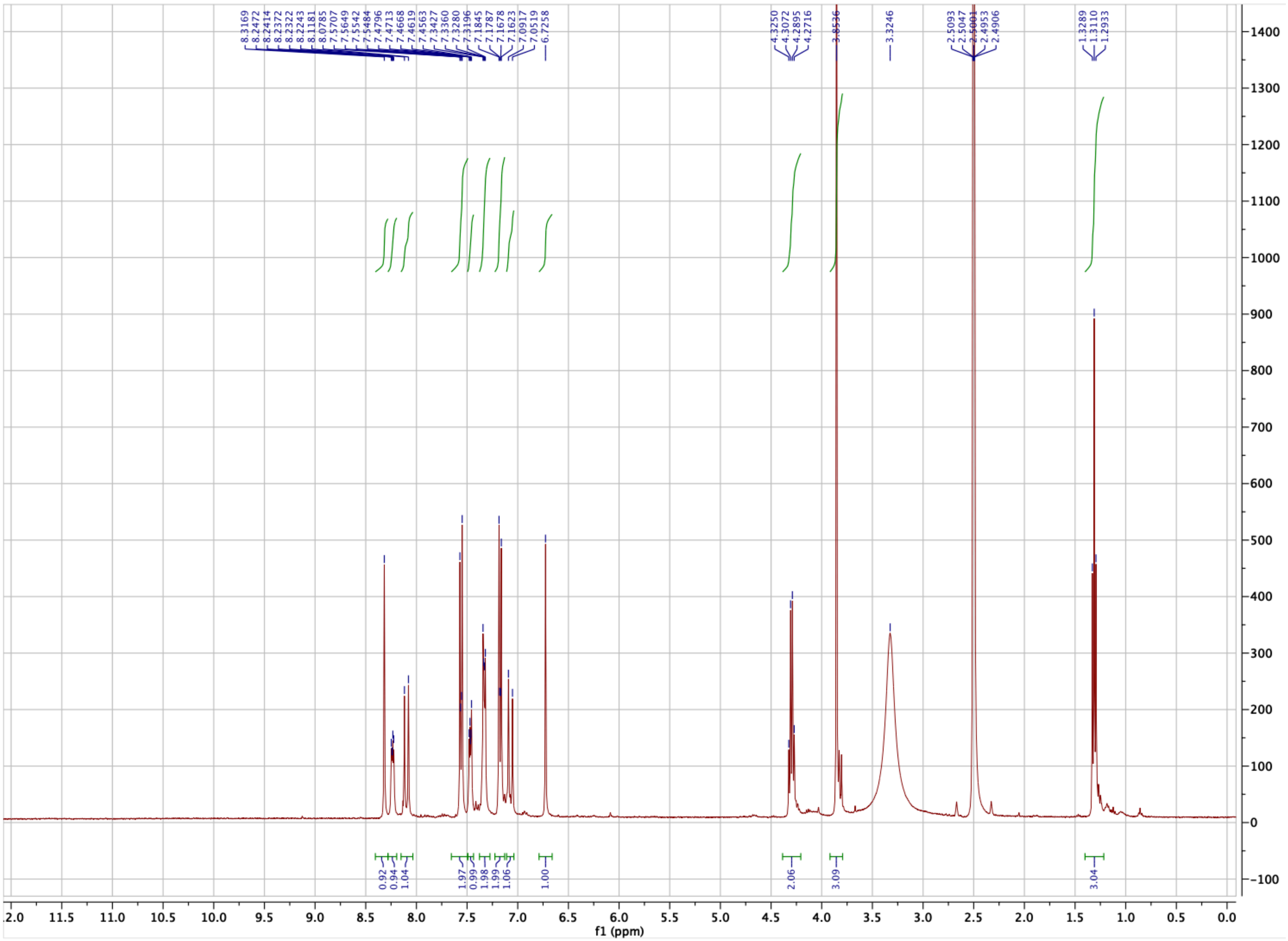

Figure S17. ^{1}H NMR Spectrum for compound **6a**

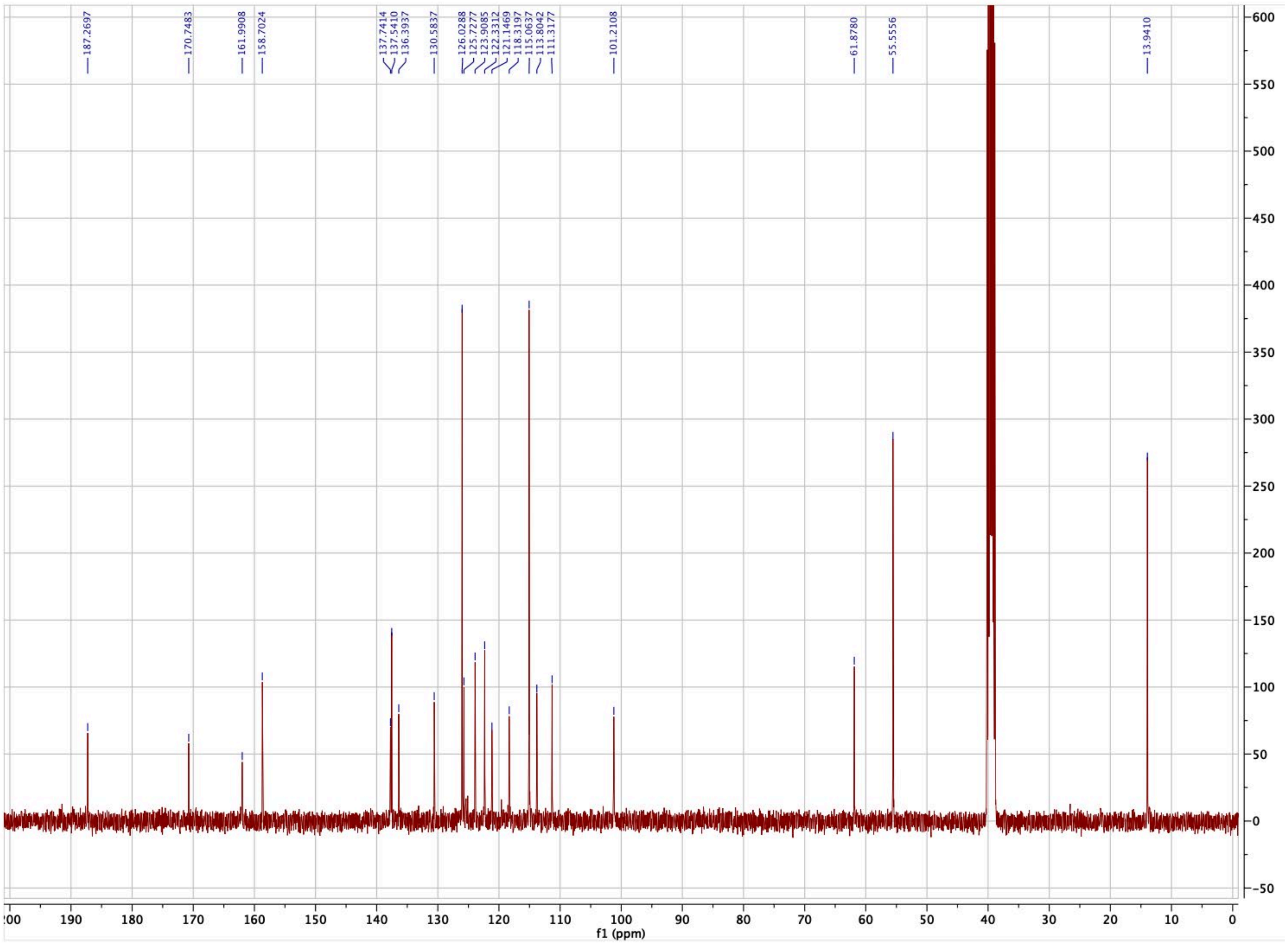

Figure S18. ^{13}C NMR Spectrum for compound **6a**

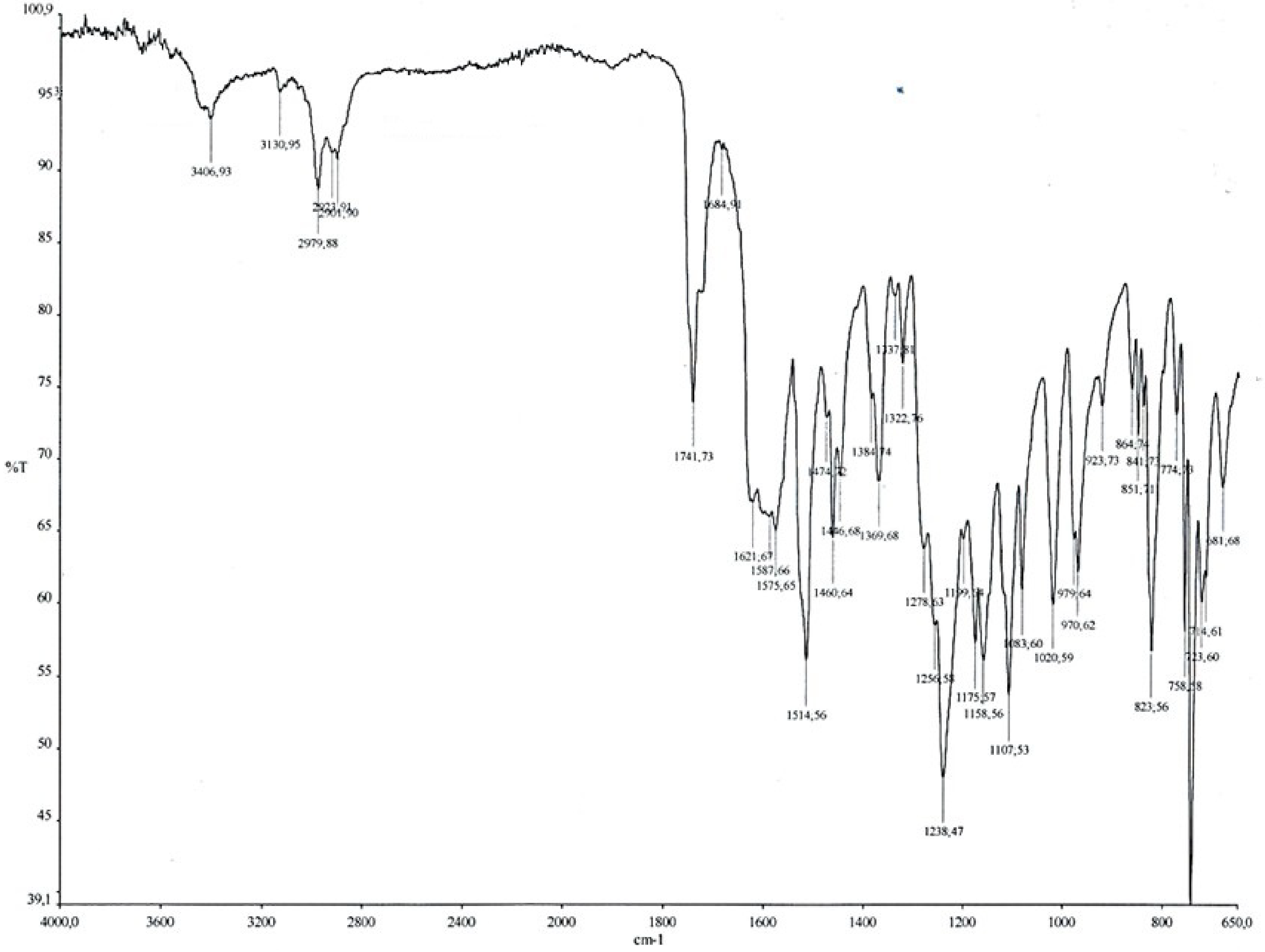

Figure S19. FTIR Spectrum for compound **6b**

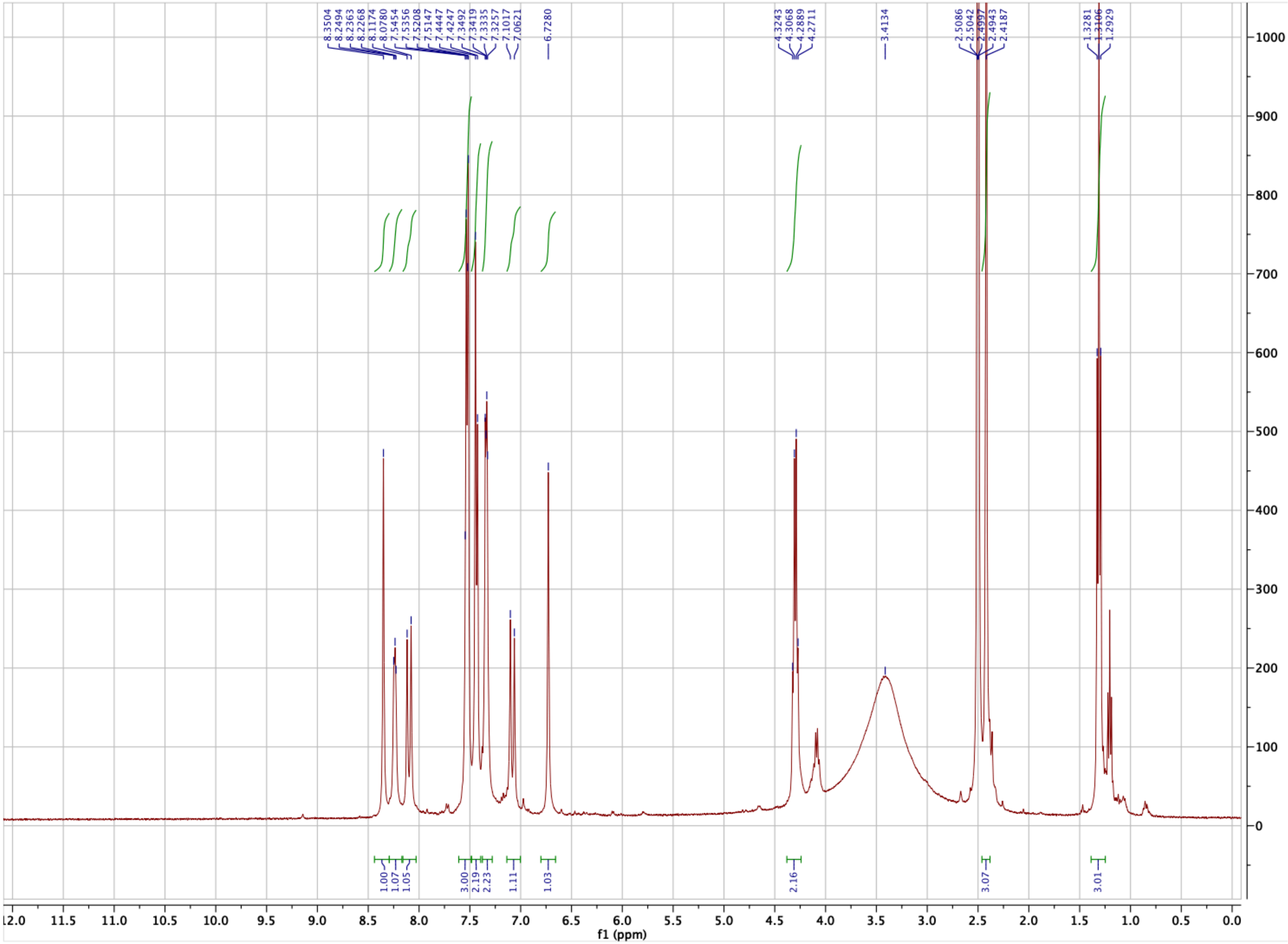

Figure S20. ^{1}H NMR Spectrum for compound **6b**

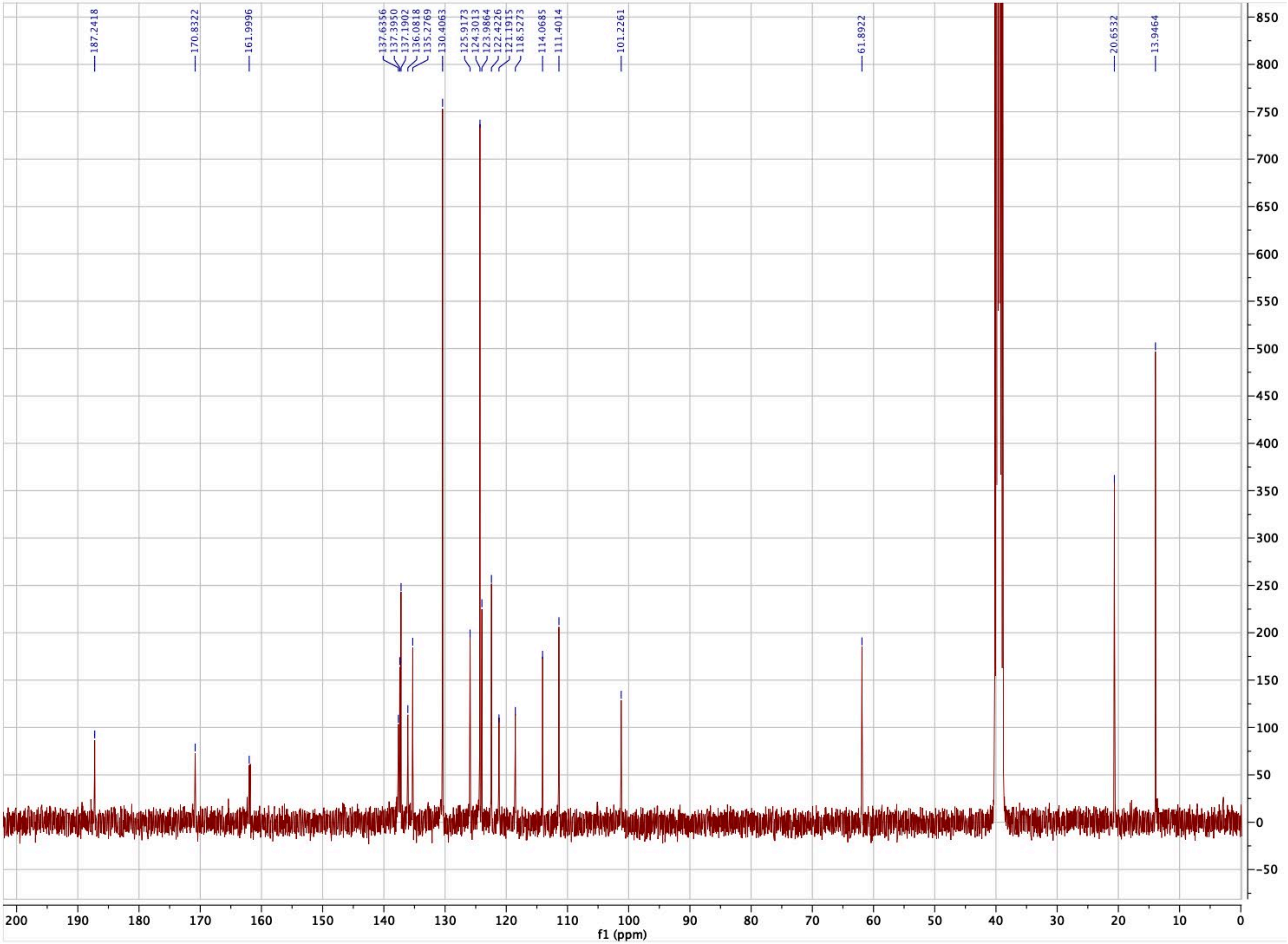

Figure S21. ^{13}C NMR Spectrum for compound **6b**

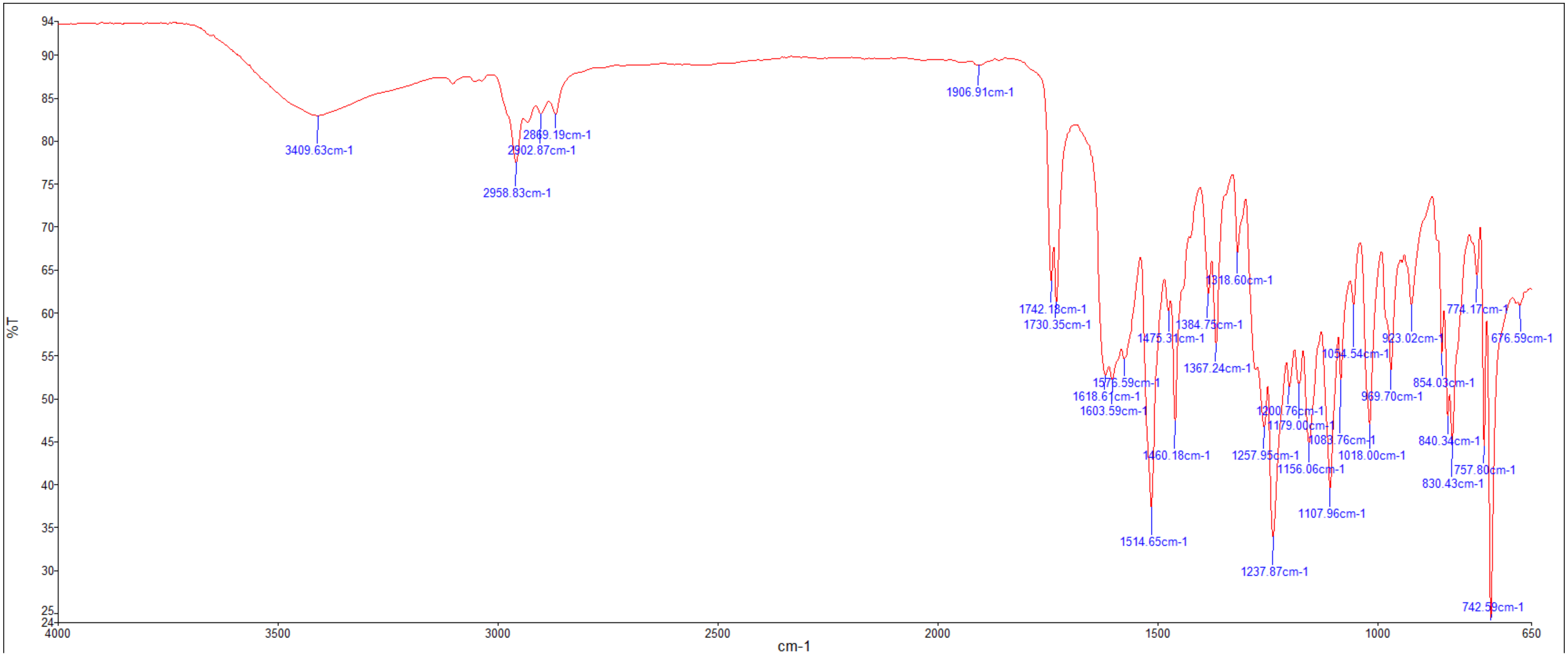

Figure S22. FTIR Spectrum for compound **6c**

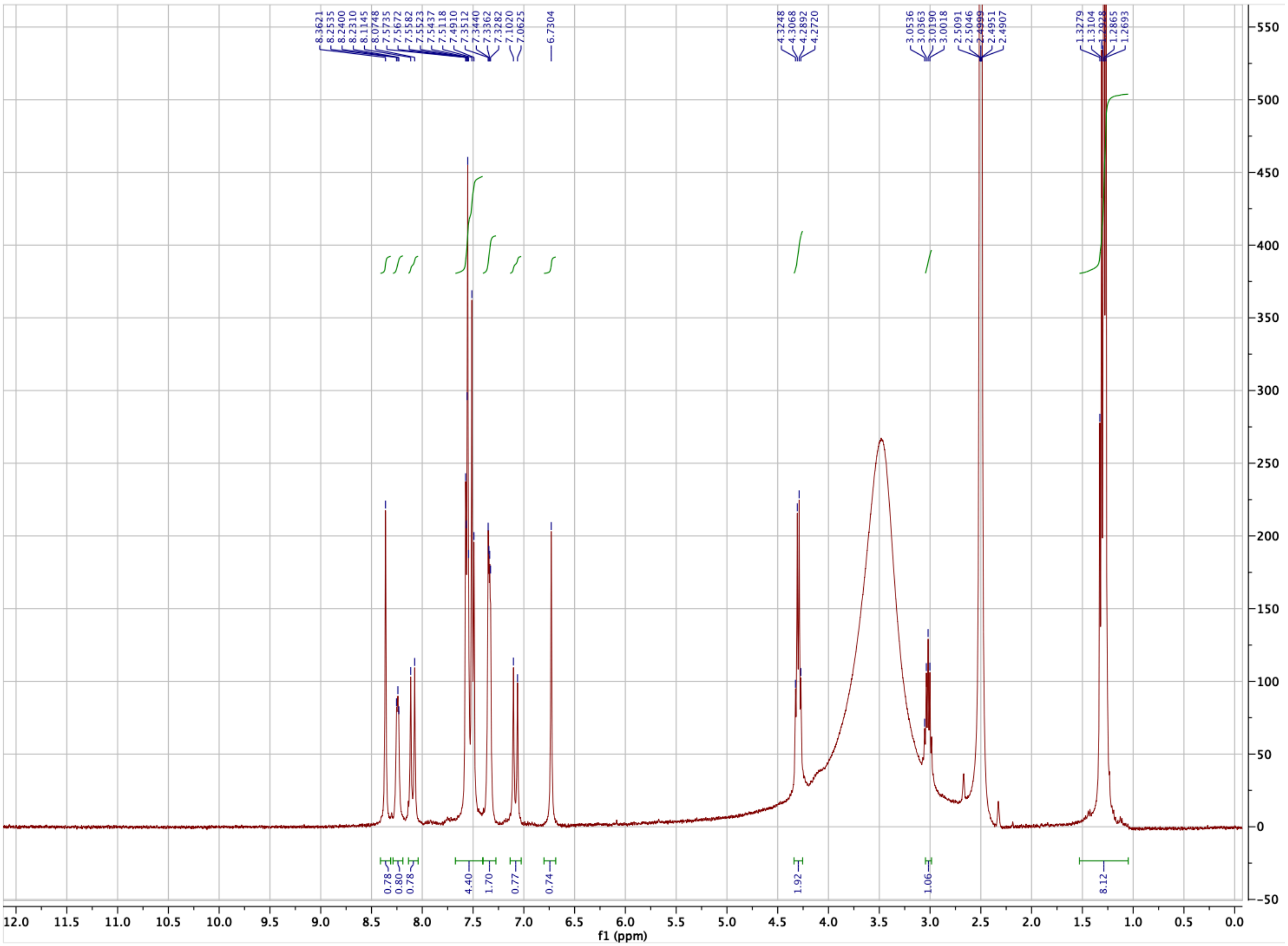

Figure S23. ^{1}H NMR Spectrum for compound **6c**

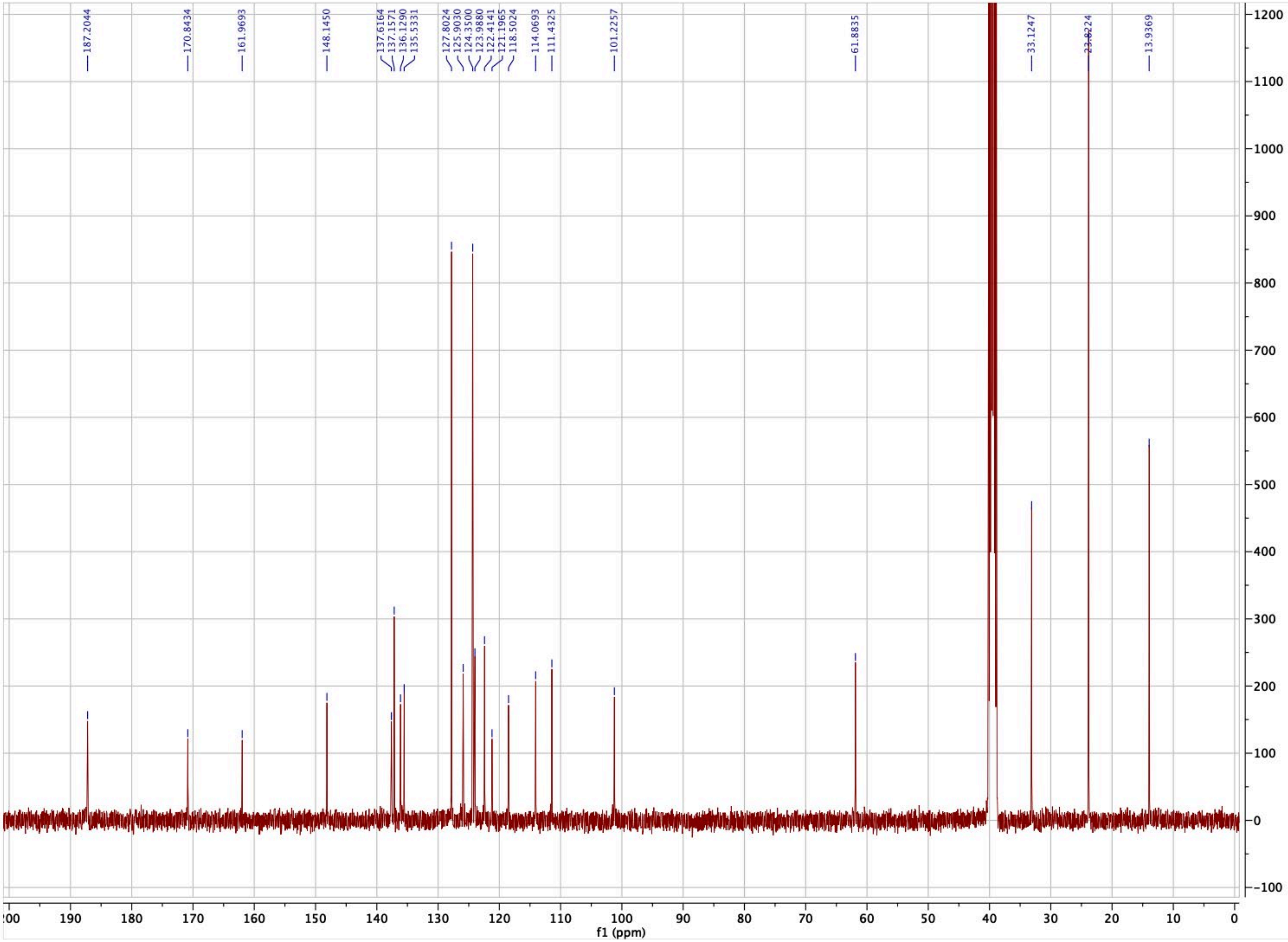

Figure S24. ^{13}C NMR Spectrum for compound **6c**

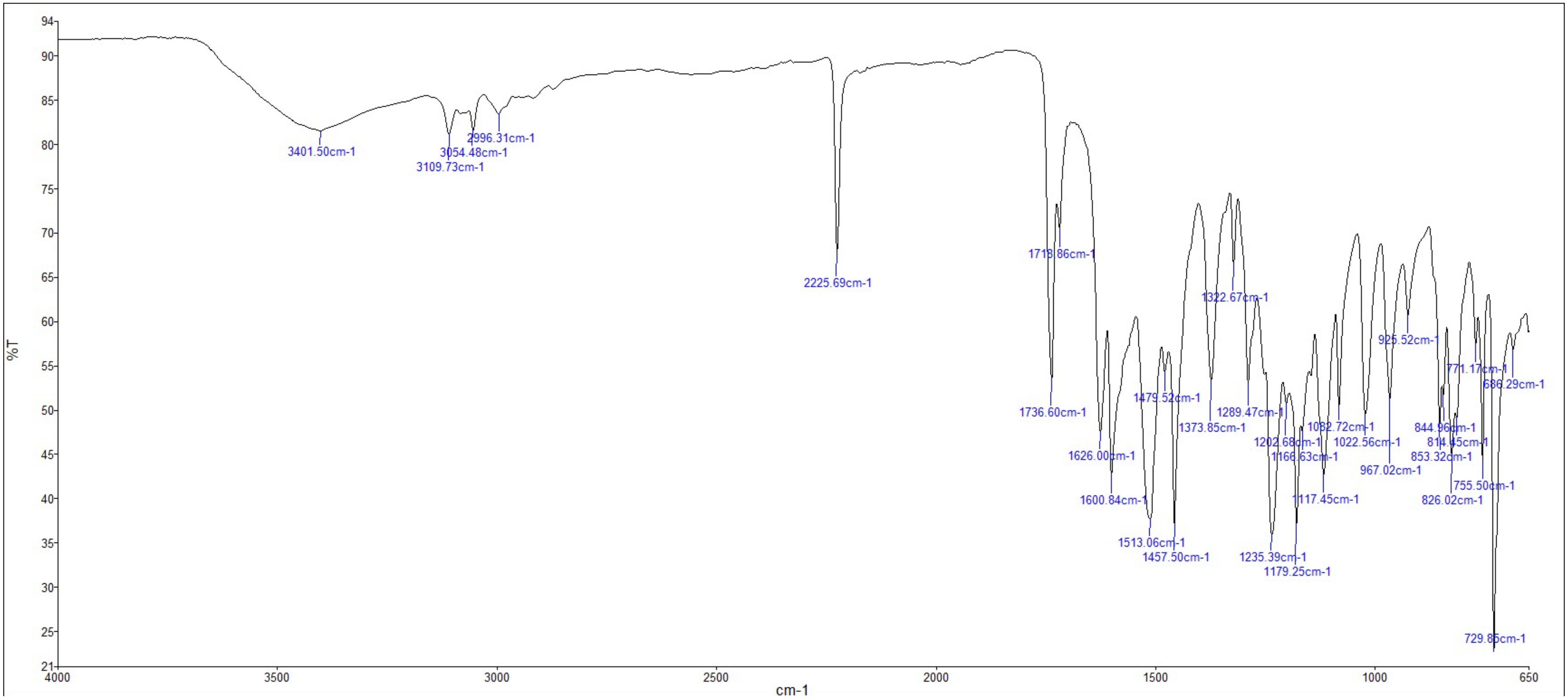

Figure S25. FTIR Spectrum for compound **6g**

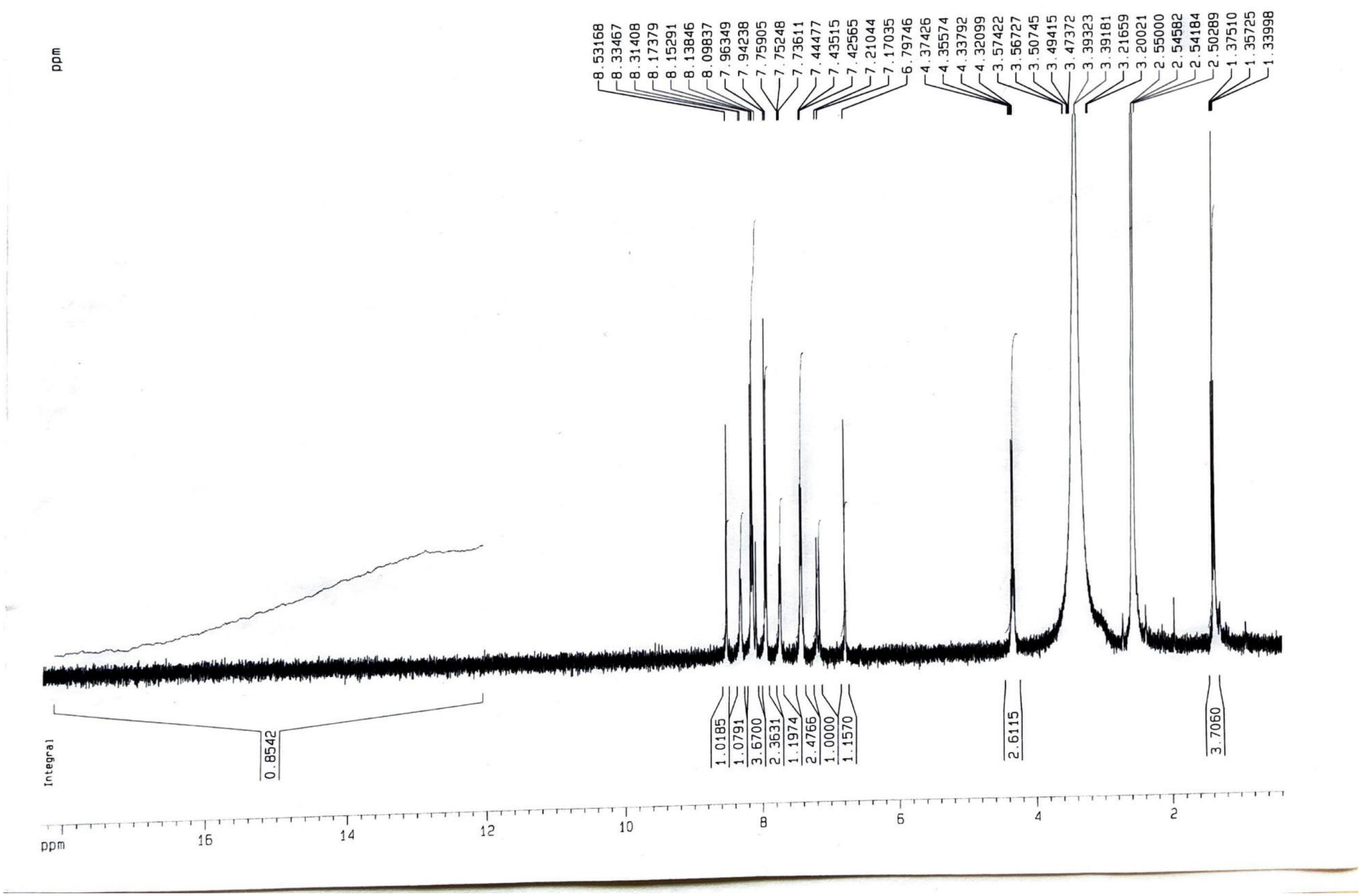

Figure S26. ^{1}H NMR Spectrum for compound **6g**

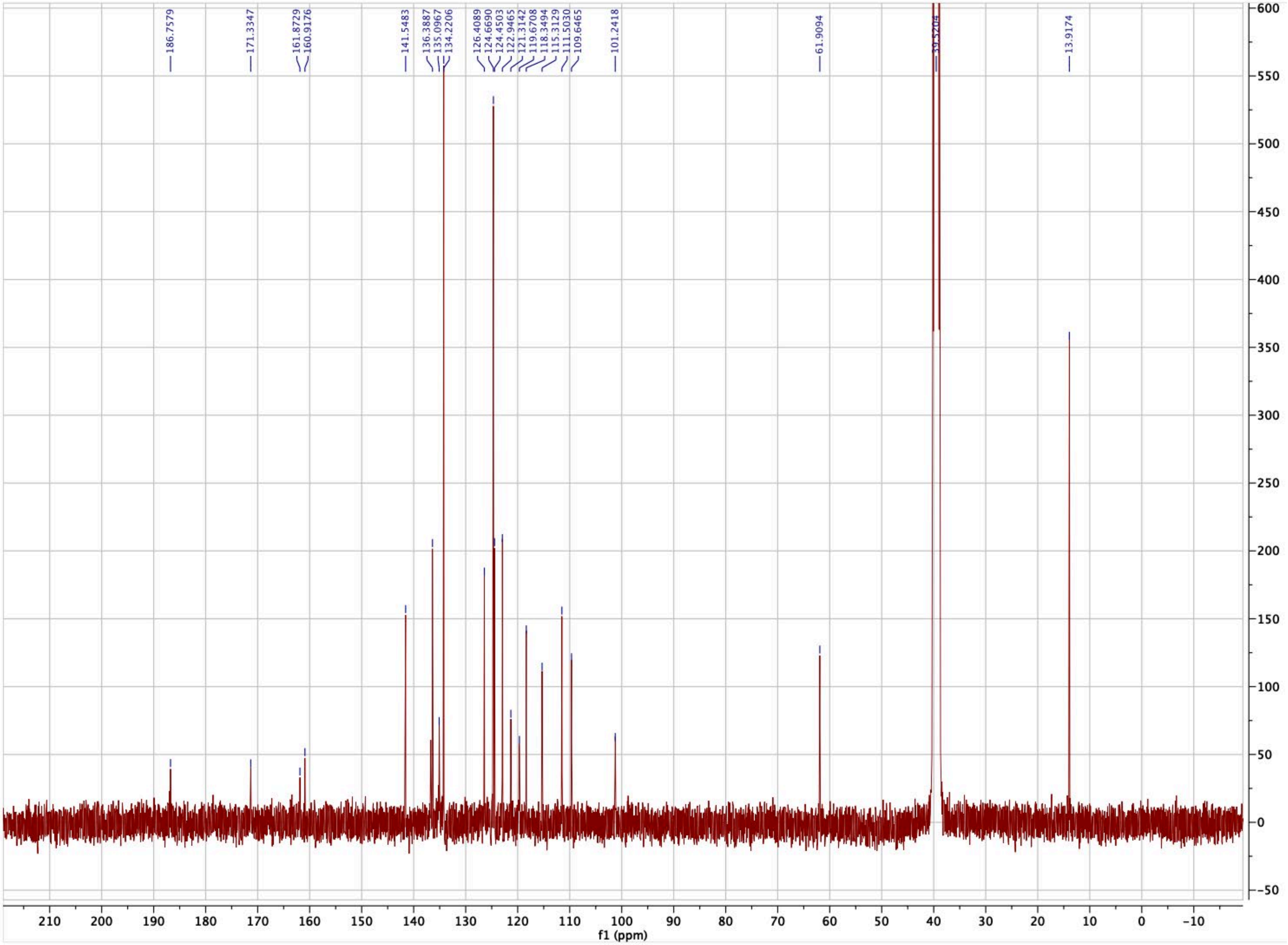

Figure S27. ^{13}C NMR Spectrum for compound **6g**

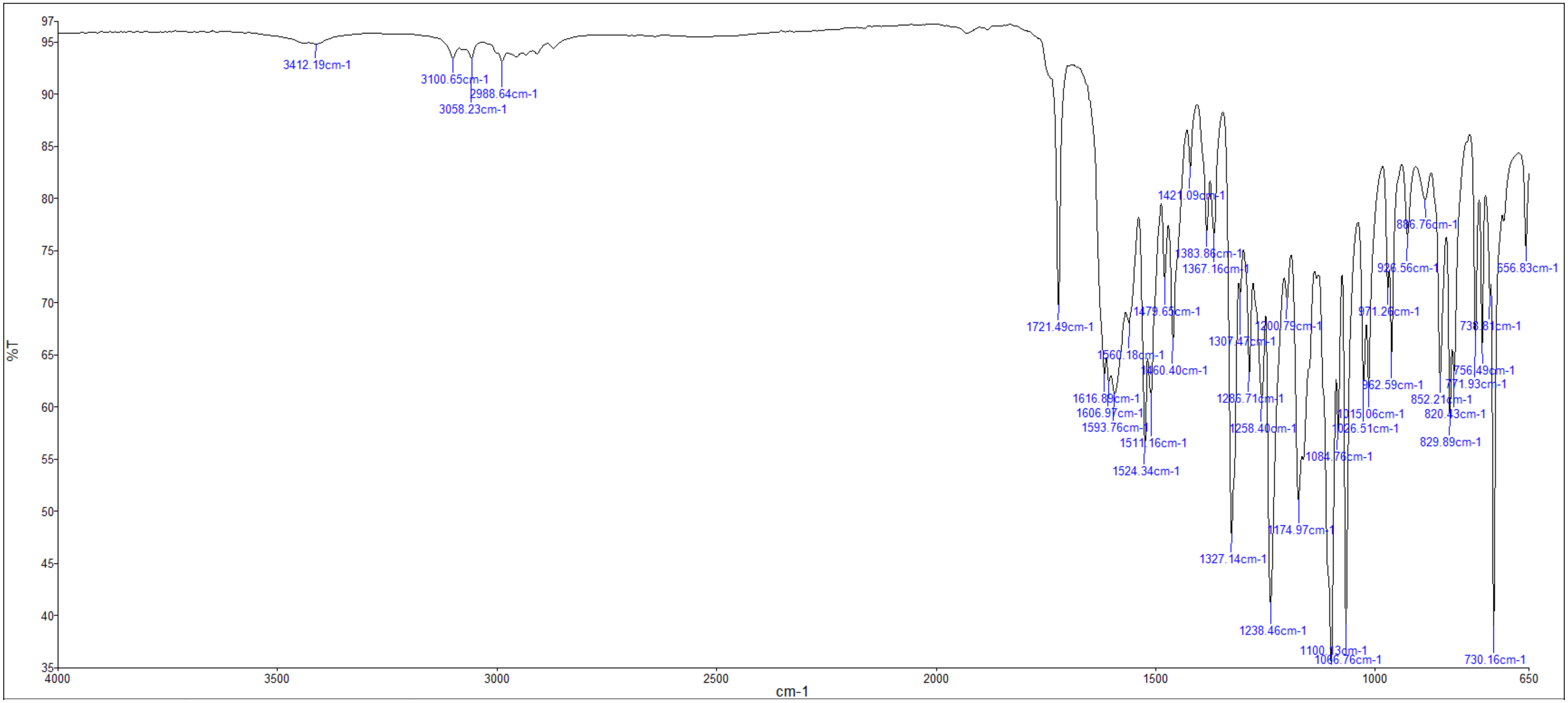

Figure S28. FTIR Spectrum for compound **6h**

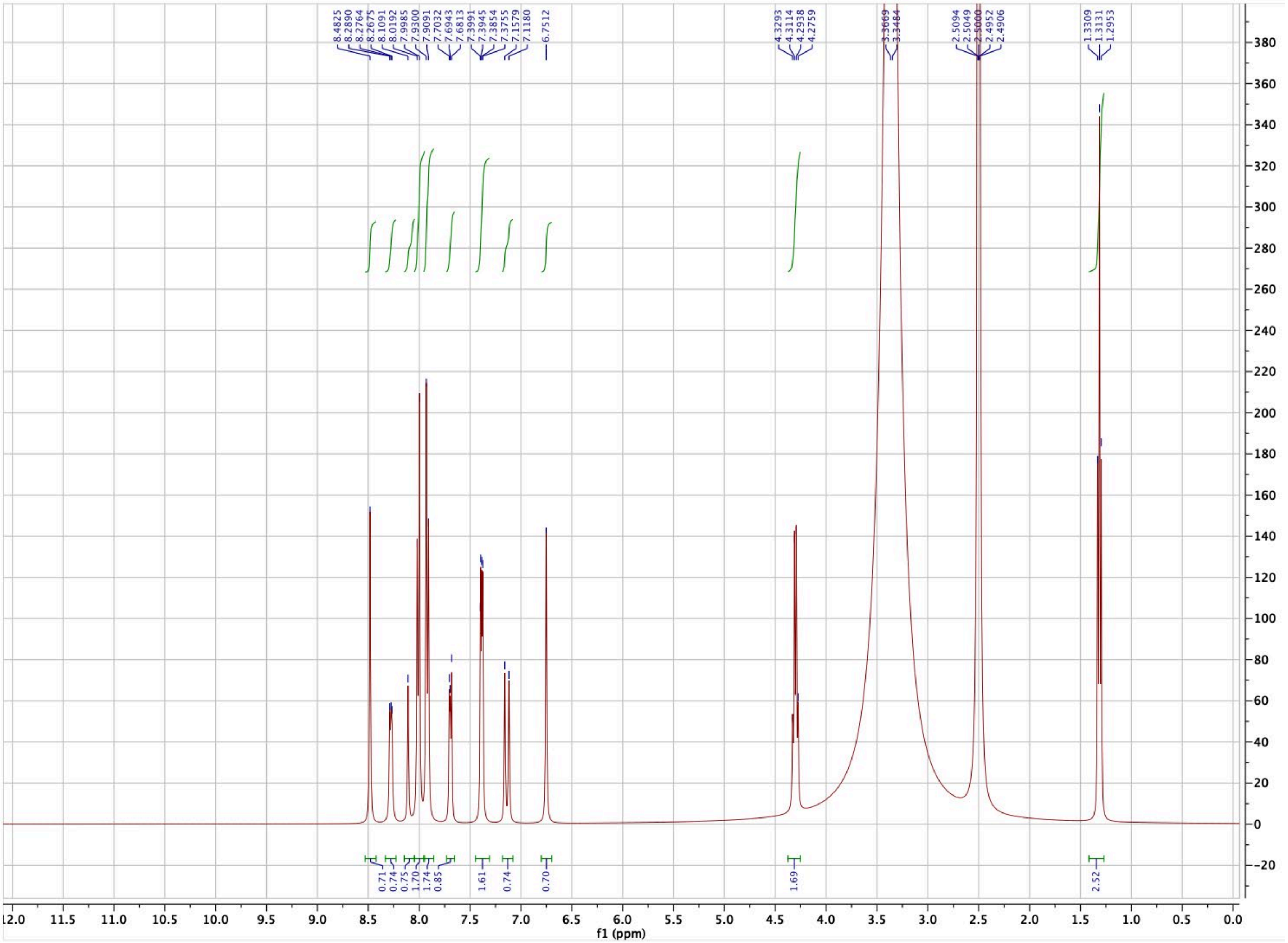

Figure S29. ^{1}H NMR Spectrum for compound **6h**

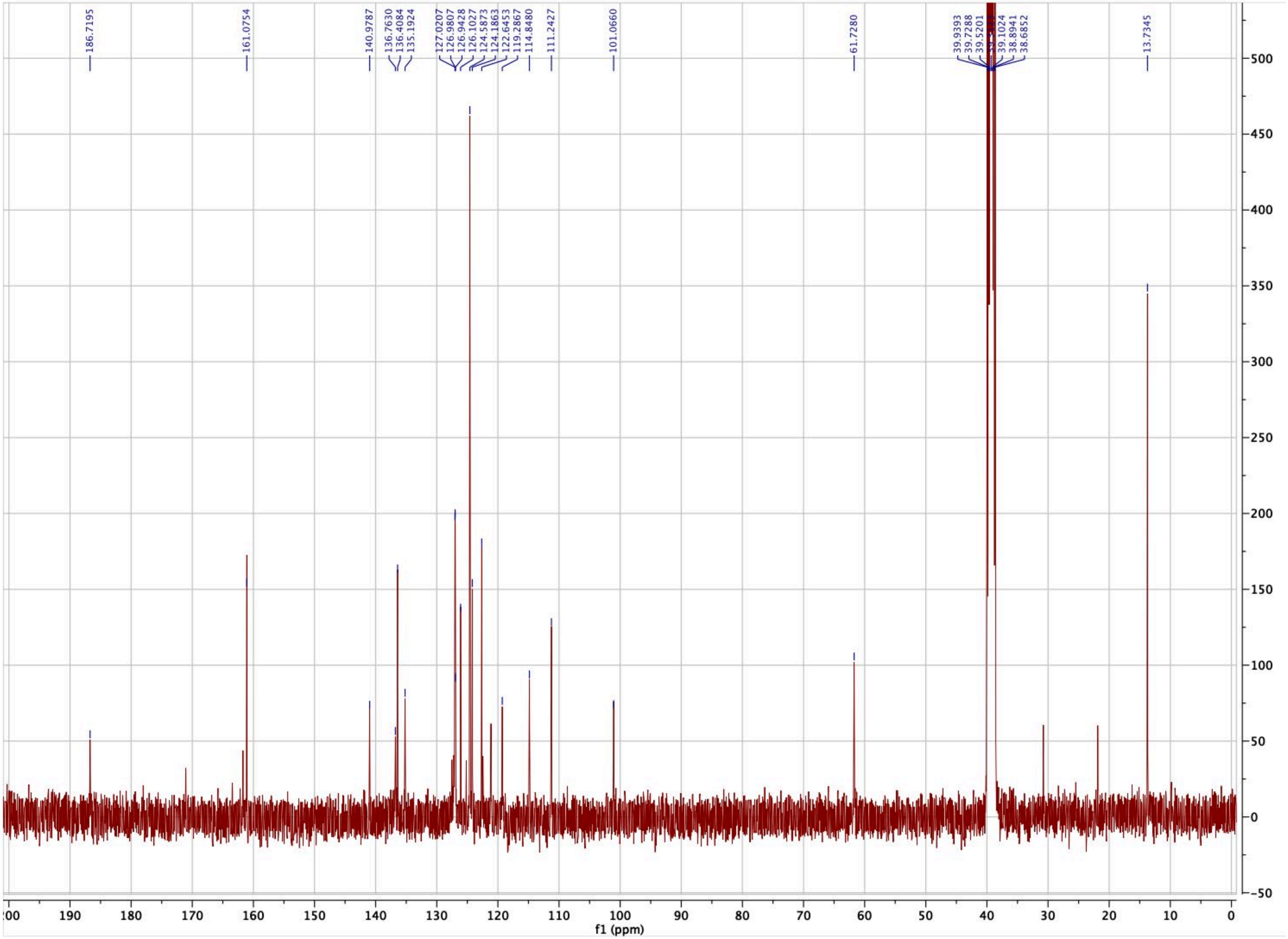

Figure S30. ^{13}C NMR Spectrum for compound **6h**

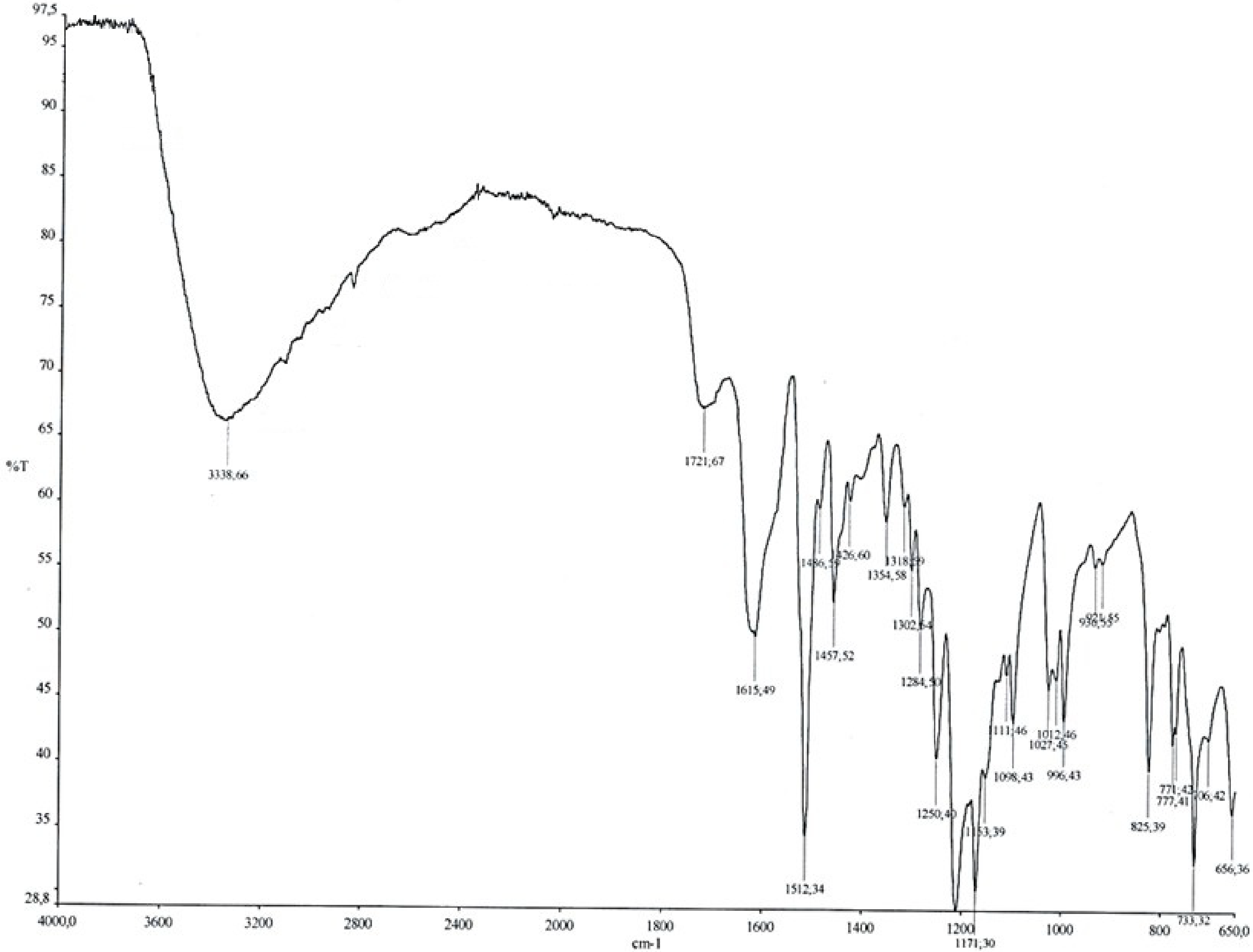

Figure S31. FTIR Spectrum for compound **7a**

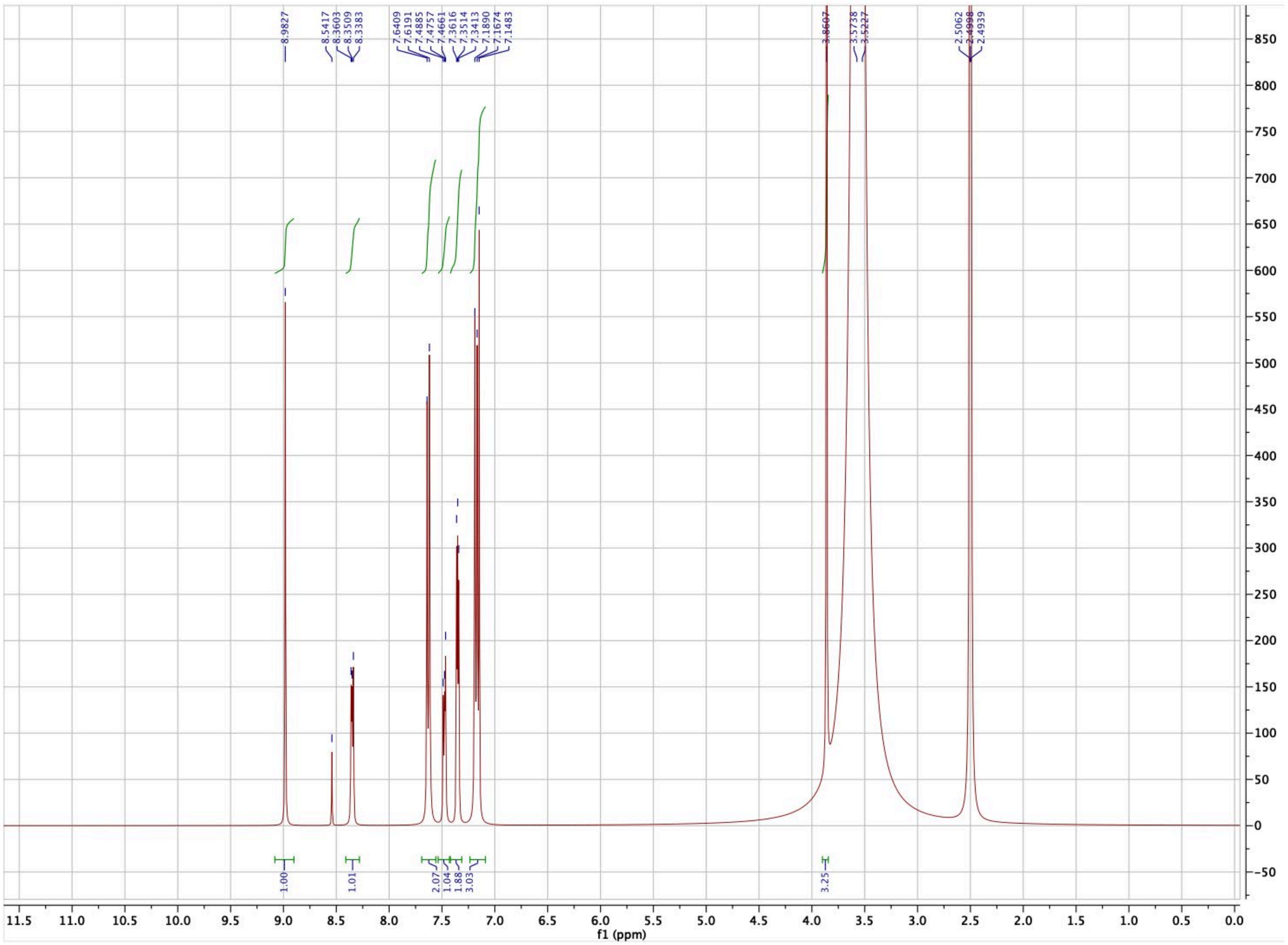

Figure S32. ^{1}H NMR Spectrum for compound **7a**

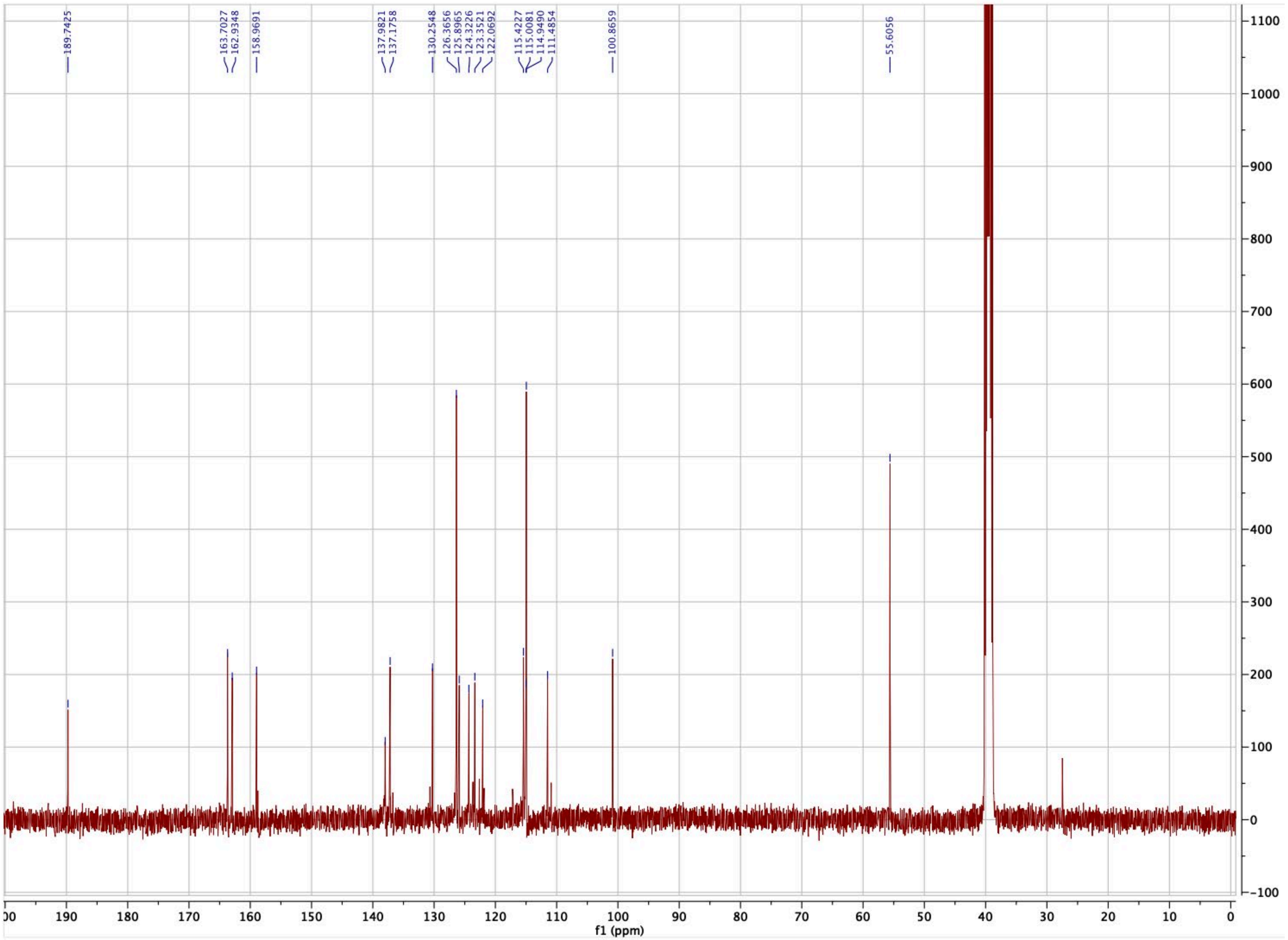

Figure S33. ^{13}C NMR Spectrum for compound **7a**

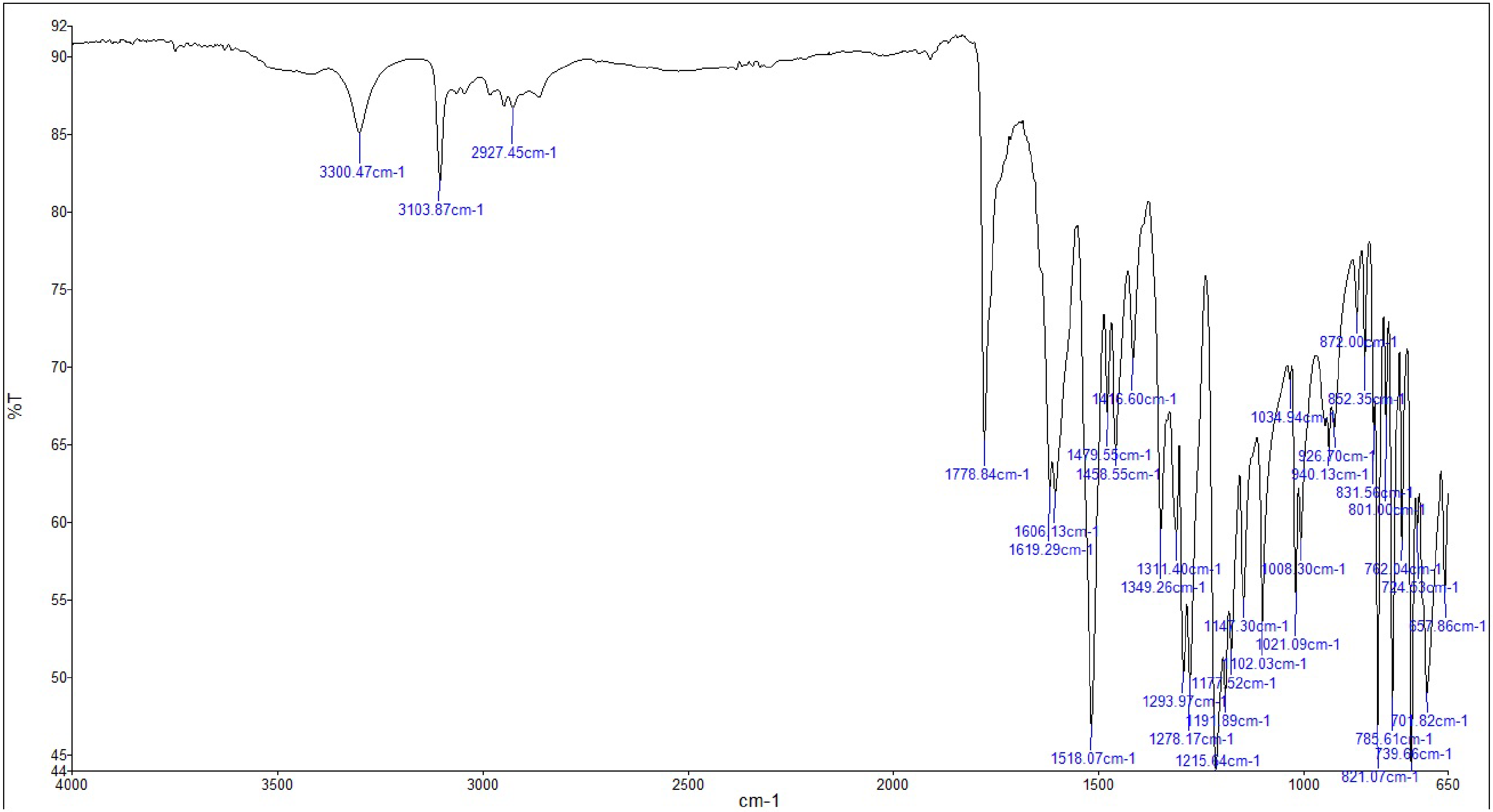

Figure S34. FTIR Spectrum for compound **7b**

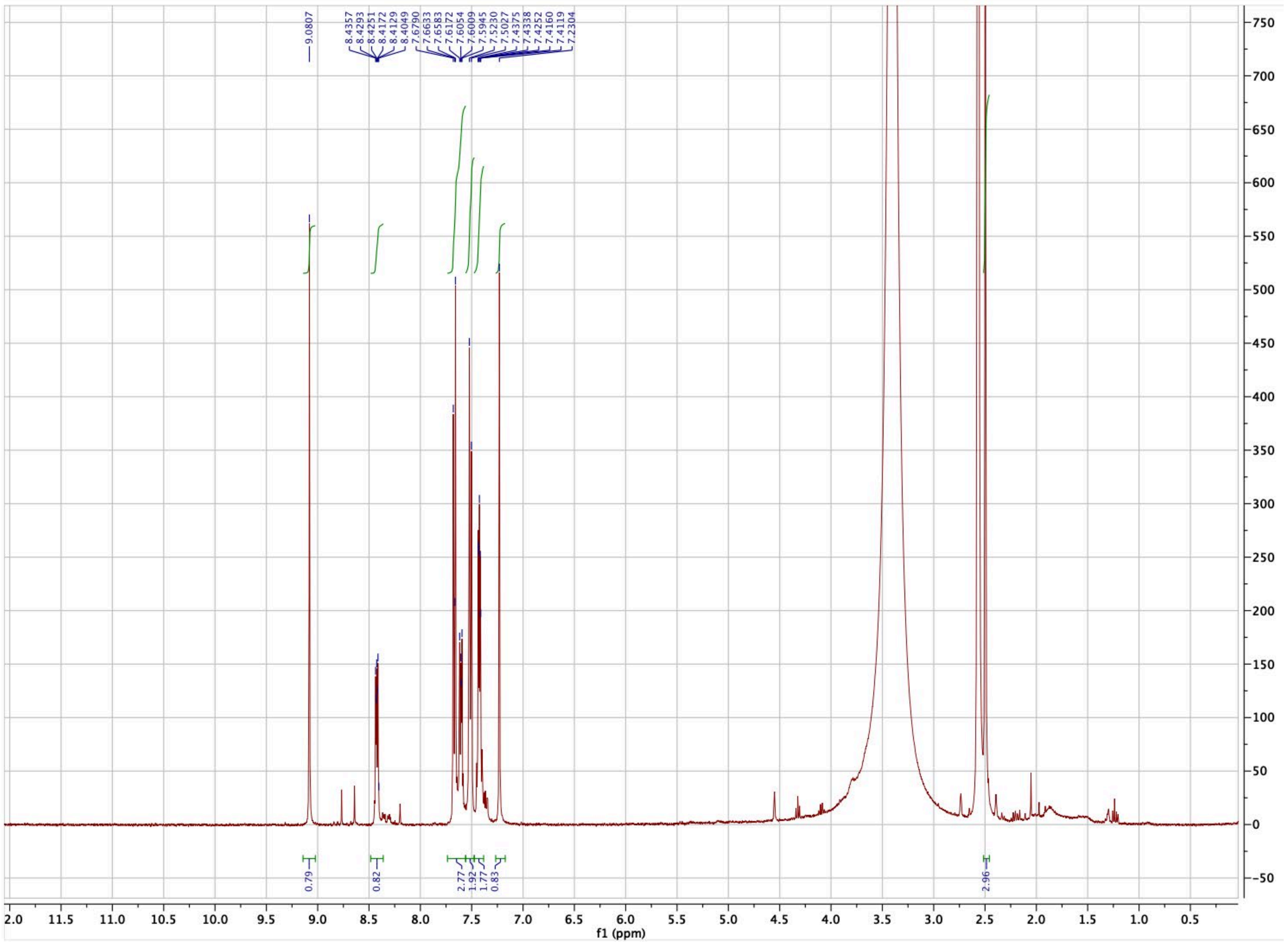

Figure S35. ^{1}H NMR Spectrum for compound **7b**

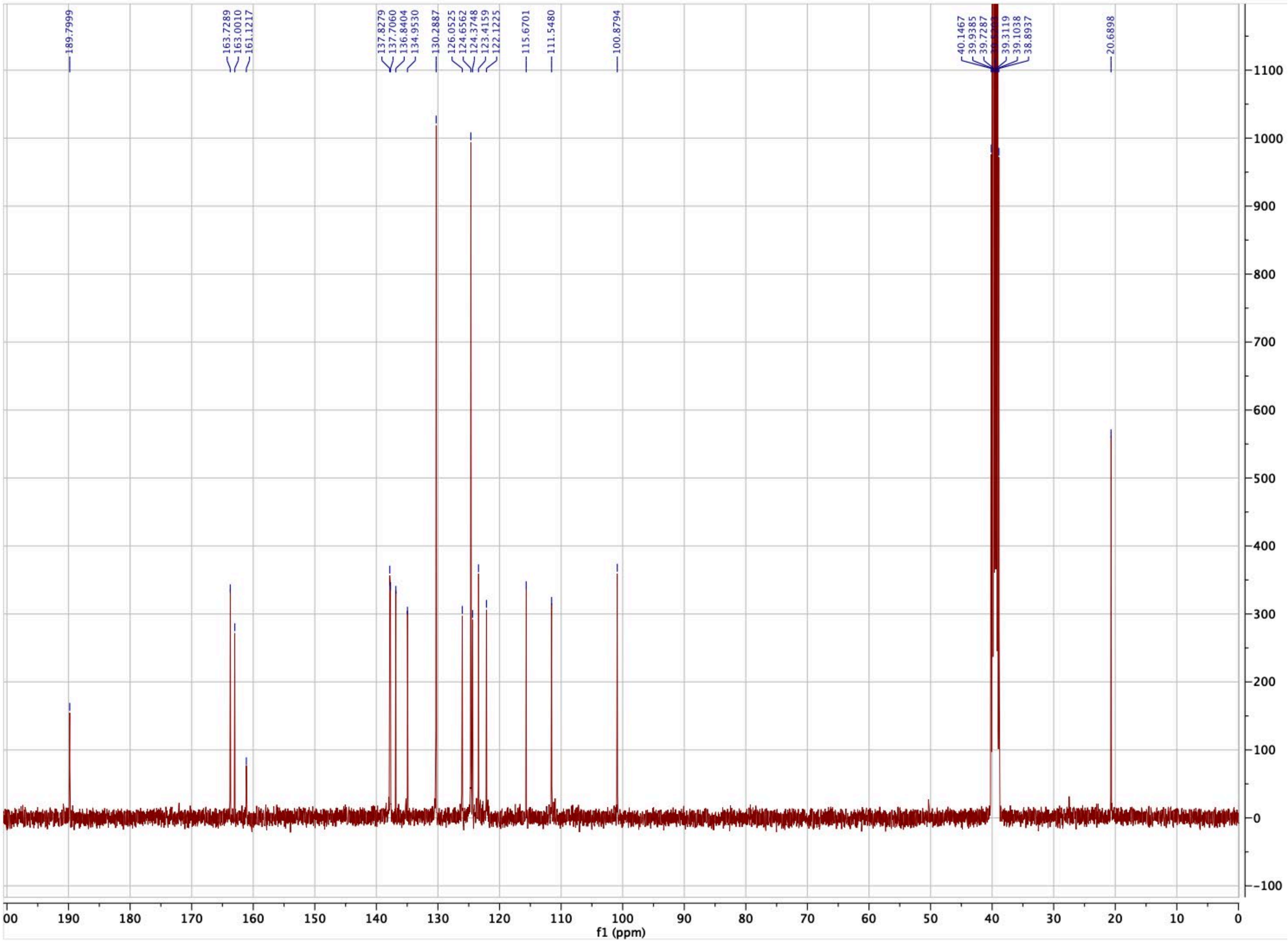

Figure S36. ^{13}C NMR Spectrum for compound **7b**

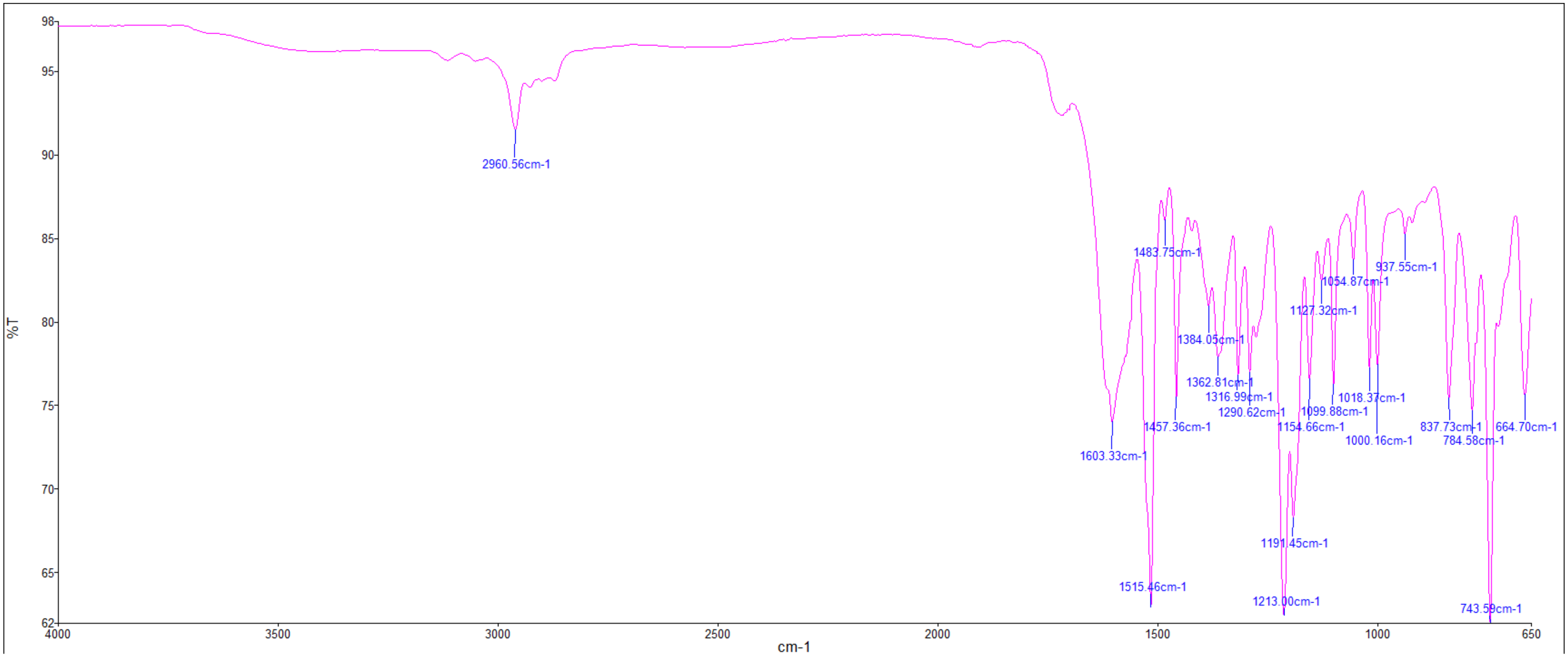

Figure S37. FTIR Spectrum for compound **7c**

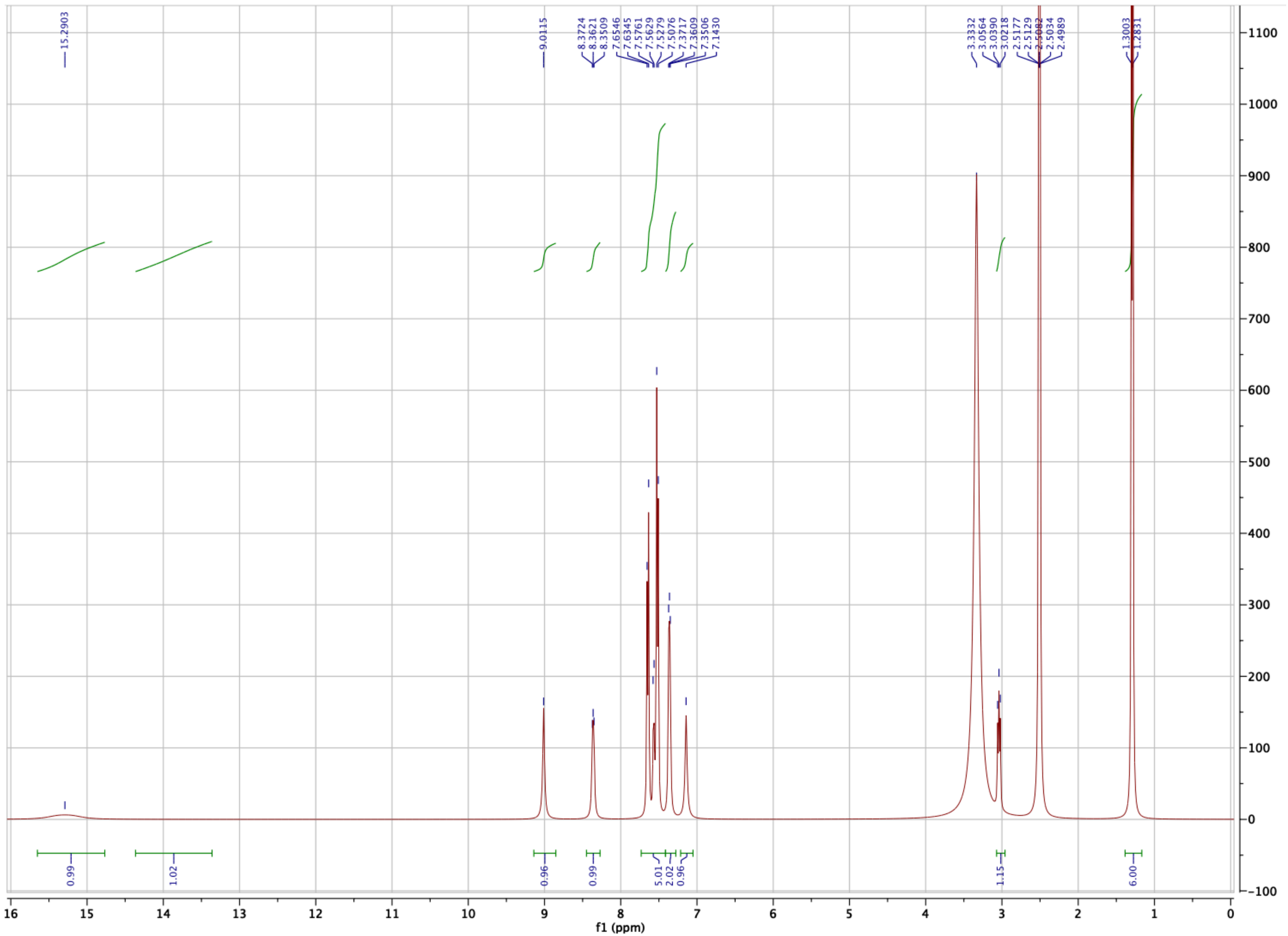

Figure S38. ^{1}H NMR Spectrum for compound **7c**

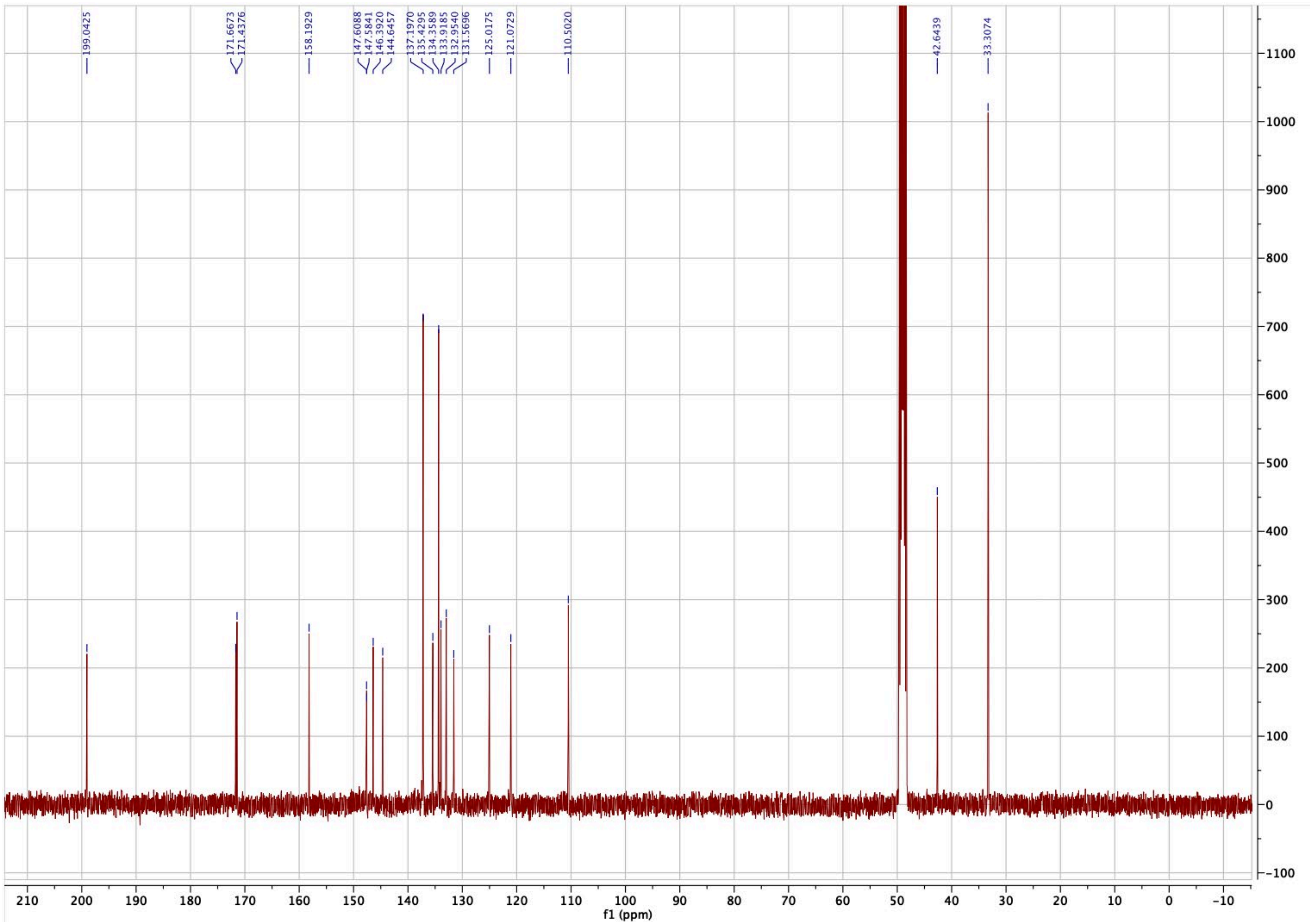

Figure S39. ^{13}C NMR Spectrum for compound **7c**

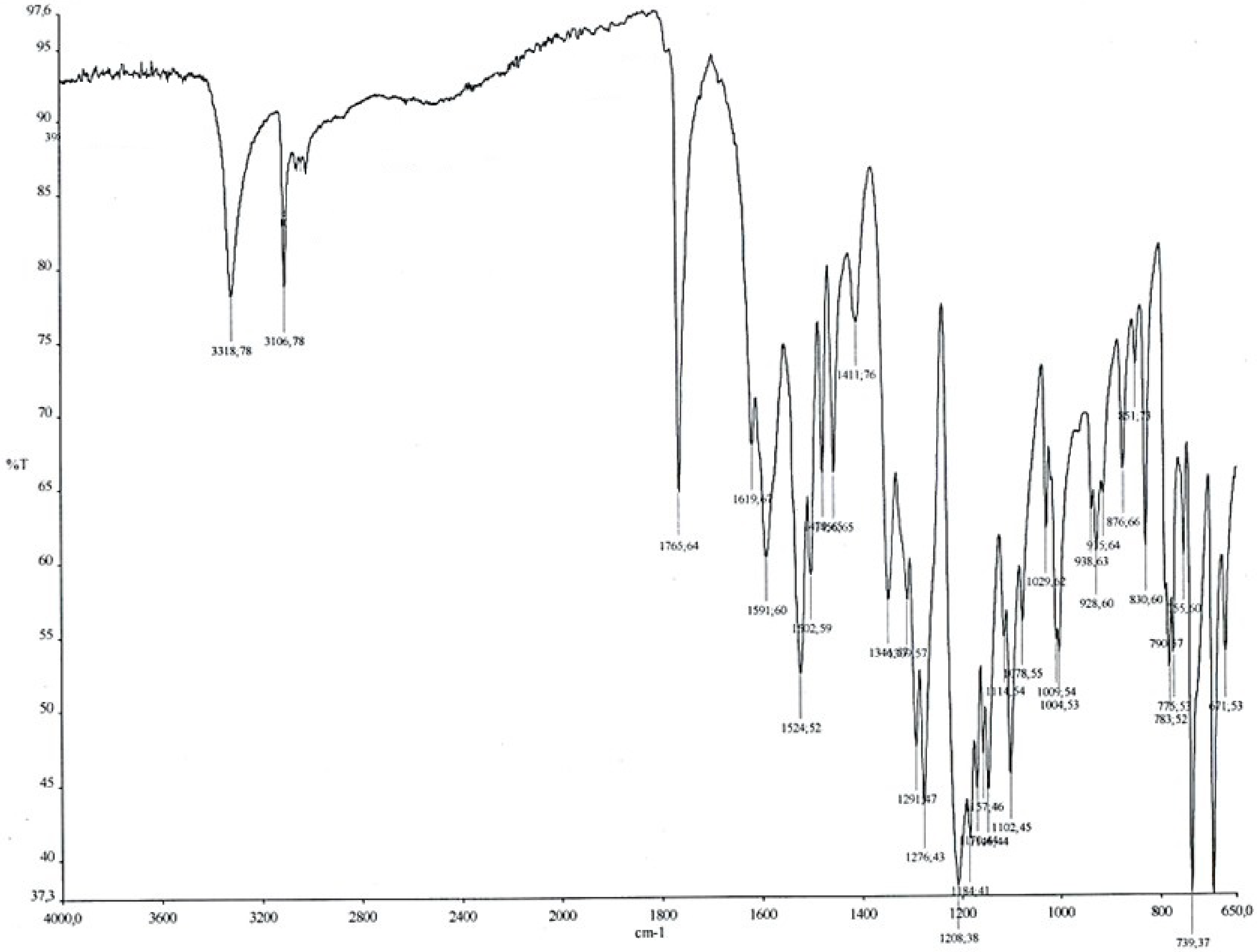

Figure S40. FTIR Spectrum for compound **7d**

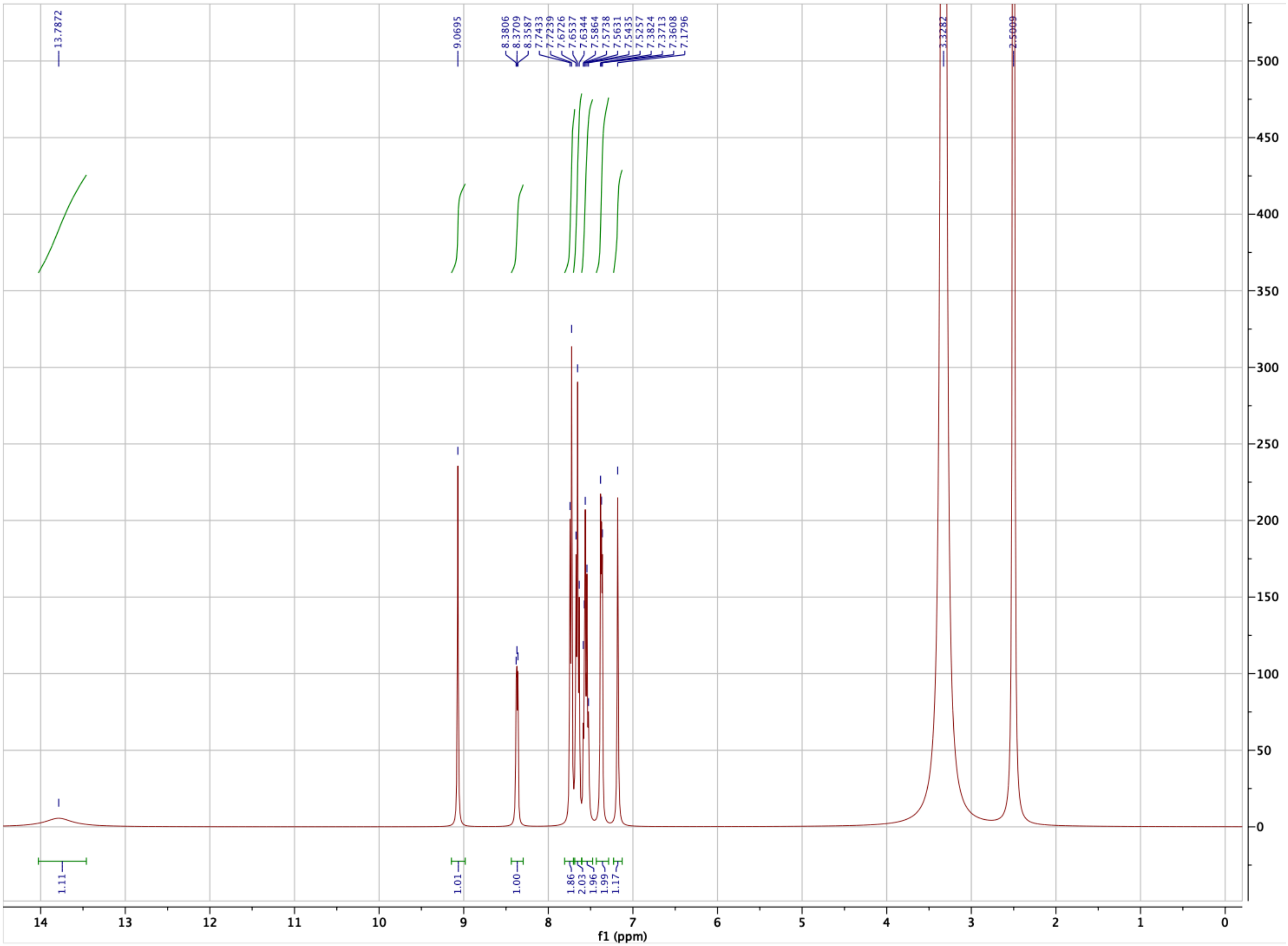

Figure S41. ^{1}H NMR Spectrum for compound **7d**

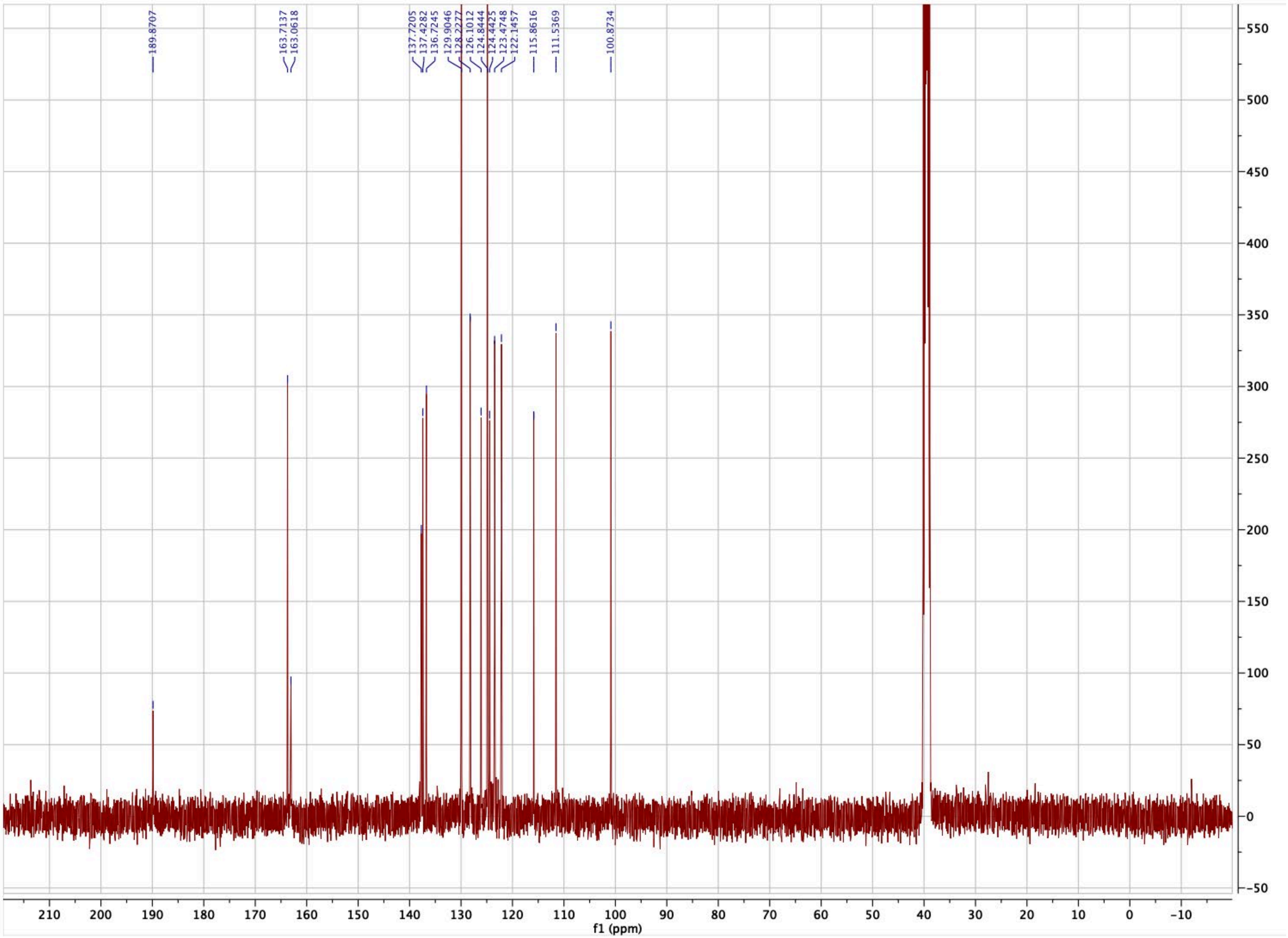

Figure S42. ^{13}C NMR Spectrum for compound **7d**

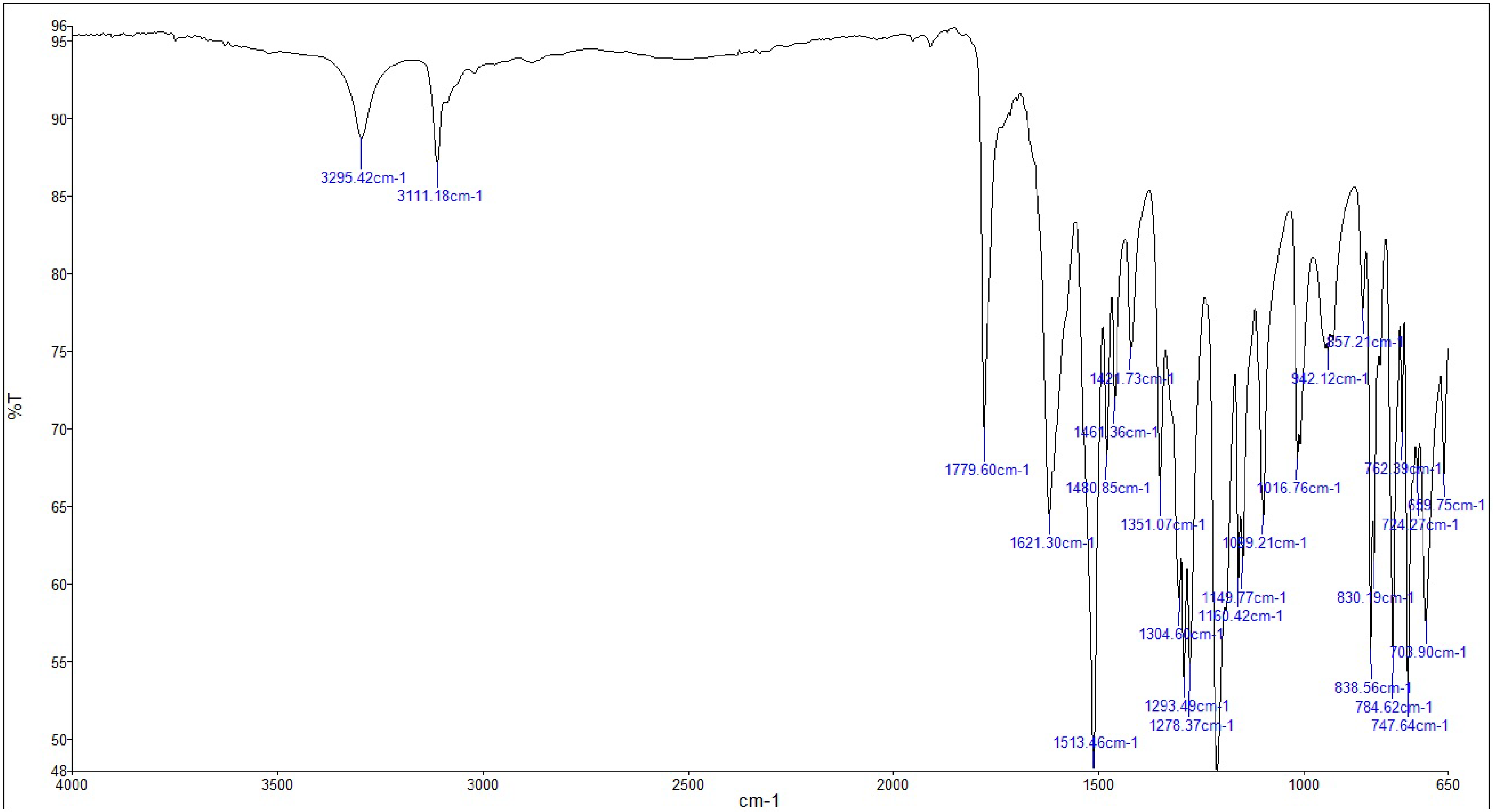

Figure S43. FTIR Spectrum for compound **7e**

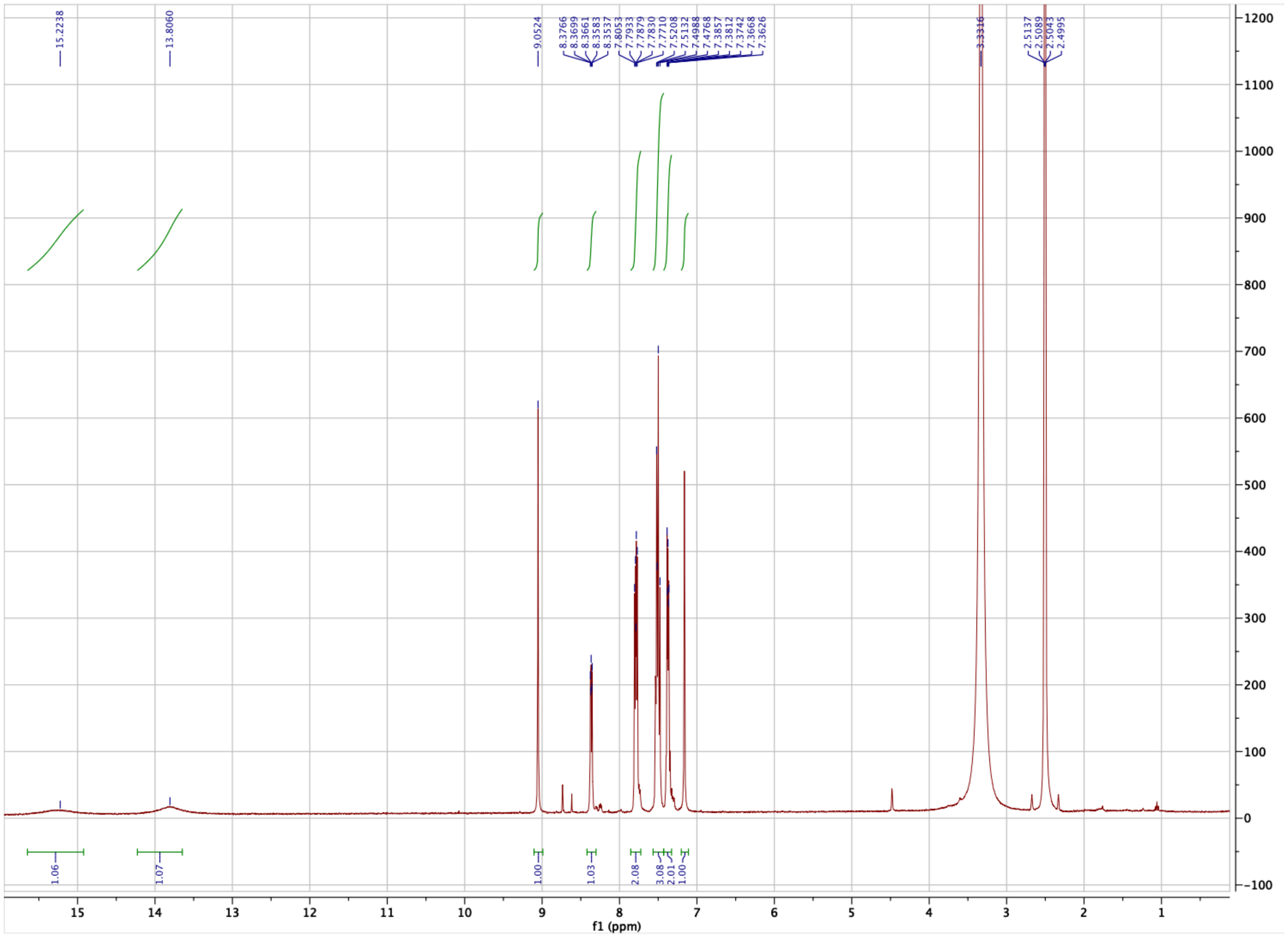

Figure S44. ^{1}H NMR Spectrum for compound **7e**

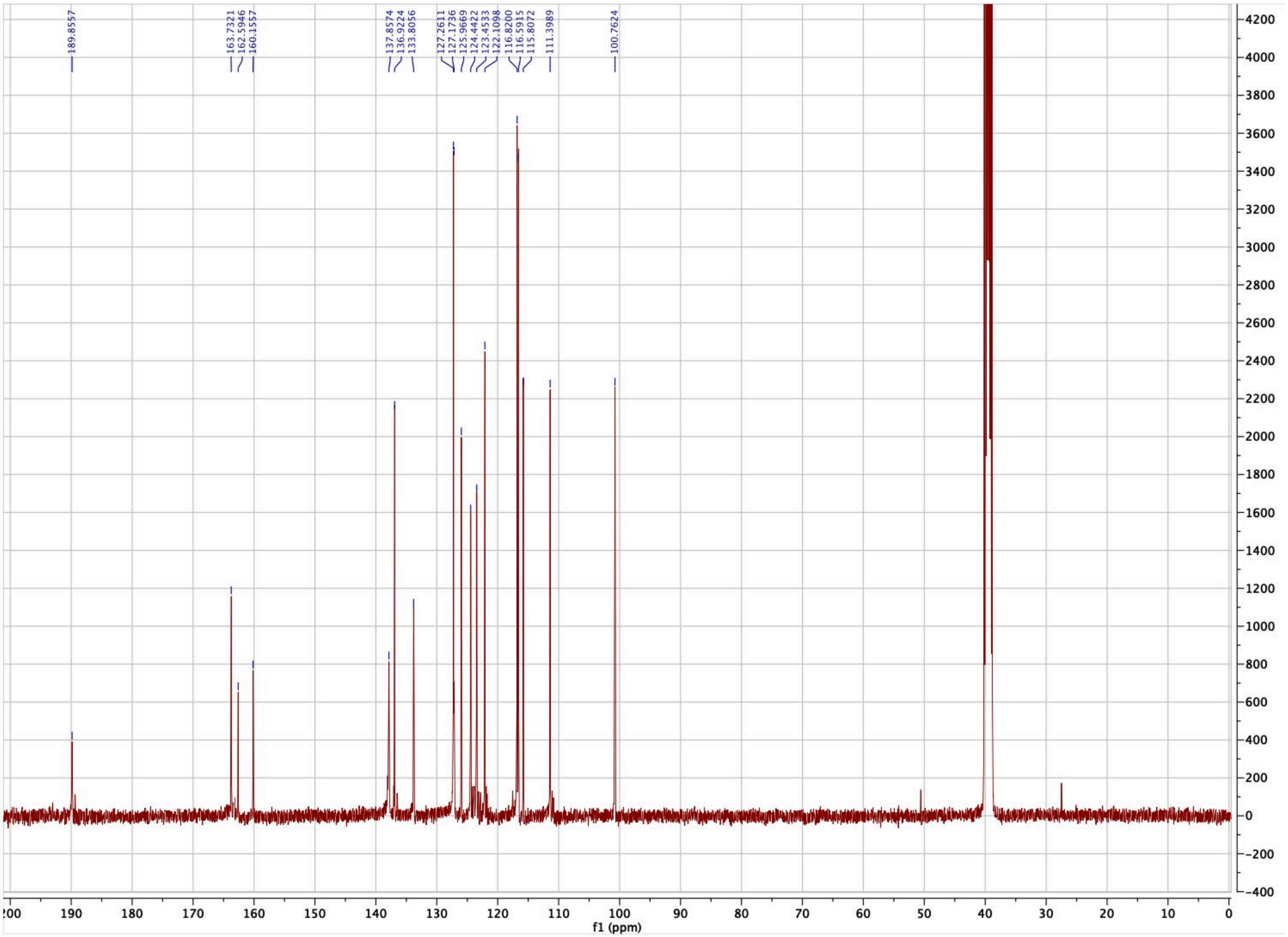

Figure S45. ^{13}C NMR Spectrum for compound **7e**

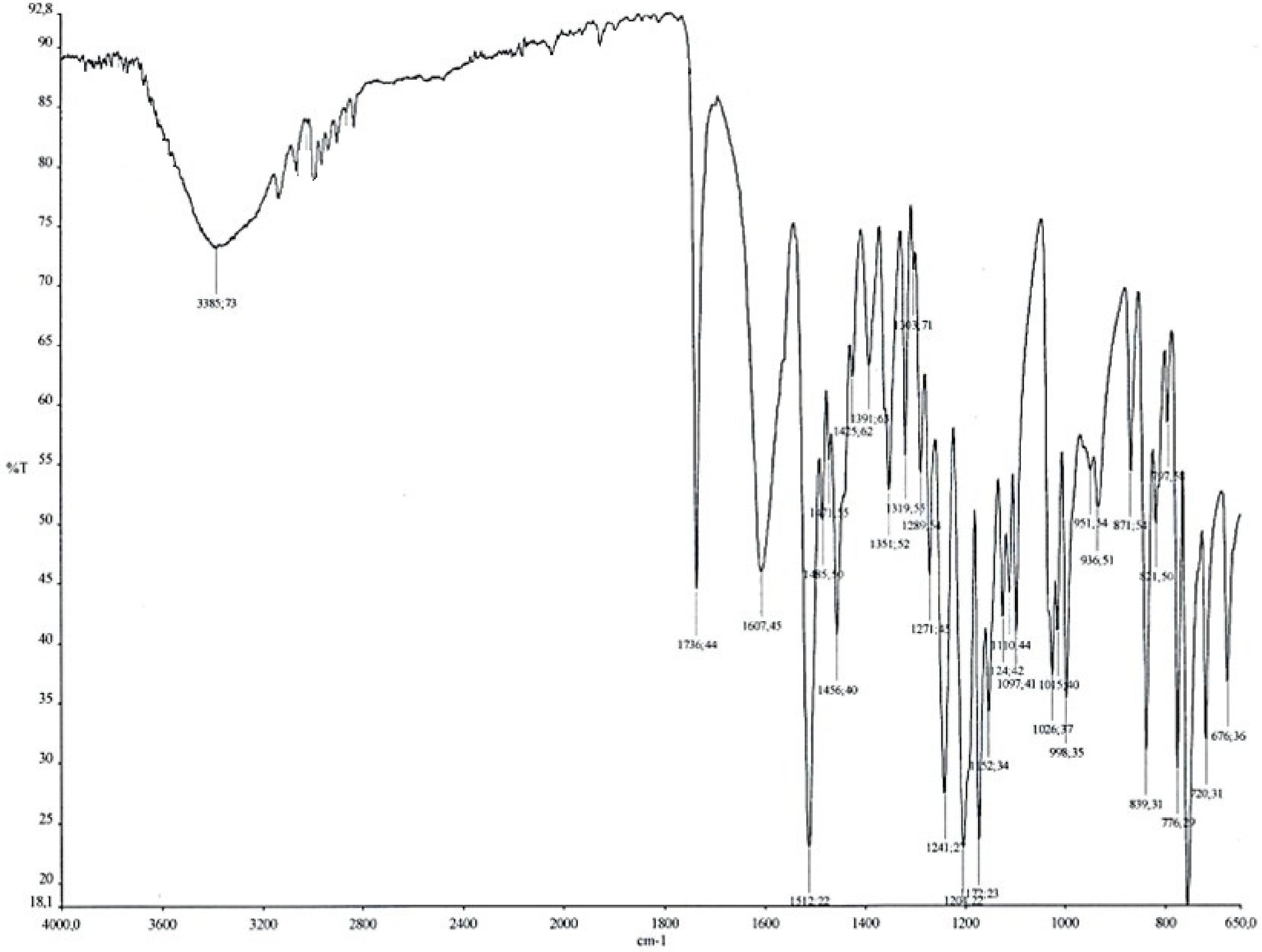

Figure S46. FTIR Spectrum for compound **8a**

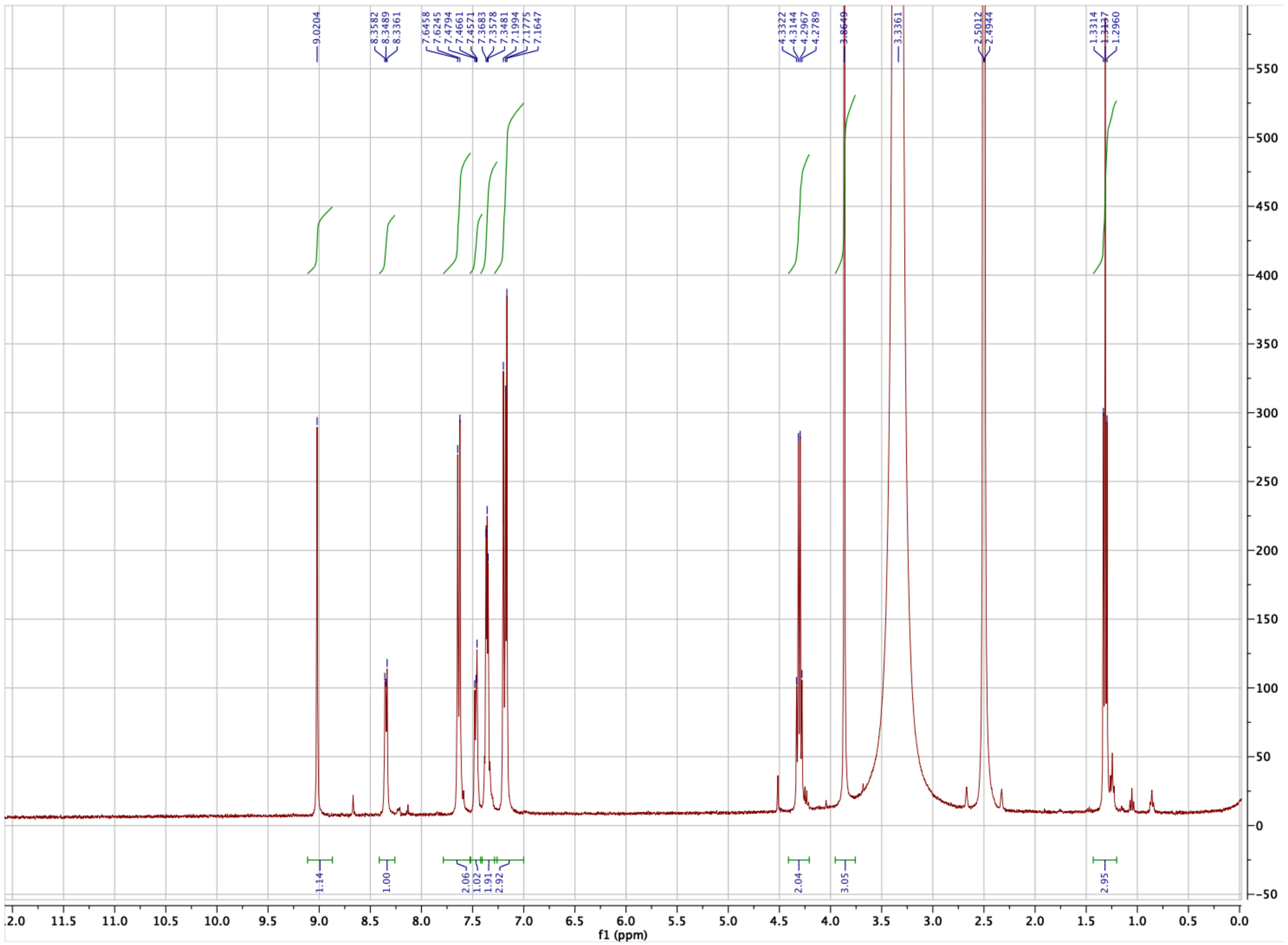

Figure S47. ^{1}H NMR Spectrum for compound **8a**

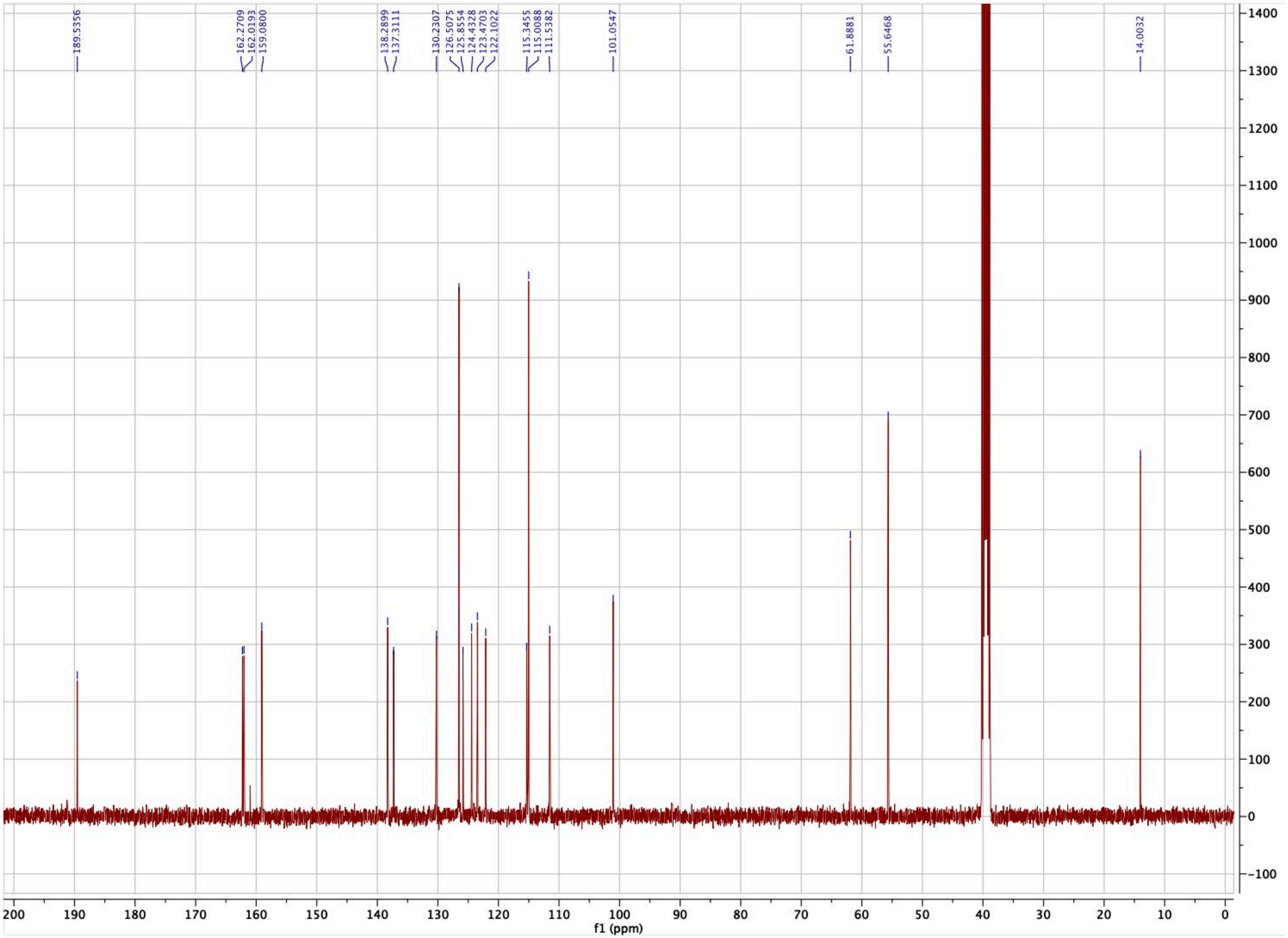

Figure S48. ^{13}C NMR Spectrum for compound **8a**

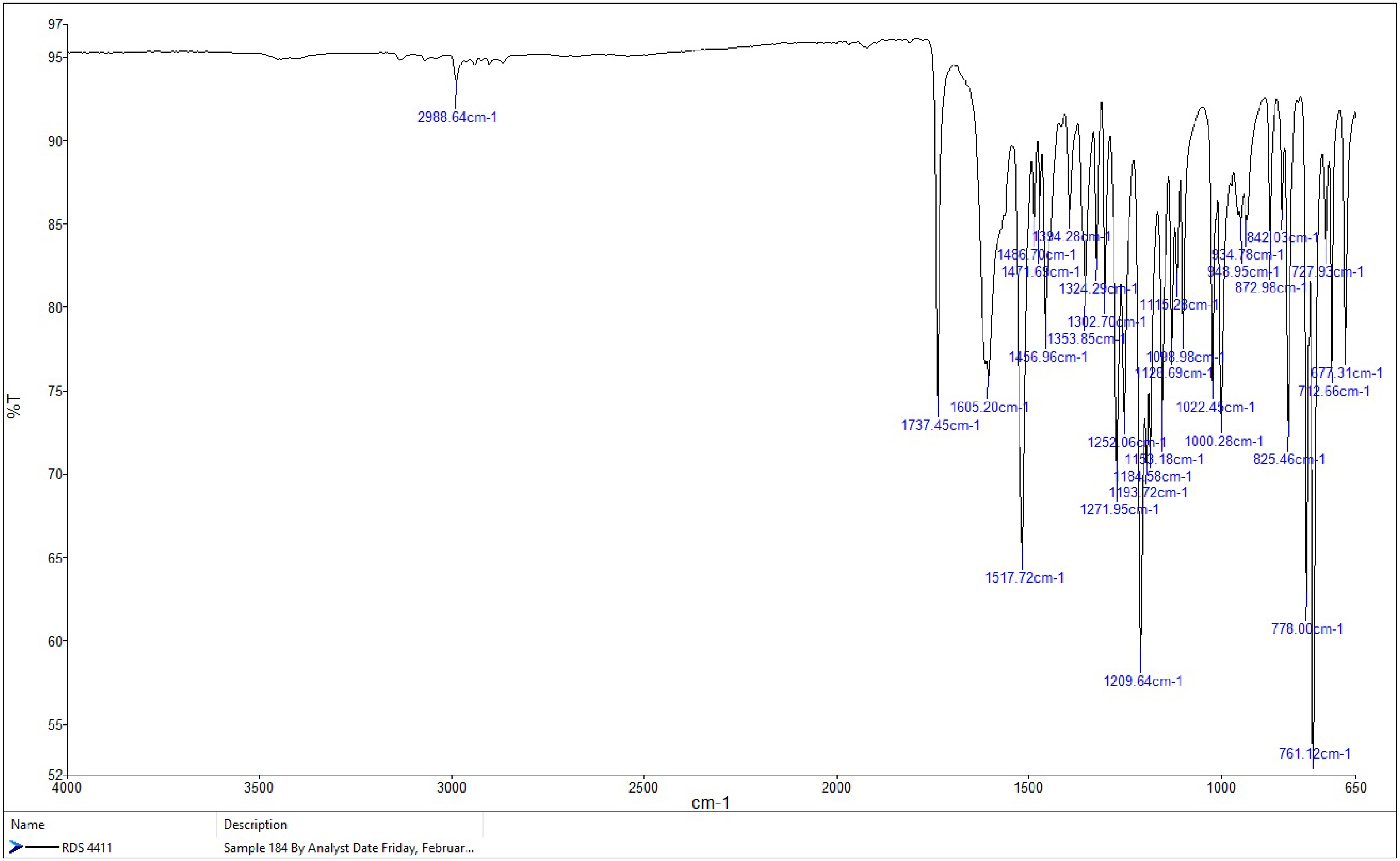

Figure S49. FTIR Spectrum for compound **8b**

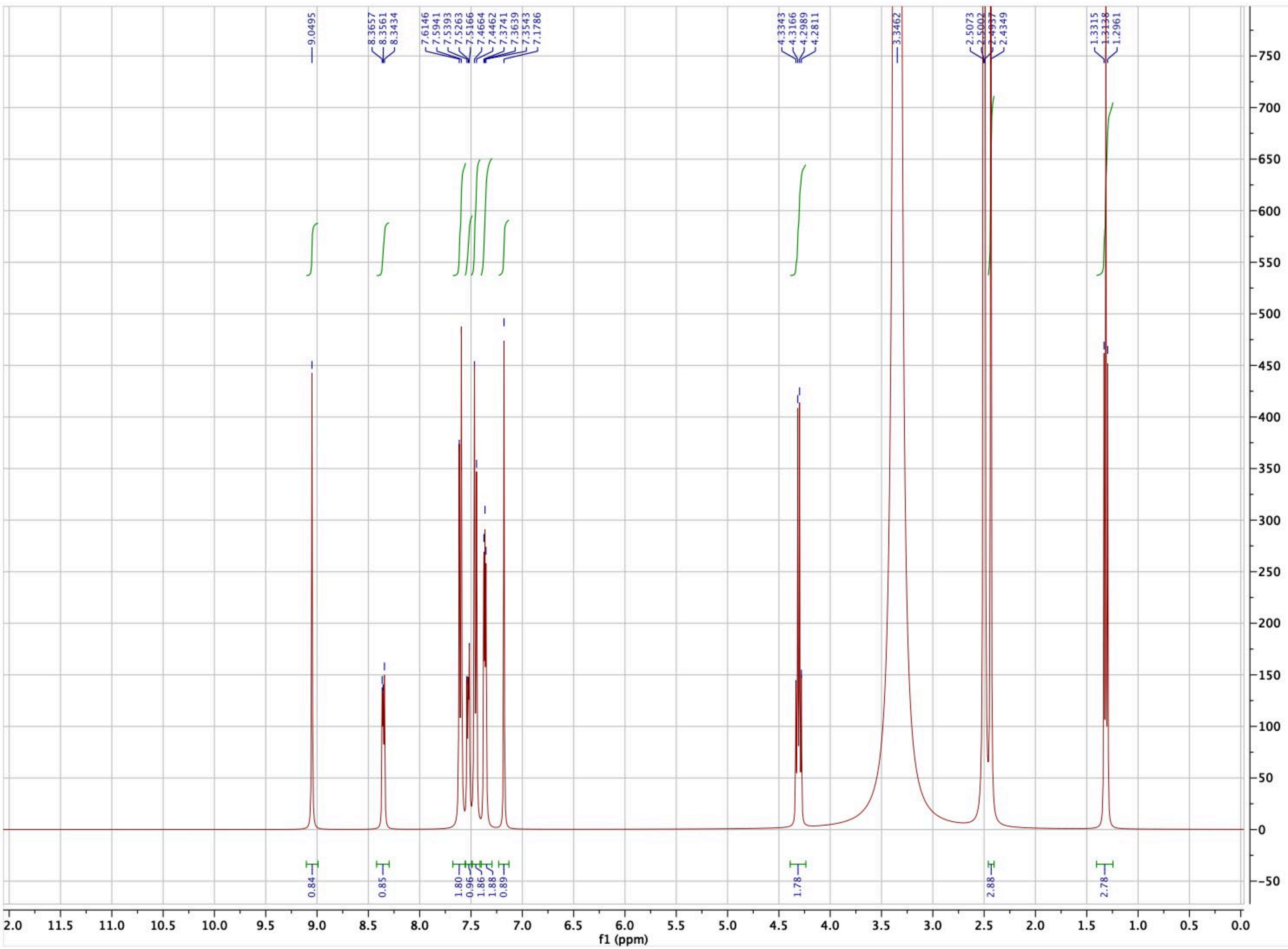

Figure S50. ^{1}H NMR Spectrum for compound **8b**

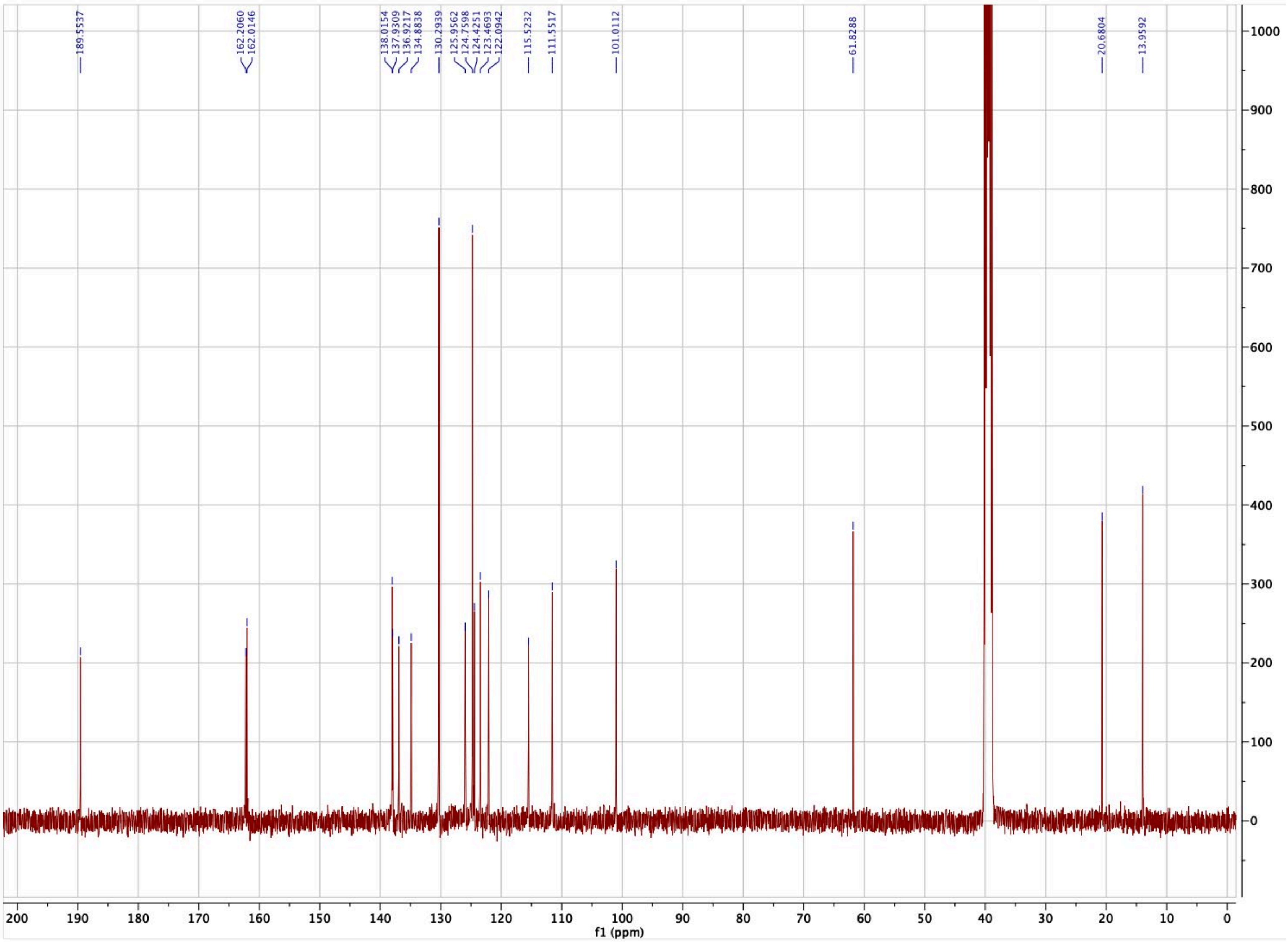

Figure S51. ^{13}C NMR Spectrum for compound **8b**

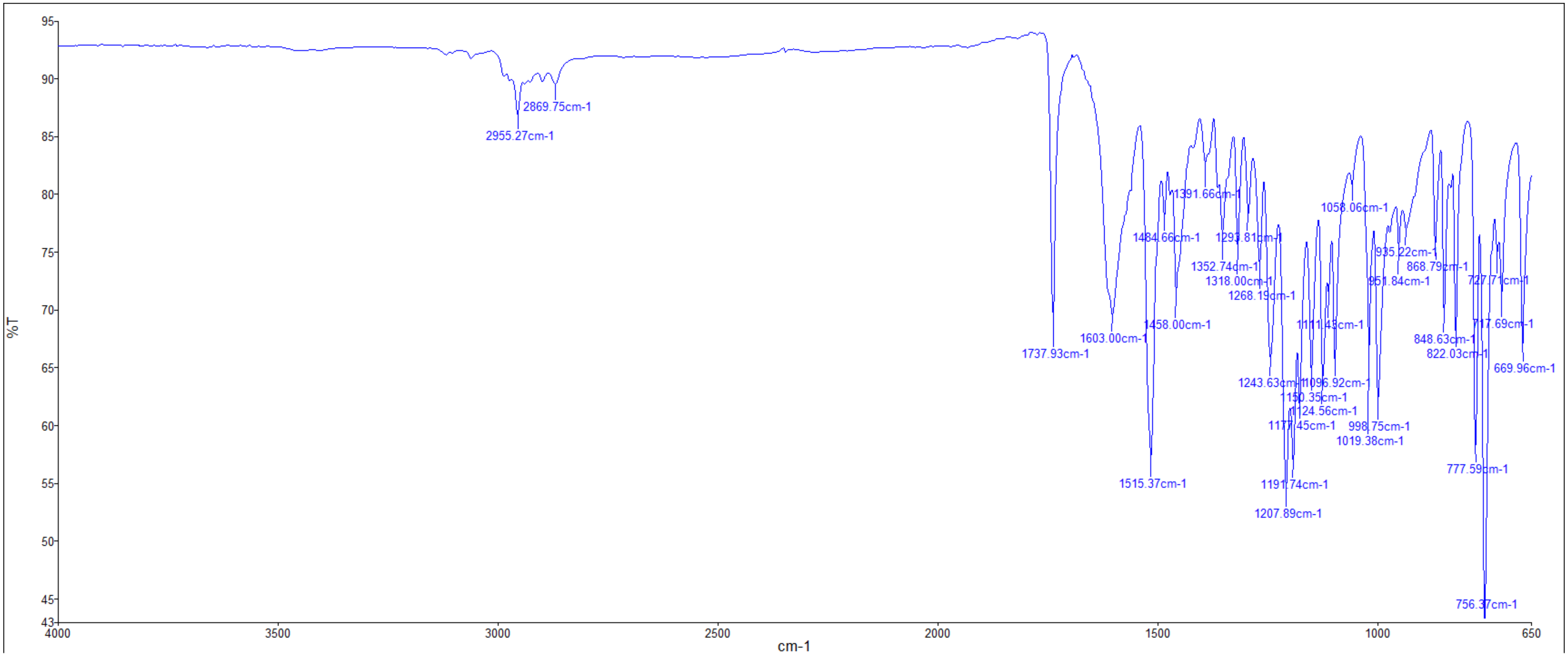

Figure S52. FTIR Spectrum for compound **8c**

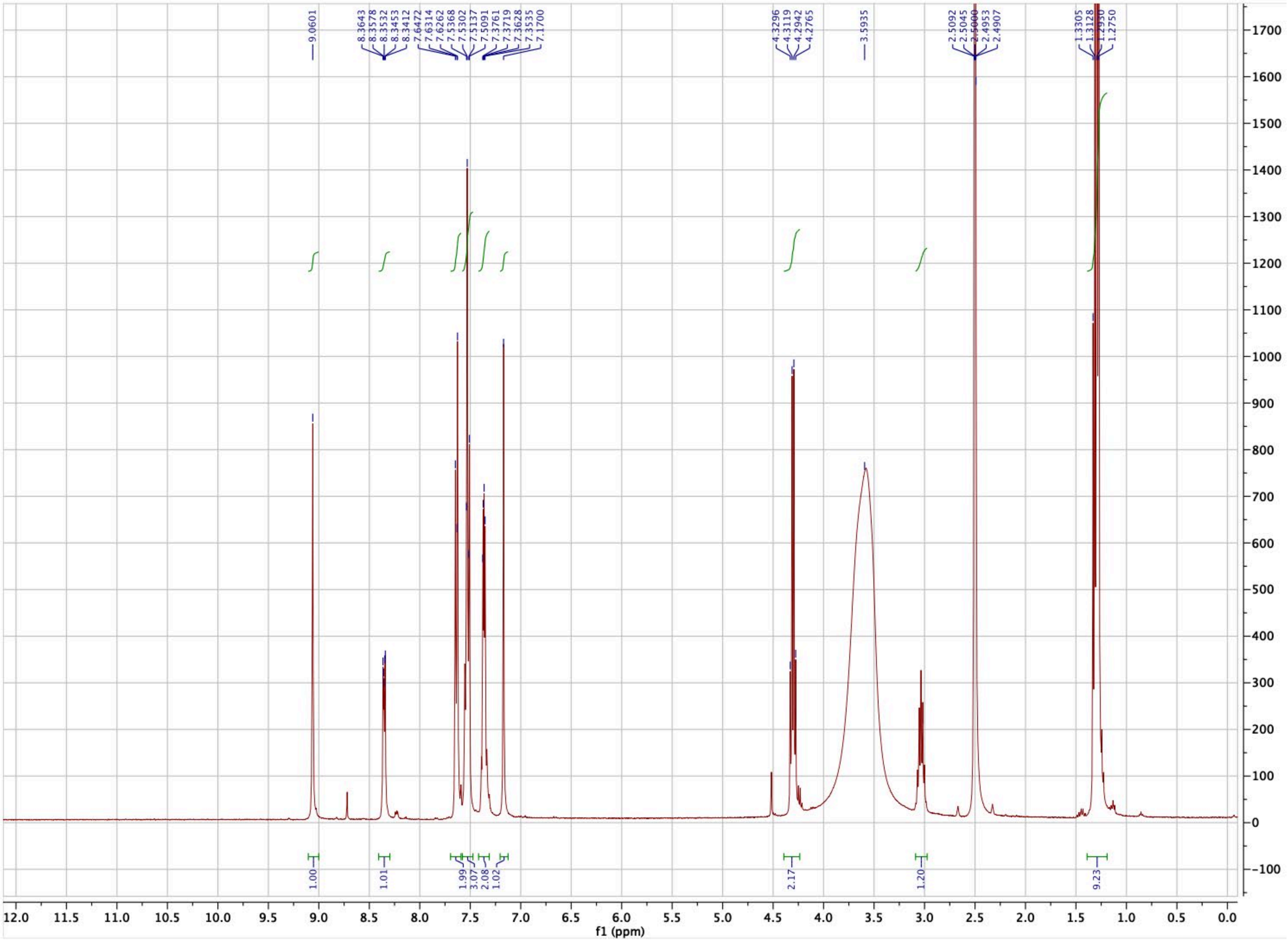

Figure S53. ^{1}H NMR Spectrum for compound **8c**

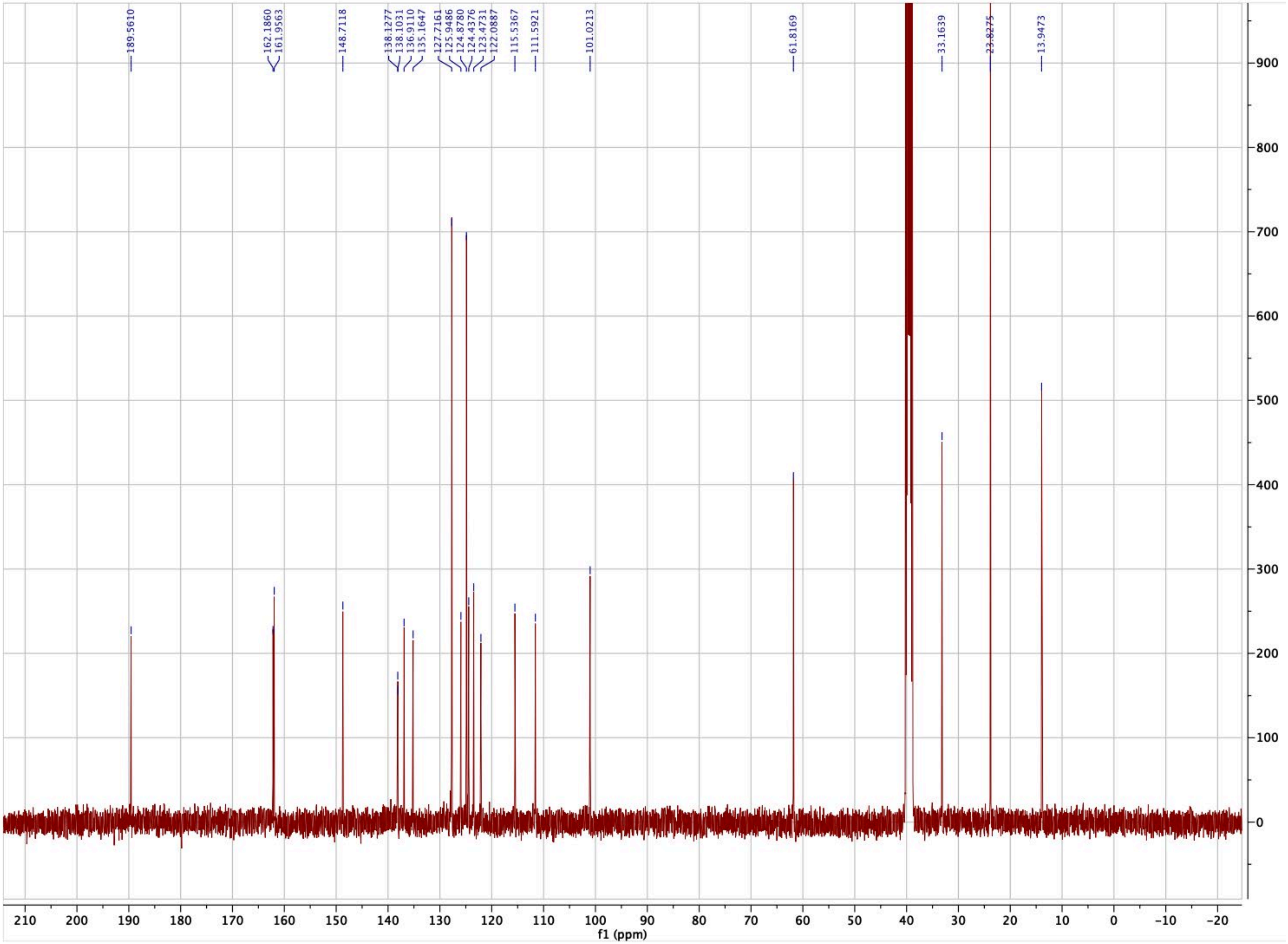

Figure S54. ^{13}C NMR Spectrum for compound **8c**

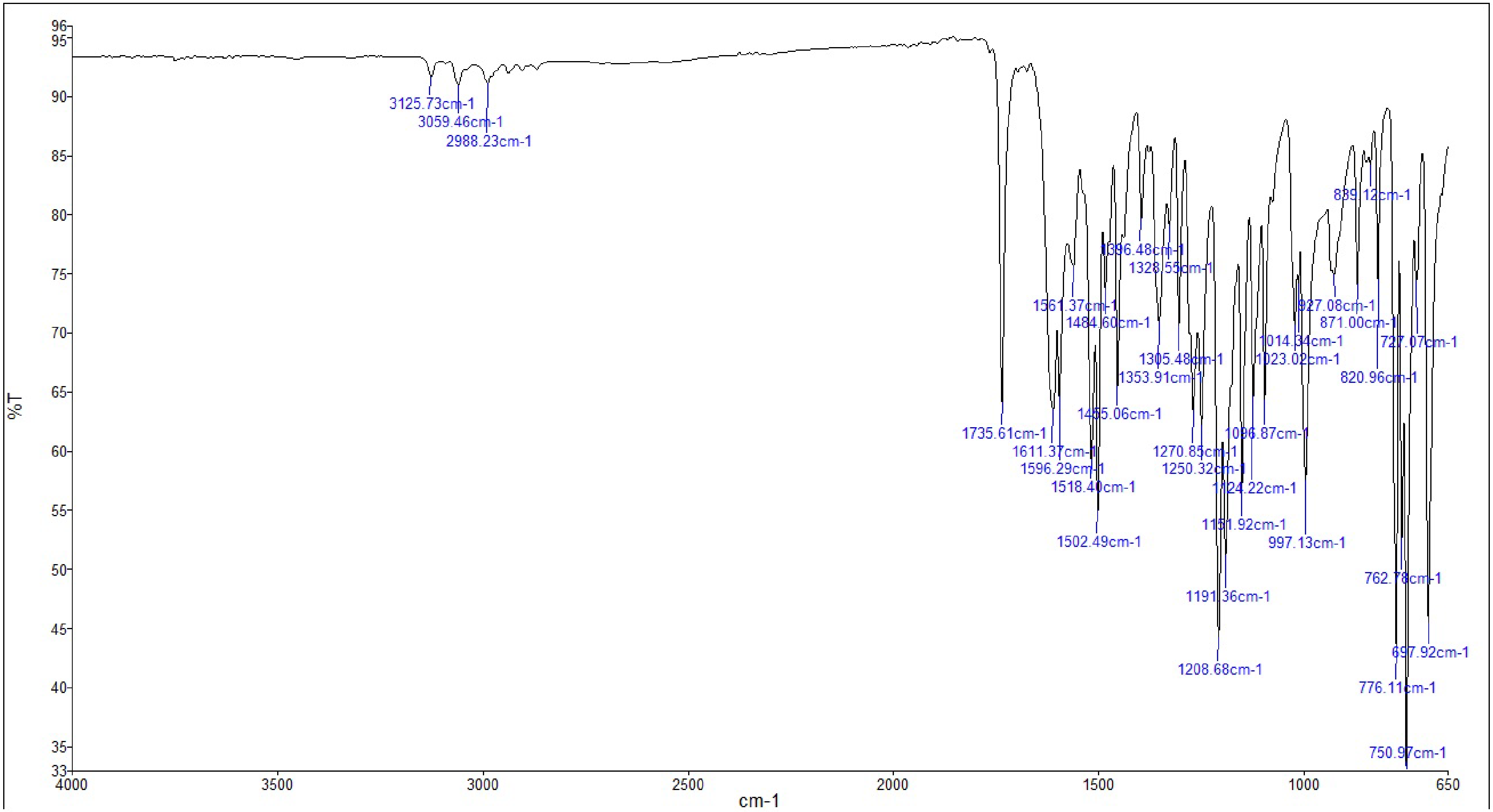

Figure S55. FTIR Spectrum for compound **8d**

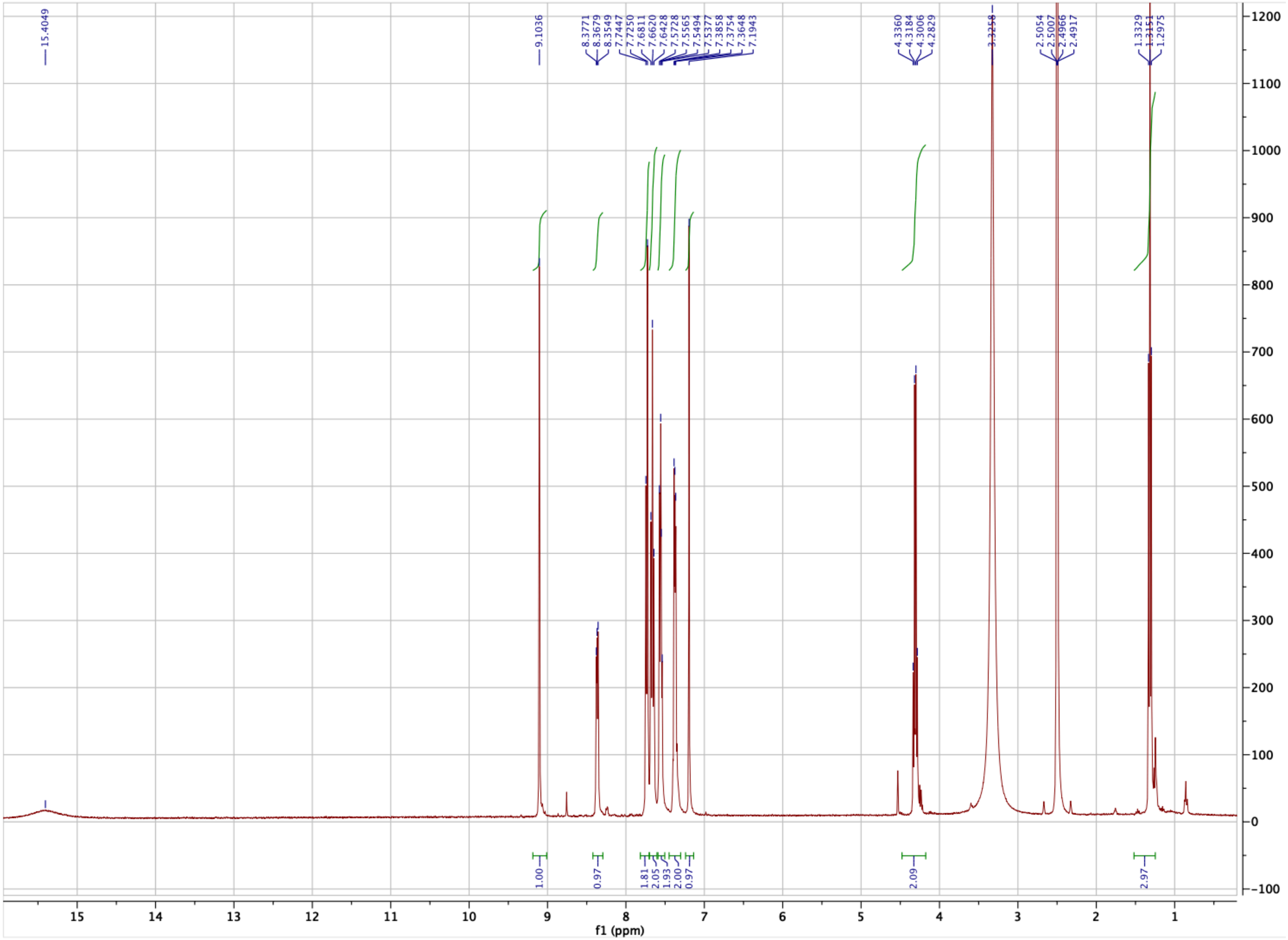

Figure S56. ^{1}H NMR Spectrum for compound **8d**

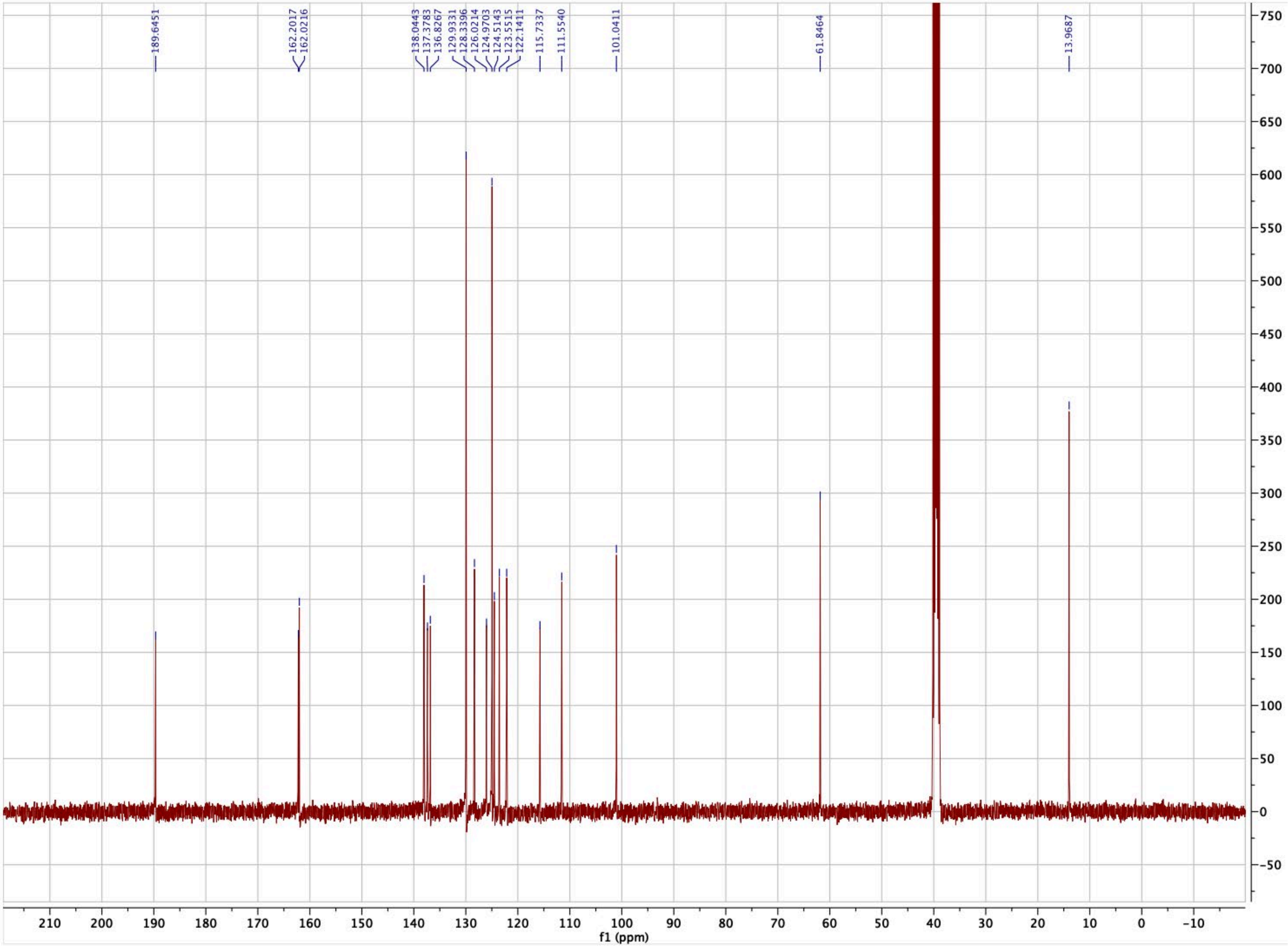

Figure S57. ^{13}C NMR Spectrum for compound **8d**

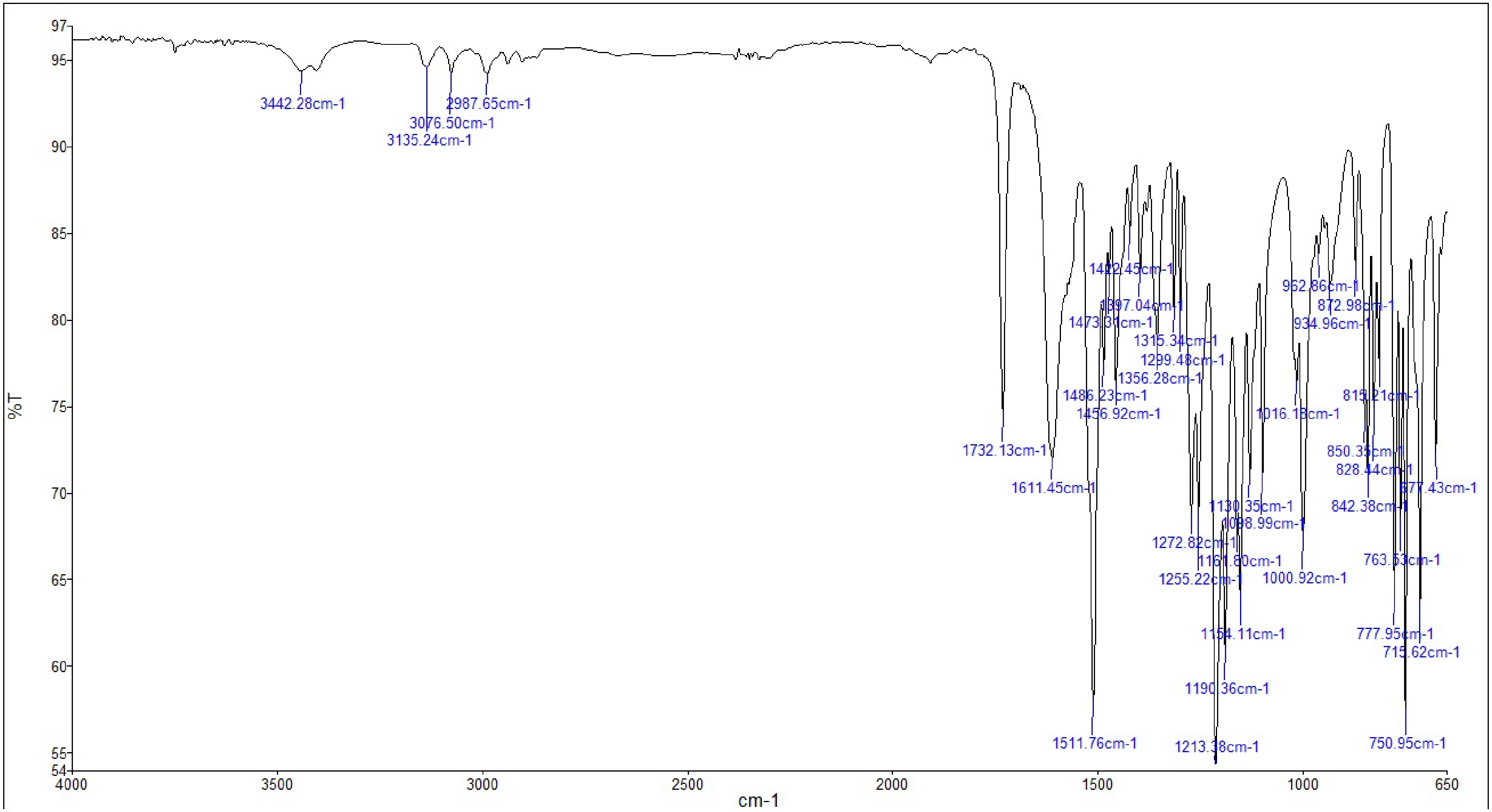

Figure S58. FTIR Spectrum for compound **8e**

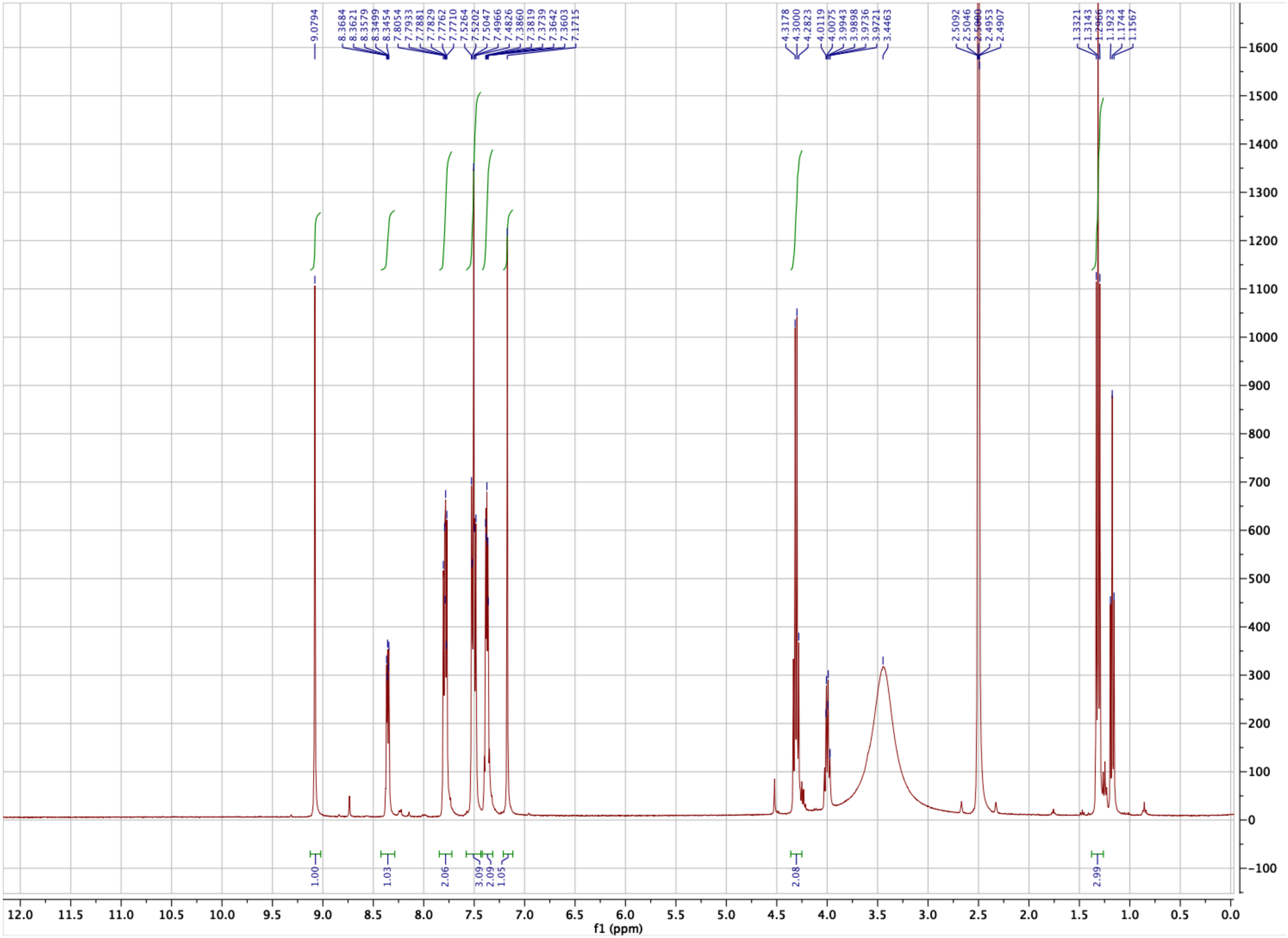

Figure S59. ^{1}H NMR Spectrum for compound **8e**

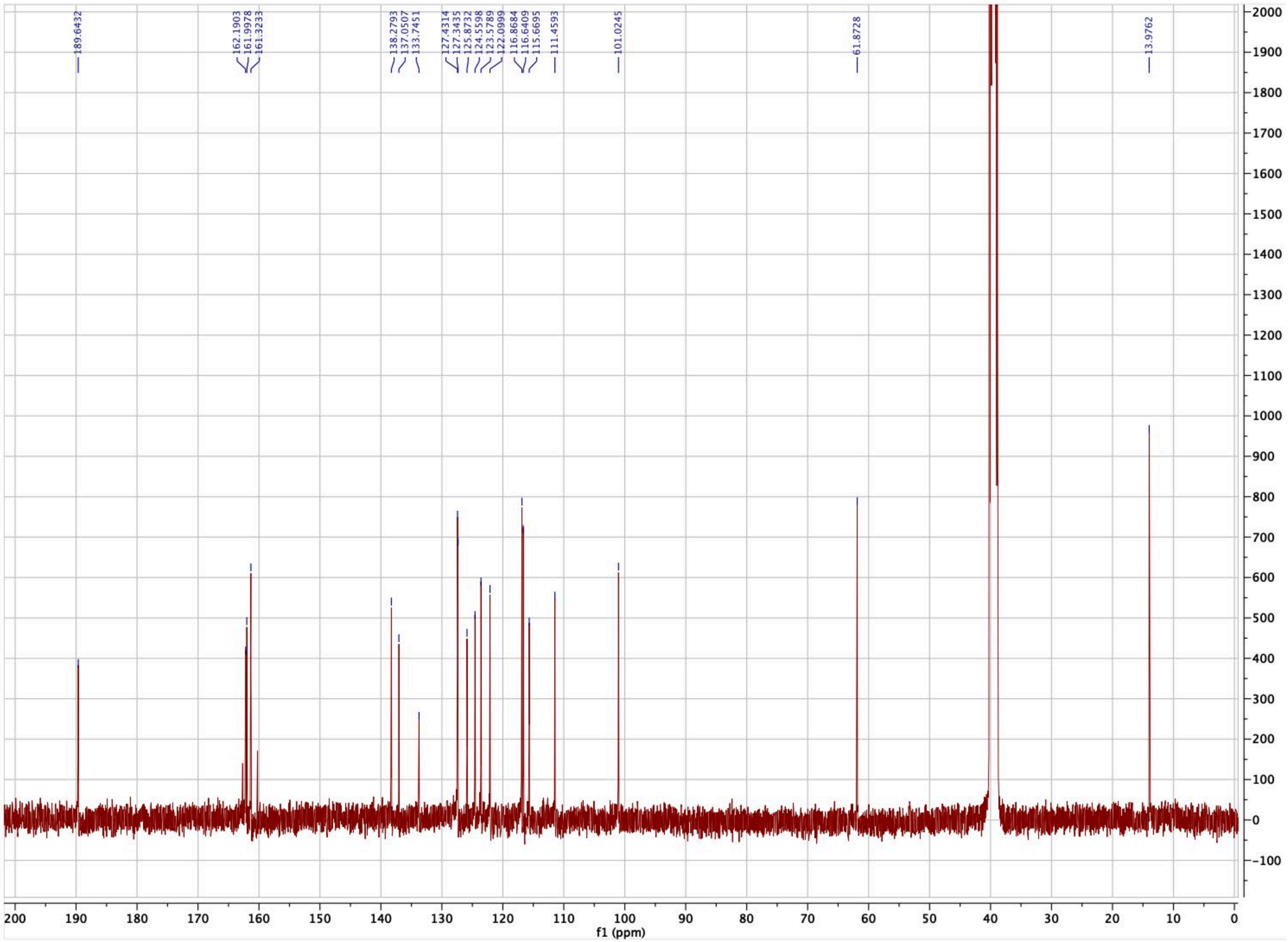

Figure S60. ^{13}C NMR Spectrum for compound **8e**

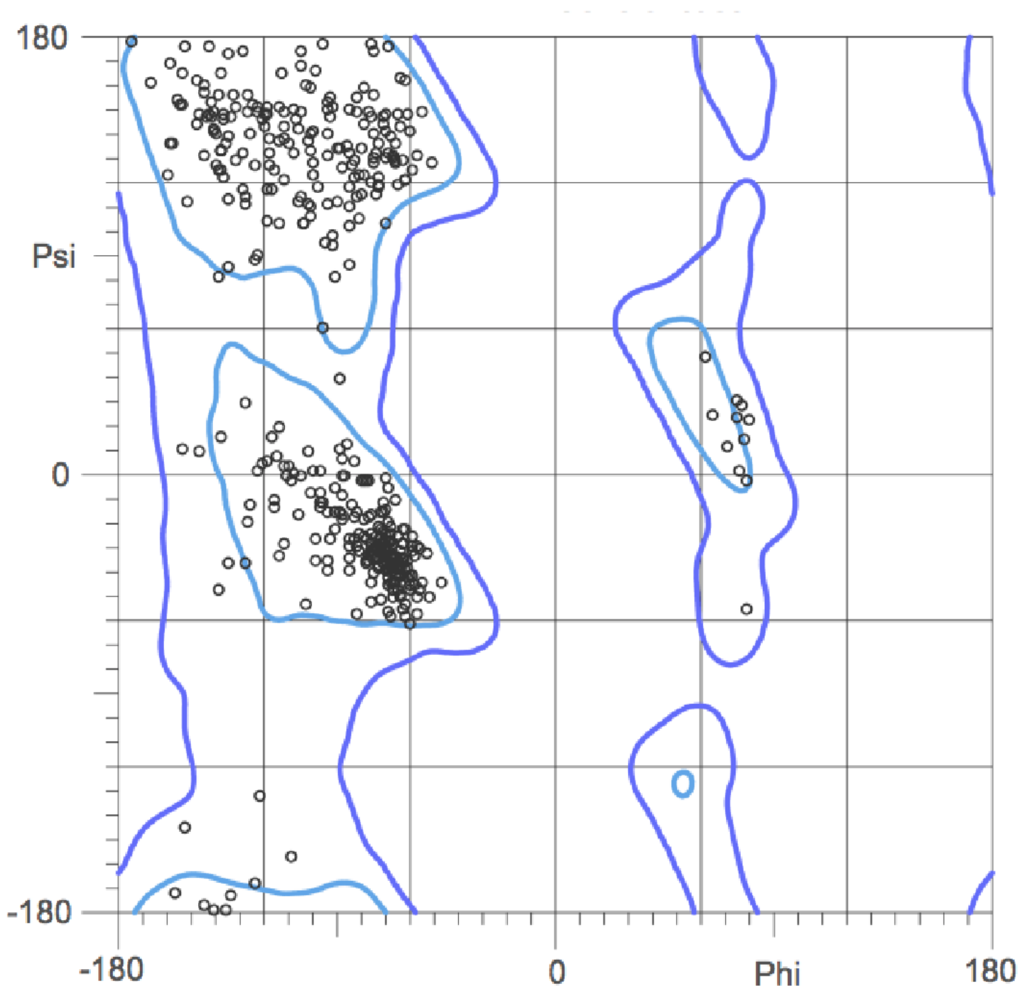

Figure S61. MolProbity[6] performed a Ramachandran plot analysis, revealing that 95.6% (570/596) of all residues were in favored (98%) regions. 100.0% (596/596) of all residues were in allowed (>99.8%) regions. There were no outliers. As a result, the model was validated as being of good quality and was used for computational simulation of ligand-receptor interactions.

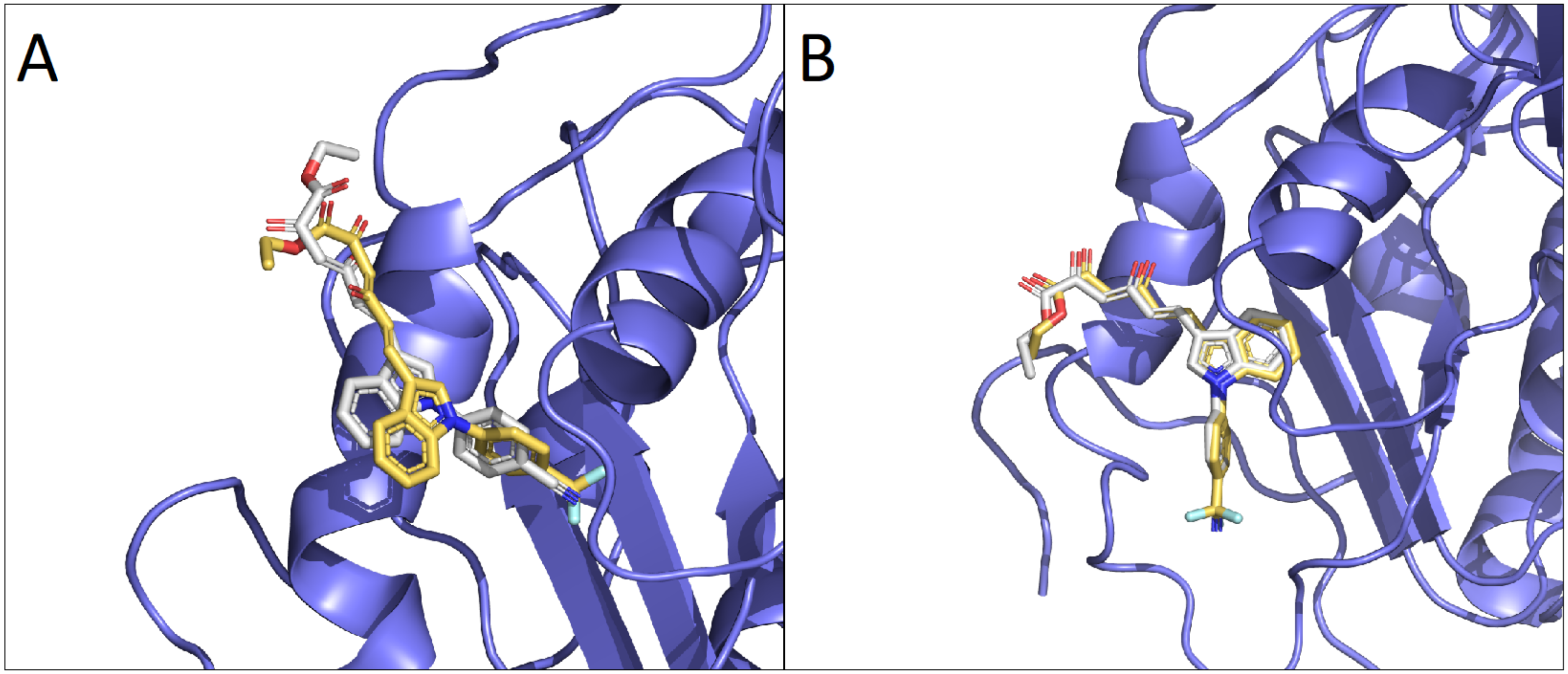

Figure S62. Predicted binding modes for **6h** (yellow) and **6g** (white).
